# Adaptation of Lactobacillus plantarum to Ampicillin Involves Mechanisms That Maintain Protein Homeostasis

**DOI:** 10.1128/mSystems.00853-19

**Published:** 2020-01-28

**Authors:** Chenxia Cao, Jicheng Wang, Yangshuo Liu, Lai-Yu Kwok, Heping Zhang, Wenyi Zhang

**Affiliations:** a Key Laboratory of Dairy Biotechnology and Engineering, Ministry of Education, Inner Mongolia Agricultural University, Huhhot, China; b Key Laboratory of Dairy Products Processing, Ministry of Agriculture, Inner Mongolia Agricultural University, Huhhot, China; Luxembourg Centre for Systems Biomedicine

**Keywords:** *Lactobacillus plantarum* P-8, adaptive laboratory evolution, ampicillin, proteomics, parallel reaction monitoring, *Lactobacillus*, antibiotics

## Abstract

The widespread use of antibiotics has caused great concern in the biosafety of probiotics. In this study, we conducted a 12-month adaptive laboratory evolution (ALE) experiment to select for antibiotics-adapted Lactobacillus plantarum P-8, a dairy-originated probiotic bacterium. During the ALE process, the ampicillin MIC for the parental L. plantarum P-8 strain increased gradually and reached the maximum level of bacterial fitness. To elucidate the molecular mechanisms underlying the ampicillin-resistant phenotype, we comparatively analyzed the genomes and proteomes of the parental strain (L. plantarum P-8) and two adapted lines (L. plantarum 400g and L. plantarum 1600g). The adapted lines showed alterations in their carbon, amino acid, and cell surface-associated metabolic pathways. Then, gene disruption mutants were created to determine the role of six highly expressed genes in contributing to the enhanced ampicillin resistance. Inactivation of an ATP-dependent Clp protease/the ATP-binding subunit ClpL, a small heat shock protein, or a hypothetical protein resulted in partial but significant phenotypic reversion, confirming their necessary roles in the bacterial adaptation to ampicillin. Genomic analysis confirmed that none of the ampicillin-specific differential expressed genes were flanked by any mobile genetic elements; thus, even though long-term exposure to ampicillin upregulated their expression, there is low risk of spread of these genes and adapted drug resistance to other bacteria via horizontal gene transfer. Our study has provided evidence of the biosafety of probiotics even when used in the presence of antibiotics.

**IMPORTANCE** Antibiotic resistance acquired by adaptation to certain antibiotics has led to growing public concerns. Here, a long-term evolution experiment was used together with proteomic analysis to identify genes/proteins responsible for the adaptive phenotype. This work has provided novel insights into the biosafety of new probiotics with high tolerance to antibiotics.

## Abstract (precis)

Antibiotic resistance acquired by adaptation to certain antibiotics has led to growing public concerns. Here, a long-term evolution experiment was used together with proteomic analysis to identify genes/proteins responsible for the adaptive phenotype. This work has provided novel insights into the biosafety of new probiotics with high tolerance to antibiotics.

## INTRODUCTION

The spread of antibiotic resistance in pathogenic bacteria has led to growing public concerns (1). Bacterial antibiotic resistance can be acquired during adaptive evolution via prolonged exposure to antibiotics (2). Several antimicrobial drugs that were once effective against bacterial infections had become ineffective (3). To slow bacterial acquisition and spread of antibiotic resistance, many countries have established policies and practices for rational use of antibiotics (4). Alternative or adjunct treatment has also been suggested to reduce antibiotic use (5). As revealed by a number of clinical studies, the use of probiotics appears to be a good choice of adjunct treatment for improving human gastrointestinal tract (GIT) disorders (6, 7).

Lactobacillus plantarum is a versatile probiotic bacterial species isolated from a traditional fermented dairy product (8) but is also naturally found in plant materials and in GITs of humans or animals (9, 10). Accumulating evidence has demonstrated that L. plantarum plays a vital role in host health (11). The physiological and cellular mechanisms of the beneficial effects of some probiotic strains have been elucidated. For example, L. paracasei F19 and L. plantarum 2362 enhance interleukin-6 production in immunologically stimulated enterocytes via a heat shock-dependent mechanism (12); L. plantarum 423 and Enterococcus mundtii ST4SA act against listeria via bacteriocin production and cell adhesion (13); L. plantarum PFM 105 promotes gut development by modulating the gut microbiota of weaning piglets (14). Moreover, some strains can produce bacteriocins such as plantaricin that enhance the effectiveness of L. plantarum against human GIT infections (15). These properties make L. plantarum a good candidate for use in adjunct treatment of diseases such as GIT infections.

Although probiotics are generally considered safe, as coadministration of probiotics and antibiotics is becoming a trend in clinical practice, whether frequent and prolonged exposure to antibiotics would enhance the evolution of antibiotic resistance and promote the spread of antibiotic resistance genes is still a valid concern (16, 17). To date, however, no study has used high-throughput genomic research tools to assess the biosafety risk of coadministration of probiotics and antibiotics. Thus, the drug resistance mechanisms and genotype-phenotype correlations of adapted mutants of probiotics that are involved in the course of antibiotics-driven adaptation remain unknown.

In this study, we conducted an adaptive laboratory evolution (ALE) experiment by continuously cultivating L. plantarum P-8 in the presence of ampicillin for 12 months to generate ampicillin-resistant strains. Ampicillin was chosen to provide the selection pressure in the ALE experiment due to its frequent clinical use (18). The ampicillin-resistant strains were analyzed by an integrative genomic, proteomic, and reverse genetic approach to elucidate the cellular mechanism of antibiotic adaptation of L. plantarum P-8 in the course of the ALE.

## RESULTS

### Long-term evolution of L. plantarum P-8 under control and ampicillin administration conditions.

**FIG 1:**
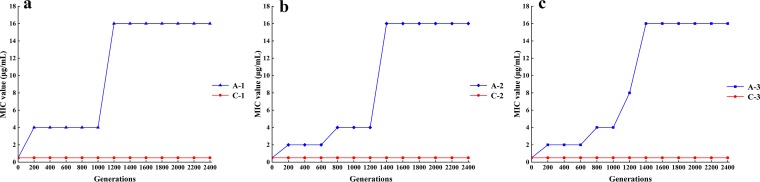
Changes in the drug MICs for L. plantarum P-8 during the 12-month adaptive laboratory evolution experiment. A-1 and C-1, A-2 and C-2, and A-3 and C-3 represent bacterial line 1, line 2, and line 3, respectively. The three cell lines were derived from three individual colonies as indicated. Each assay was repeated three times.

The evolution of L. plantarum P-8 under control and ampicillin conditions was followed in parallel for 2,400 bacterial generations. The only difference between the control and ampicillin conditions was that ampicillin was not added in the culture medium of the control. The ampicillin MIC was employed as an indicator to monitor changes in the level of fitness of bacteria grown under conditions of two different experimental environments. At generation zero, the ampicillin MIC (0.5 μg/ml) was the same for all bacterial lines. The MIC for the bacterial lines (A-1, A-2, and A-3) increased in the ampicillin-containing environment and climbed until it reached a maximum value of 16 μg/ml after 1,600 bacterial generations (Fig. 1), while the MIC for the bacteria cultured under the control condition (C-1, C-2, and C-3) remained unchanged (Fig. 1), suggesting an antibiotics-dependent enhancement of fitness.

### Selection of adapted strains for genomic and proteomic analyses.

To explore the mechanism of adaptive evolution of ampicillin resistance, the genome and proteome of the L. plantarum P-8 parental strain were compared with those of two descendant strains of bacterial line A-1, which were adapted to ampicillin for 400 and 1,600 bacterial generations (here named L. plantarum 400g and L. plantarum 1600g, respectively). L. plantarum 400g and L. plantarum 1600g were selected at time points representing the middle and late stages of the ALE. These two time points (400 and 1600 generations) were selected because they were the next time points when obvious phenotypic changes occurred (at 200 and 1,400 generations, respectively).

Both L. plantarum 400g and L. plantarum 1600g grew slower and entered stationary phase later than L. plantarum P-8 when cultivated in the presence of ampicillin (see Fig. S1 in the supplemental material). The pH values of the culture supernatants of L. plantarum P-8 dropped earlier than those of L. plantarum 400g and L. plantarum 1600g during growth (Fig. S1a). Meanwhile, L. plantarum 400g and L. plantarum 1600g exhibited higher antibiotic resistance, as reflected by the higher viable counts and optical density (OD) values at the late growth stage (Fig. S1b and c).

10.1128/mSystems.00853-19.1 FIG S1 Growth curves of L. plantarum P-8, L. plantarum 400g, and L. plantarum 1600g cultivated in ampicillin-containing LSM. Changes in the pH (a), viable counts (b), and optical density at 600 nm (OD_600_) (c) were monitored over 30 h. Each assay was repeated three times. Error bars represent standard deviations. Download FIG S1, TIF file, 0.3 MB. Copyright © 2020 Cao et al. 2020 Cao et al. This content is distributed under the terms of the Creative Commons Attribution 4.0 International license.

### Identification of single nucleotide polymorphisms (SNPs) in the selected adapted strains by whole-genome resequencing.

**FIG 2:**
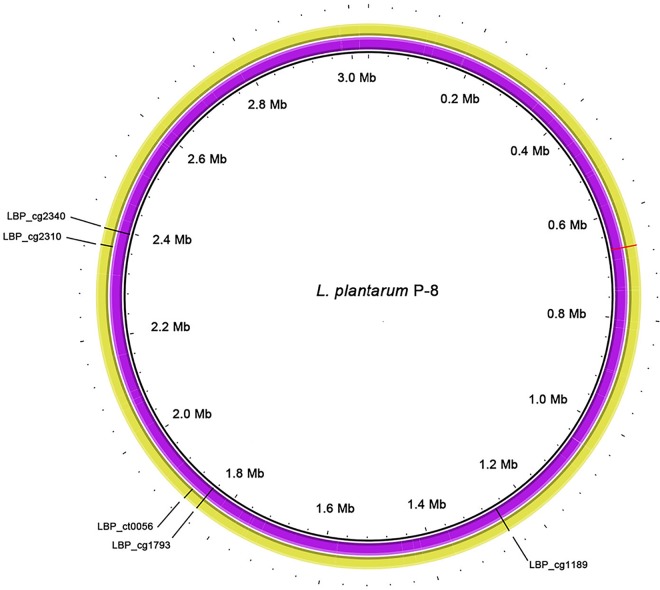
Distribution of SNPs on the genomes of L. plantarum 400g and L. plantarum 1600g. The inner circle illustrates the nucleotide position of the genome. The violet and yellow circles represent the genomes of L. plantarum 400g and L. plantarum 1600g, respectively. Specific genes are denoted by gene locus tags. The mutation occurring at an intergenic region is indicated in red.

A total of six SNPs were identified in L. plantarum 400g and L. plantarum 1600g in comparison with the genome of L. plantarum P-8 (Fig. 2; see also Table S1 in the supplemental material). Four of them were nonsynonymous SNPs. Interestingly, two nonsynonymous SNPs were found within two genes (LBP_cg1189 and LBP_cg1793); both genes encoded penicillin-binding protein 2B (PBP2B) and were found to be involved in cell wall biosynthesis.

10.1128/mSystems.00853-19.3 TABLE S1 General information describing SNPs in *L*. *plantarum* strains 400g and 1600g in comparison with L. plantarum P-8. Download Table S1, DOCX file, 0.02 MB. Copyright © 2020 Cao et al. 2020 Cao et al. This content is distributed under the terms of the Creative Commons Attribution 4.0 International license.

### Identification of differentially expressed proteins of selected adapted strains by proteomics.

The proteomes of L. plantarum P-8, L. plantarum 400g, and L. plantarum 1600g were comparatively analyzed to characterize the bacterial adaptation to ampicillin exposure at the protein level. Peptides prepared from three biological replicates of each strain were labeled with tandem mass tag 126 (TMT-126), TMT-127N, TMT-127C, TMT-128N, TMT-128C, TMT-129N, TMT-129C, TMT-130N, and TMT-130C reagents, respectively. A total of 68,341 peptide spectrum matches were found by TMT analysis, and 1,446 proteins were identified (Table S2). Proteins having a *P* value of <0.05 and fold change of >1.2 were considered significantly differential upregulated, while those having a *P* value of <0.05 and fold change of <0.83 were considered significantly differential downregulated.

10.1128/mSystems.00853-19.4 TABLE S2 General information of identified proteins. Download Table S2, DOCX file, 0.08 MB. Copyright © 2020 Cao et al. 2020 Cao et al. This content is distributed under the terms of the Creative Commons Attribution 4.0 International license.

**FIG 3:**
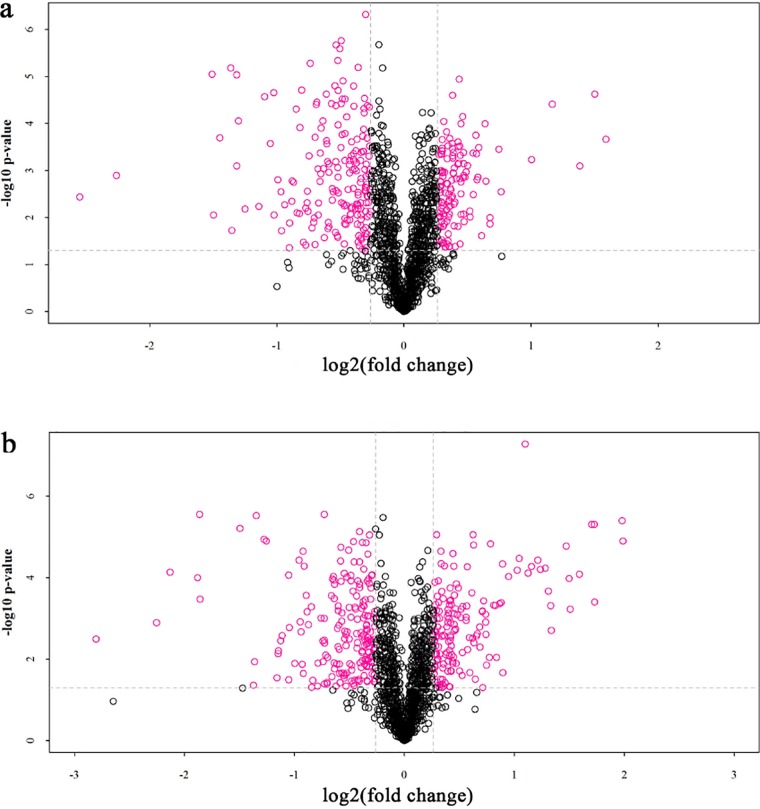
Volcano plot of the proteomic data of L. plantarum 400g (a) and L. plantarum 1600g (b). The *x* axis indicates the differential levels of protein expression (fold ratio between the parental L. plantarum P-8 and the adapted strains, 400g and 1600g, in a log-2 scale). The *y* axis indicates the statistical significance for the differential expression levels (*P* value generated from *t* test in log_10_ scale).

**FIG 4:**
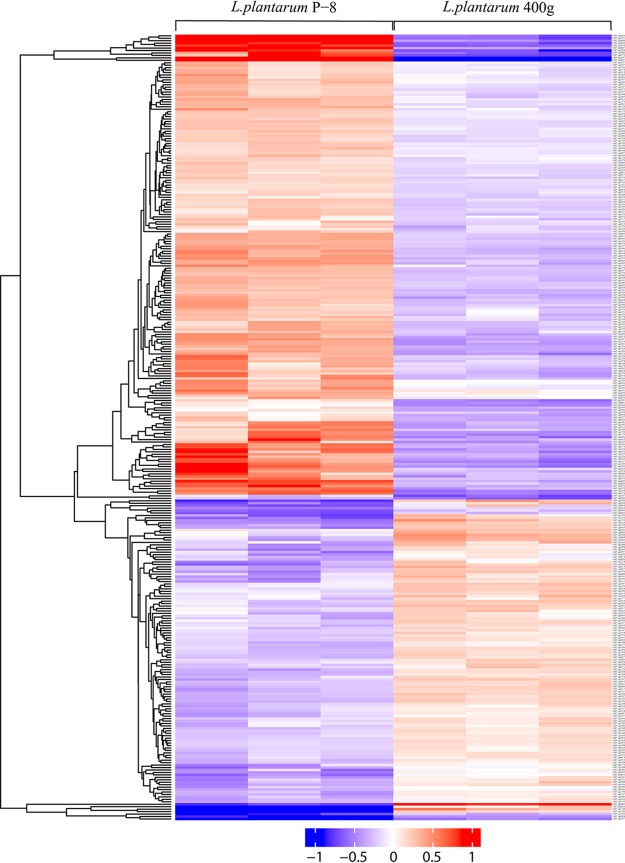
Hierarchical clustering of differentially expressed proteins of L. plantarum 400g relative to L. plantarum P-8. Each row represents a differentially expressed protein, while data of each of the three biological replicates are plotted in one column. The color scale represents the protein expression level, ranging from −1 (minimal expression) to 1 (maximal expression).

**FIG 5:**
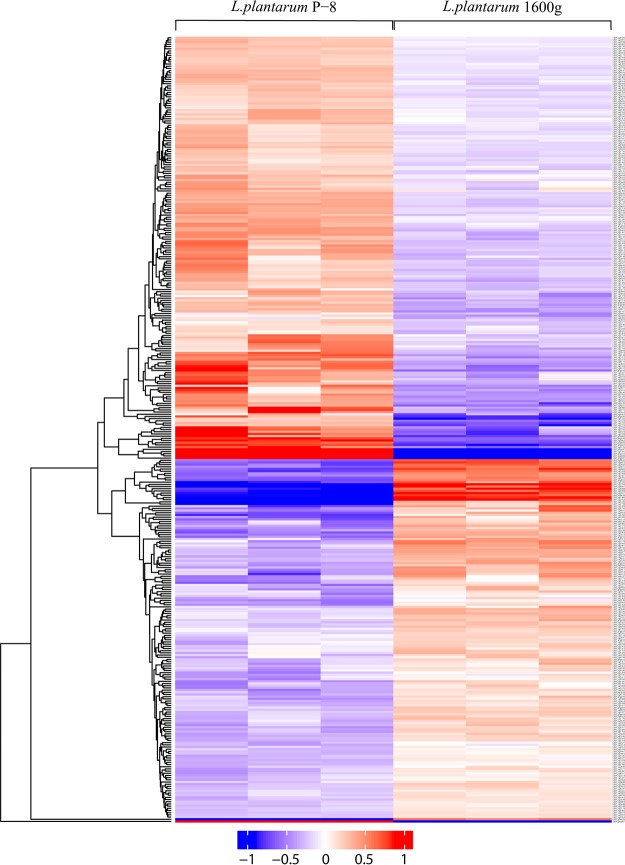
Hierarchical clustering of differentially expressed proteins of L. plantarum 1600g relative to L. plantarum P-8. Each row represents a differentially expressed protein, while data of each of the three biological replicates are plotted in one column. The color scale represents the protein expression level, ranging from −1 (minimal expression) to 1 (maximal expression).

The proteomic data were represented by volcano plots (Fig. 3). Overall, 395 and 214 differentially expressed proteins were identified in L. plantarum 400g and L. plantarum 1600g, respectively. For L. plantarum 400g, 165 proteins significantly increased in expression, while 230 proteins exhibited a significant decrease in expression (Fig. 4; see also Table S3 and Table S4). For L. plantarum 1600g, expression of 126 proteins significantly increased, while that of 88 proteins significantly decreased (Fig. 5; see also Table S5 and Table S6). Seventy-three proteins were commonly upregulated in both L. plantarum 400g and L. plantarum 1600g, while 140 proteins were commonly downregulated (Fig. S2).

10.1128/mSystems.00853-19.2 FIG S2 Venn plot showing distribution of differentially expressed proteins in L. plantarum 400g and L. plantarum 1600g. (a) Upregulated proteins. (b) Downregulated proteins. Download FIG S2, TIF file, 1.4 MB. Copyright © 2020 Cao et al. 2020 Cao et al. This content is distributed under the terms of the Creative Commons Attribution 4.0 International license.

10.1128/mSystems.00853-19.5 TABLE S3 Upregulated proteins of L. plantarum 400g relative to L. plantarum P-8 grown in the presence of the ampicillin. Download Table S3, DOCX file, 0.04 MB. Copyright © 2020 Cao et al. 2020 Cao et al. This content is distributed under the terms of the Creative Commons Attribution 4.0 International license.

10.1128/mSystems.00853-19.6 TABLE S4 Downregulated proteins of L. plantarum 400g relative to L. plantarum P-8 grown in the presence of the ampicillin. Download Table S4, DOCX file, 0.05 MB. Copyright © 2020 Cao et al. 2020 Cao et al. This content is distributed under the terms of the Creative Commons Attribution 4.0 International license.

10.1128/mSystems.00853-19.7 TABLE S5 Upregulated proteins of L. plantarum 1600g relative to L. plantarum P-8 grown in the presence of the ampicillin. Download Table S5, DOCX file, 0.04 MB. Copyright © 2020 Cao et al. 2020 Cao et al. This content is distributed under the terms of the Creative Commons Attribution 4.0 International license.

10.1128/mSystems.00853-19.8 TABLE S6 Downregulated proteins of L. plantarum 1600g relative to L. plantarum P-8 grown in the presence of the ampicillin. Download Table S6, DOCX file, 0.04 MB. Copyright © 2020 Cao et al. 2020 Cao et al. This content is distributed under the terms of the Creative Commons Attribution 4.0 International license.

**FIG 6:**
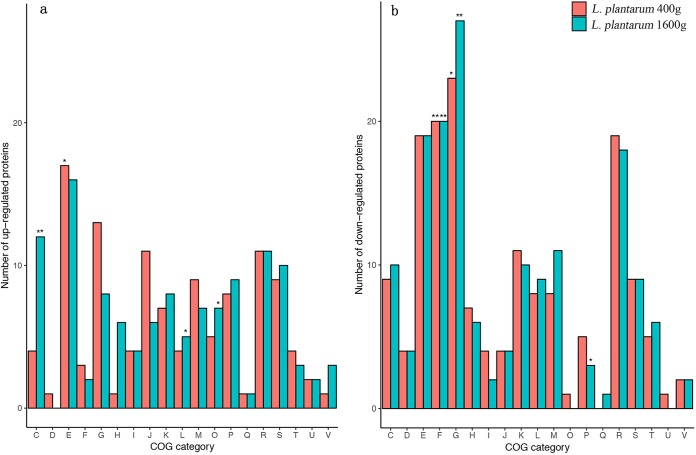
Functional distribution of differentially expressed proteins of ampicillin-adapted strains. Data represent upregulated (a) and downregulated (b) proteins of L. plantarum 400g and L. plantarum 1600g. Enrichment analyses were performed based on the protein expression of each COG functional category of L. plantarum 400g and L. plantarum 1600g relative to L. plantarum parental strain P-8. Significant enhancement is indicated by single and double asterisks (* represents *P* values of <0.05; ** represents *P* values of <0.01; Fisher’s exact test). COG functional categories are indicated as follows: C, energy production and conversion; D, cell cycle control, cell division, chromosome partitioning; E, amino acid transport and metabolism; F, nucleotide transport and metabolism; G, carbohydrate transport and metabolism; H, coenzyme transport and metabolism; I, lipid transport and metabolism; J, translation, ribosomal structure, and biogenesis; K, transcription; L, replication, recombination, and repair; M, cell wall/membrane/envelope biogenesis; O, posttranslational modification, protein turnover, chaperones; P, inorganic ion transport and metabolism; Q, secondary metabolites biosynthesis, transport, and catabolism; R, general function prediction only; S, function unknown; T, signal transduction mechanisms; V, defense mechanisms.

Most of the identified differentially expressed proteins could be assigned to a Clusters of Orthologous Group (COG) functional category (Fig. 6). Then, Fisher’s exact test was used for identifying the enriched COG functional categories in the ampicillin-adapted strains relative to the parental strain. Among the upregulated proteins (Fig. 6a), only one COG functional category was significantly enriched in L. plantarum 400g (i.e., amino acid transport and metabolism [COG functional category E], *P < *0.05), whereas three COG functional categories were significantly enriched in L. plantarum 1600g (i.e., energy production and conversion [C], *P < *0.01; replication, recombination and repair [L] and posttranslational modification, protein turnover, and chaperones [O], *P < *0.05). Among the downregulated proteins (Fig. 6b), the COG functional categories of carbohydrate transport and metabolism [G] and nucleotide transport and metabolism [F] were significantly enriched in both L. plantarum 400g (*P < *0.05 and *P < *0.01 for categories G and F, respectively) and L. plantarum 1600g (*P < *0.01 for both functional categories). The COG functional category inorganic ion transport and metabolism [P] was uniquely enriched in L. plantarum 1600g (*P < *0.05).

### Validation of protein expression by parallel reaction monitoring (PRM).

To validate the protein expression data obtained by TMT labeling analysis, we used PRM for verification of the expression of 10 differentially upregulated proteins of interest (5 from L. plantarum 400g and 5 from L. plantarum 1600g). An insignificantly modulated small heat shock protein (sHSP) (LBP_cg0109; proteomic ratio = 1.31, *P = *0.075) from L. plantarum 1600g was also included for verification due to its strong association with stress responses. For each chosen protein, between one and three unique peptides were selected for downstream quantification (Table S7).

10.1128/mSystems.00853-19.9 TABLE S7 Signature peptides used for PRM validation of the upregulated proteins in L. plantarum 400g and L. plantarum 1600g. Download Table S7, DOCX file, 0.02 MB. Copyright © 2020 Cao et al. 2020 Cao et al. This content is distributed under the terms of the Creative Commons Attribution 4.0 International license.

**FIG 7:**
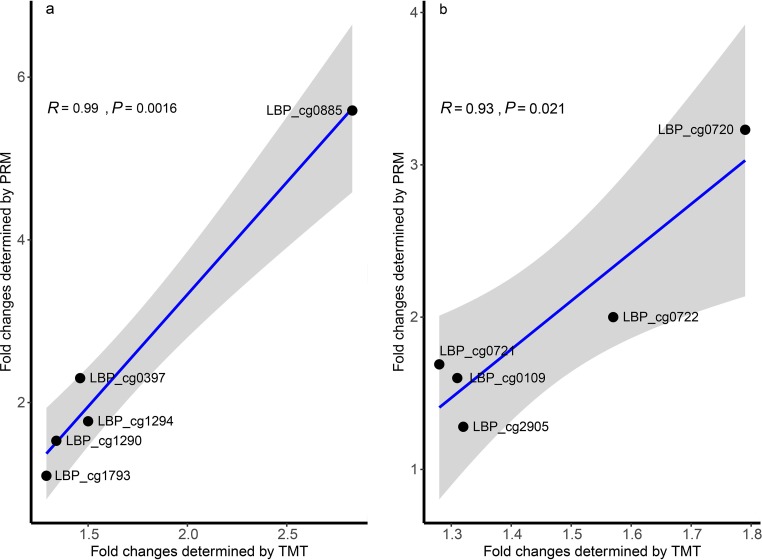
Correlation between expression patterns of 10 selected proteins detected by TMT and PRM analyses. (a) L. plantarum 400g. (b) L. plantarum 1600g. For both the TMT and PRM analyses, peptides from three biological replicates of the protein samples were analyzed. The strength and statistical significance of Pearson’s correlations between fold changes found by TMT and PRM analyses are represented by the *R* and *P* values, respectively. Confidence intervals are illustrated by the gray area. LBP_cg0397, l-serine dehydratase, beta subunit; LBP_cg0885, malate dehydrogenase; LBP_cg1290, enoyl-(acyl carrier protein) reductase; LBP_cg1294, acyl-CoA thioester hydrolase; LBP_cg1793, penicillin binding protein 2B; LBP_cg0109, sHSP; LBP_cg0720, hypothetical protein; LBP_cg0721, alkaline shock protein; LBP_cg0722, alkaline shock protein; LBP_cg2905, ClpL.

The expression patterns of the 10 target proteins detected by TMT labeling and PRM were largely consistent. Strong positive correlations were found between the fold changes of TMT and PRM for both L. plantarum 400g (*R *= 0.99, *P = *0.0016; Fig. 7a) and L. plantarum 1600g (*R *= 0.93, *P = *0.021; Fig. 7b) as determined by Pearson correlation analysis, confirming the accuracy of the proteomic data. The discrepancy in the fold changes of proteins found by the two methods could be explained by the high measurement precision of PRM as observed in other studies (19).

### Phenotypic analysis of the mutants.

**FIG 8:**
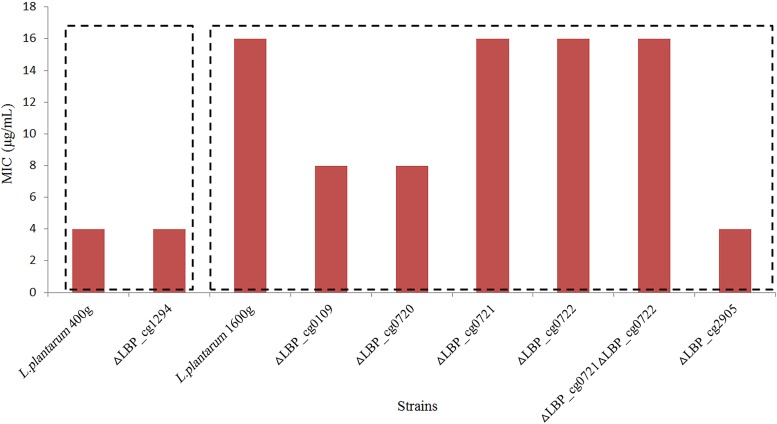
Ampicillin MICs for L. plantarum 400g and L. plantarum 1600g and their mutants. Each assay was repeated three times, and identical results were obtained in all cases.

The roles of the highly expressed proteins in ampicillin adaptation were further determined by constructing gene disruption mutants. Six target genes were selected for creating gene-inactivated mutants based on the results of PRM analysis. Changes in the ampicillin MICs determined for the mutants were analyzed (Fig. 8).

Our results showed that three of these genes, including genes encoding a sHSP (LBP_cg0109), an ATP-dependent Clp protease/ATP-binding subunit ClpL (LBP_cg2905), and a hypothetical protein (LBP_cg0720), were potentially associated with the ampicillin-resistant phenotype of the adapted strain L. plantarum 1600g (Fig. 8). The ampicillin MIC for the △LBP_cg2905 mutant (4 μg/ml) was only one-fourth of that for L. plantarum 1600g (16 μg/ml), while the ampicillin MIC for the △LBP_cg0109 and △LBP_cg0720 mutants was reduced by half (8 μg/ml). In contrast, inactivating LBP_cg1294 led only to poor cell growth and did not alter the ampicillin MIC of this mutant (Fig. 8). Finally, in comparison with the L. plantarum 1600g adapted strain, no phenotypic changes were observed in the three remaining mutants, i.e., the △LBP_cg0721, △LBP_cg0722, and △LBP_cg0721 △LBP_cg0722 mutants (Fig. 8). It seemed that the functional redundancy of the genes coding for alkaline shock proteins had no relationship with the ampicillin-resistant phenotype of the adapted strains.

**FIG 9:**
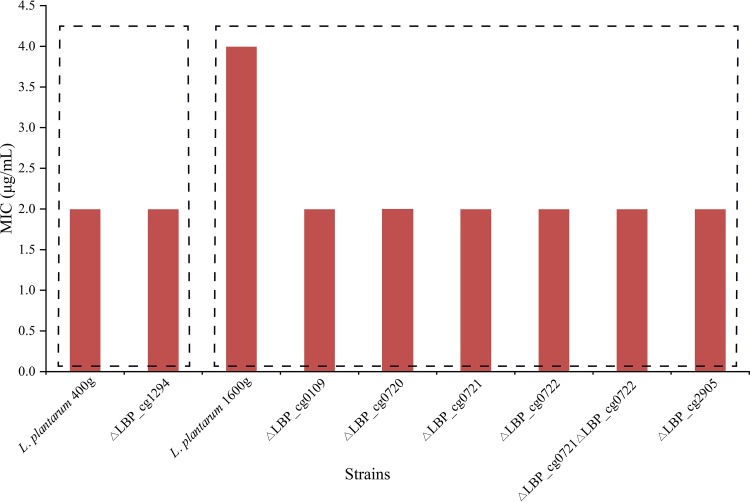
Amoxicillin MICs for L. plantarum 400g and L. plantarum 1600g and their mutants. Each assay was repeated three times, and identical results were obtained in all cases.

To test whether there was cross-resistance to other antibiotics, the MICs for amoxicillin of wild-type strains and mutants were also analyzed (Fig. 9). The amoxicillin MIC for the △LBP_cg1294 mutant remained identical to that of its wild type, L. plantarum 400g (Fig. 9). Interestingly, the amoxicillin MIC for all six mutants derived from L. plantarum 1600g (MIC, 4 μg/ml) was found to have been reduced by half to 2 μg/ml (Fig. 9).

## DISCUSSION

Ampicillin was the first broad-spectrum penicillin and belongs to the group of beta-lactam antibiotics. To kill bacteria, ampicillin acts as an irreversible inhibitor of the enzyme transpeptidase, which is needed for assembling the cell wall (20). Therefore, one common mechanism of ampicillin resistance operates via modulating bacterial transpeptidases like PBPs (21). Interestingly, the two nonsynonymous SNPs identified in L. plantarum 400g and L. plantarum 1600g were consistently located within genes encoding the PBPs. Moreover, the expression levels of some of these proteins were altered. Therefore, the modification of this group of protein at the genetic and protein levels could represent molecular signatures of long-term adaptation of L. plantarum to ampicillin. The levels of expression of PBP1A (LBP_cg1351) and PBP2A (LBP_cg1079) encoded by L. plantarum 400g and L. plantarum 1600g were suppressed. These proteins are bifunctional enzymes that polymerize and cross-link glycan chains through peptide bonds. Such catalyzation reactions occur in the final stages of bacterial peptidoglycan synthesis (22). Furthermore, the expression of PBP2B (LBP_cg1793) was slightly elevated in L. plantarum 1600g. This protein participates in peripheral elongation and septum synthesis and positioning (23).

The bacterial cell membrane acts like a barrier against external chemical stressors such as ampicillin. Some antibiotics inhibit bacterial growth by blocking fatty acid (FA) biosynthesis (24). Here, we observed elevated expression of some key proteins encoded by the FA biosynthetic operon, including an enoyl-(acyl carrier protein) reductase (LBP_cg1290) and an acyl-coenzyme A (CoA) thioester hydrolase (LBP_cg1294) in L. plantarum 400g. In the process of FA biosynthesis, enoyl-(acyl carrier protein) reductase catalyzes NADH-dependent reduction of crotonyl-CoA to butyryl CoA, while acyl-CoA thioester hydrolase catalyzes the hydrolysis of acyl-CoAs to free FA and CoA (25). Consistently, the expression of the biotin carboxyl carrier protein (LBP_cg0319) also increased even though it was not located within the FA biosynthetic operon. This protein is part of the acetyl-CoA carboxylase complex, which catalyzes the first and the rate-limiting step of FA synthesis (26). The disruption of acyl-CoA thioester hydrolase (LBP_cg1294) only retarded the growth of the mutant without changing the ampicillin-resistant phenotype, suggesting that ampicillin-mediated modulation of FA synthesis occurred in the bacteria under conditions of antibiotic stress; however, such a physiological change likely represented a membrane adaptation to the drug rather than a major mechanism that conferred the resistance.

Other forms of cell surface-associated adaptation induced by long-term ampicillin stress included increases in the expression of two alkaline shock proteins (LBP_cg0721 and LBP_cg0722) and of a hypothetical protein (LBP_cg0720). Genes coding these proteins clustered in the genome of L. plantarum P-8. An identical gene cluster existed in Staphylococcus aureus, and it encodes proteins relating to cell wall stress and cell wall homeostasis (27). Our work generated gene-inactivated mutants for all three genes; however, only the mutant △LBP_0720 showed attenuated ampicillin resistance, suggesting a role of this hypothetical protein in protecting the bacteria from the antibiotic stress. However, limited information is available regarding its biological function except that it contains a DUF2273 domain, and the close proximity of LBP_0720 to the alkaline shock proteins could imply that their cellular functions are closely linked (27).

To promote survival during antibiotic challenge, ampicillin-adapted L. plantarum P-8 seemed to enhance the machinery of protein homeostasis, which is associated with the sHSPs. They act as cellular gatekeepers to prevent premature protein folding by assisting proper protein folding. A sHSP (LBP_cg0109) showed elevated expression in L. plantarum 1600g; sHSPs function together with the HSP70 machinery to maintain cellular proteins in a state competent for refolding or clearance (28). In lactic acid bacteria (LAB), sHSPs are associated with several types of stress responses. For example, the sHSPs in Lactococcus lactis improve the stress tolerance of bacteria under heat and acid conditions (29); the expression of sHSP in L. sanfranciscensis protects the bacteria from potential cell damage caused by oxidative stress (30). The deletion of the LBP_cg0109 gene in L. plantarum 1600g significantly reversed its antibiotic-resistant phenotype, indicating an important role of this protein in ampicillin adaptation in L. plantarum P-8.

Some bacterial proteases serve as partners of sHSPs to maintain protein homeostasis by degrading proteins that are folded incorrectly (31). The most extensively studied sHSP is the Clp protease complex in Escherichia coli, which consists of a proteolytic subunit (ClpP) together with other subunits, namely, ClpA, ClpC, and ClpX (32). The substrate specificity of ClpP is often determined by the regulatory ClpP subunits, and the ClpP subunits alone can function as chaperones. The Clp proteases are found also in several LAB species, and they are important for the survival of some LAB species, including L. casei (33), L. delbrueckii (34), and L. reuteri (35), in acid stress and bile salts. Furthermore, Clp proteases are associated with thermotolerance of the species Streptococcus thermophilus (36, 37). The inactivation of a highly expressed ATP-dependent Clp protease, ATP-binding subunit ClpL (LBP_cg2905), in L. plantarum 1600g resulted in apparent reversal of the ampicillin-resistant phenotype, suggesting that this protein plays a role in protecting the bacterial cells from external ampicillin stress. To our knowledge, this is the first report demonstrating the role of sHSPs in adaptation to antibiotics in LAB.

Some proteins relating to carbon utilization were abundantly expressed either in strain L. plantarum 400g or in strain L. plantarum 1600g. Among them, only a few glycolytic proteins were identified, even though glucose was the primary carbon source in the culture medium. Two glycolytic pathway-associated proteins (namely, glyceraldehyde-3-phosphate dehydrogenase [LBP_cg1476] and pyruvate kinase [LBP_cg0590]) were abundantly expressed, which might have enhanced pyruvate production. Consistently, an enzyme involving in downstream pyruvate metabolism, a malate dehydrogenase (LBP_cg0885) (38), showed significantly stronger expression than was seen with the parental strain. In contrast, the expression levels of several proteins that are linked to lactose and galactose uptake and utilization (LBP_cg2828), fructose utilization (LBP_cg1704 and LBP_cg2868), mannitol utilization (LBP_cg0206), sorbitol utilization (LBP_cg2912, LBP_cg2913, and LBP_cg2917), citrate utilization (LBP_cg0871, LBP_cg0872, LBP_cg0876 to LBP_cg0878, and LBP_cg0880), and transport of another sugar (LBP_cg2833) also increased, indicating that L. plantarum P-8 might have shifted its carbon source metabolism during ampicillin adaptation. Such physiological and biochemical modulation might help maximize the extraction of cellular energy when lactobacilli are exposed to environmental stressors (39).

Amino acids represent another group of essential nutrients required for bacterial growth. Our proteomic analysis of L. plantarum 400g and L. plantarum 1600g revealed intensified expression of transport systems of peptides (LBP_cg0964, LBP_cg0965, LBP_cg0967, and LBP_cg0585) and amino acids (LBP_cg2627 and LBP_cg2613). These transport systems translocate amino acids or peptides to the cytoplasm (39). In addition, there was increased expression in the group of proteins responsible for serine utilization, including a seryl-tRNA synthetase 1 (LBP_cg0395) and a serine transporter (LBP_cg0396) as well as the alpha (LBP_cg0398) and beta (LBP_cg0397) subunits of an l-serine dehydratase. Of particular interest is the l-serine dehydratase, which catalyzes the cleavage of l-serine into equimolar amounts of pyruvate and ammonia (40). The strengthening of expression of these proteins might suggest that increased cellular levels of serine and, subsequently, pyruvate were necessary for the adaptive L. plantarum P-8 strains to cope with the antibiotic stress.

In addition to our analysis of the modulation of bacterial physiology at the protein level, we identified some antibiotic resistance genes that potentially contributed to the ampicillin-adapted phenotype. However, a detailed analysis of the genome of L. plantarum P-8 revealed that none of these affected genes were flanked by any mobile genetic elements, indicating their high genomic stability within the bacterial chromosome and a low risk of lateral transfer to other bacteria. Therefore, the spread of antibiotic resistance genes is unlikely to be a significant safety concern.

Finally, six (of a total of seven) gene inactivation mutants generated in this work became less resistant to amoxicillin. Disruption of any member of the gene cluster LBP_cg0720 to LBP_cg0722 resulted in attenuated amoxicillin resistance, differing from the phenotypic responses of mutants toward ampicillin. Such results suggest that the mechanisms of resistance to ampicillin and amoxicillin overlap at least partially, though the details cannot be entirely deciphered by analysis of the current proteomic data.

### Conclusion.

The current work applied an integrative genomic and proteomic analysis to elucidate the adaptive mechanism of L. plantarum P-8 in coping with ampicillin-induced stress, and selected targeted genes were disrupted to evaluate their role in the adaptive evolution. Our results suggested that some stress-related proteins, such as ClpL and a small heat shock protein, possibly contributed to the antibiotic-resistant phenotype. Although none of the genes identified as possibly involved in the ampicillin adaptation were located next to any mobile genetic element, suggesting a low risk of lateral gene transfer, our study did raise concerns about the biosafety or biosecurity of probiotics, considering their enhanced antibiotic resistance after prolonged exposure to the drug. It is thus worth exploring further if the current observations are strain specific or universal and applicable to other probiotics.

## MATERIALS AND METHODS

### Experimental design and statistical rationale.

The ALE experiment was performed with three bacterial lines to determine the evolutionary behavior of the probiotic bacterium L. plantarum P-8 under conditions of ampicillin selection pressure. The evolutionary behavior was evaluated based on changes in bacterial fitness of survival in antibiotics-containing medium, as represented by MICs. Descendant bacteria representing different evolutionary stages were subjected to integrative genomic and proteomic analysis to identify genes/proteins that were important for the adaptive phenotype of the evolved bacterial lines. Quantitative proteomics analysis was performed using a high-resolution mass spectrometer. To ensure the reliability of the proteomic analysis, samples of the selected adapted strains were prepared from the three biological replicates. Significant differences were identified by *t* test with a *P* value of <0.05. After comparative proteomic analysis, PRM was used to validate the expression of proteins of interest. Then, target gene disruption was performed to determine the biological role of selected target genes of interest.

### Bacterial strains and cultivation.

Lactobacillus plantarum P-8 was obtained from the Key Laboratory of Dairy Biotechnology and Engineering, Ministry of Education, Inner Mongolia Agricultural University. It was cultivated either in de Mann-Rogosa-Sharpe (MRS) broth (catalog no. CM0359, Oxoid) or in LAB susceptibility test medium (LSM) (41, 42). The LSM consisted of 90% Iso-Sensitest medium (catalog no. CM0473; Oxoid) and 10% MRS broth. The E. coli DH5α strain was used for standard cloning, and the culture was grown aerobically in Luria-Bertani broth at 37°C. The antibiotics chloramphenicol (10 μg/ml for both E. coli and L. plantarum P-8) and erythromycin (250 μg/ml and 5 μg/ml for E. coli and L. plantarum P-8, respectively) were used for selecting genetic mutants.

### ALE experiment.

The ALE experiment was performed according to methods described previously by Pena-Miller et al. (43). Briefly, a frozen stock of L. plantarum P-8 was activated by overnight cultivation in MRS broth. Then, the bacteria were allowed to grow at 37°C for 72 h on MRS agar plates. Three colonies were randomly picked and were separately inoculated into three tubes of 5 ml LSM supplemented with ampicillin (0.25 μg/ml) or without any antibiotics. The ampicillin concentration represented half the strength of the MIC of L. plantarum P-8. Each of these cultures was maintained as an individual long-term cell line by daily inoculation of 50 μl of the original culture (1% [vol/vol]) into the respective fresh growth media. A 100-fold daily increase in bacterial growth would be roughly equal to 6.6 generations for each subcultivation. The cells were sampled regularly, and they were coded according to their growth generation in consecutive order. Phenotypic changes (as measured by the ampicillin MICs) of each bacterial line were recorded every 200 generations. Frozen stocks were made regularly throughout the ALE experiment.

### Fitness evaluation represented by MICs.

Phenotypic changes of bacterial cultures were measured by analysis of the MIC for ampicillin, which was determined using the broth dilution method described in the Clinical and Laboratory Standards Institute (CLSI) and International Diabetes Federation (IDF) guidelines (44, 45). Briefly, the bacterial cell density was adjusted to the turbidity level of McFarland standard of 1 (∼3 × 10^8^ CFU/ml). They were then diluted 500-fold (∼6 × 10^5^ CFU/ml). Doubling dilutions of antibiotics, ranging from 0.032 to 16 μg/ml for ampicillin, were freshly prepared in LSM broth. All of the antibiotics-containing or control tubes were then inoculated with equal volumes of the respective diluted bacterial suspensions (final bacterial concentration of 3 × 10^5^ CFU/ml). The MIC endpoints were read after 48 h of incubation at 37°C. The quality control strain, L. plantarum ATCC 334, was used for controlling the precision and accuracy of the susceptibility test. Each assay was repeated three times.

### Genomic resequencing and identification of SNPs.

To study the mechanism underlying the adaptive evolution process, genomic resequencing was performed on selected adapted strains. Genomic DNA isolated from strains was sequenced by the Shanghai Majorbio Bio-Pharm Technology Corporation using an Illumina HiSeq 4000 platform. Raw reads of 150 bp in length with an insertion size of 400 to 500 bp were generated. The average coverage depth of high-quality data was over 100-fold for each sample. To identify SNPs, the raw sequence reads were imported into CLC Bio Genomics Workbench V8.5.1 (CLC Inc., Aarhus, Denmark) and mapped against the L. plantarum P-8 genome with 80% identity and a length fraction setting of 0.5. The variant detector available in the software, namely, Fixed Ploidy Variant Detection, was used to call mutations. The parameters of the detector were single nucleotide variation coverage of >20, variant probability of 90%, and ploidy of 1. Synonymous or nonsynonymous sites were discriminated by using the protocol of Amino Acid Changes available in the Functional Consequences module. In addition, the upstream and downstream sequences of each SNP in the final set were retrieved, and primers specific to each identified sequence were designed for PCR amplification (see Table S8 in the supplemental material). The PCR products were subjected to Sanger sequencing to verify the identified SNPs.

10.1128/mSystems.00853-19.10 TABLE S8 Primers used for validation of SNPs. Download Table S8, DOCX file, 0.02 MB. Copyright © 2020 Cao et al. 2020 Cao et al. This content is distributed under the terms of the Creative Commons Attribution 4.0 International license.

### Preparation and TMT labeling of protein extracts for proteomic analysis.

Strains representing different stages of adaptation to antibiotics were selected. To ensure the reliability of the proteomic analysis, three samples were prepared from three biological replicates. Protein extracts were prepared in triplicate by lysing the collected cells in SDT lysis buffer (Invitrogen, Carlsbad, USA) (4% SDS, 100 mM dithiothreitol [DTT], 150 mM Tris-HCl [pH 8.0]). Then, the lysates were homogenized by the use of a homogenizer (6.0 m/s, 60 s, performed twice), sonicated, and boiled for 15 min. Boiled lysates were centrifuged at 14,000 × *g* for 15 min at room temperature, and the supernatants were collected. The protein concentration of the supernatants was assayed with a Pierce bicinchoninic acid (BCA) protein assay kit (Bio-Rad, USA).

For each sample, 200 μg of protein was mixed with 30 μl SDT buffer and denatured in 8 M urea buffer (dissolved in 150 mM Tris-HCl, pH 8.0). The low-molecular-weight components in the denatured protein mix were removed by repeated ultrafiltration (Microcon units; 10 kDa). Then, the filtrates were incubated in 100 μl iodoacetamide (IAA; 100 mM in urea buffer) for 30 min in dark to block reduced cysteine residues. Finally, the protein suspensions were digested with 4 μg trypsin (Promega)–40 μl triethylammonium bicarbonate (TEAB) buffer overnight at 37°C.

For each sample, the peptides were collected after centrifugation through the 10-kDa filter. A 100-μm volume of the peptide mixture was labeled using TMT reagent following the instructions of the manufacturer (Thermo Fisher Scientific). The TMT-labeled digests were further fractionated into 10 fractions by using a high-pH reversed-phase fractionation kit (Thermo Fisher Scientific) according to the manufacturer’s instructions.

### Proteomic analysis by liquid chromatography (LC)-tandem mass spectrometry (MS/MS).

Proteomic analyses were performed on an Easy-nLC system coupled to a Q-Exactive mass spectrometer (Thermo Scientific) for 60 min. For LC analysis, the peptide mixture was loaded onto a reversed-phase trap column (Thermo Scientific Acclaim PepMap100) (100 μm by 2 cm) (nanoViper C_18_) connected to a C_18_ reversed-phase analytical column (Thermo Scientific Easy column) (10 cm long, 75-μm inner diameter, 3-μm resin particle size) in buffer A (0.1% formic acid) and separated with a linear gradient of buffer B (84% acetonitrile and 0.1% formic acid) at a flow rate of 300 nl/min controlled by IntelliFlow technology.

The mass spectrometer was operated in positive-ion mode. The generated MS data (.raw file) were acquired using a data-dependent method, which selected the most abundant precursor ions from the survey scan (300 to 1,800 *m*/*z*) dynamically for higher-energy C-trap dissociation (HCD) fragmentation. The automatic gain control (AGC) target was set to 3e−6, with a maximum inject time of 10 ms. The dynamic exclusion duration was set to 40.0 s. Survey scans were acquired at a resolution of 70,000 at *m*/*z* 200, and the resolution for HCD spectra was set to 35,000 at *m*/*z* 200 (TMT 10plex). The isolation width was 2 *m*/*z*. The normalized collision energy level was 30 eV. The underfill ratio, which specified the minimum percentage of the target value likely to be reached at the maximum fill time, was defined as 0.1%. The instrument was run with the peptide recognition mode enabled.

### Data processing.

Raw data files were converted into Mascot Generic Format (MGF) files using Proteome Discoverer 1.4 (Thermo Fisher, Waltham, USA) (46). By using the Mascot engine (Matrix Science, London, United Kingdom; version 2.2), the detected proteins were identified and quantified. A false-discovery-rate (FDR) cutoff value of <0.01 was applied to ensure the credibility of the obtained results. An ion score cutoff value of >20 was used for peptide identification, and a minimum of 2 unique peptides were used for identifying each protein.

The MS data were also mapped with the reference protein sequences of L. plantarum P-8 (GenBank accession numbers CP005942.2 for chromosomes and CP005943.2 to CP005947.2, CP005948.1, and CP010527.1 for plasmids; 3,120 entries) using Mascot 2.2 (47, 48). The parameters were set as follows: (i) trypsin digestion with a maximum of two missed cleavages; (ii) fragment ion mass tolerance of 0.1 Da and precursor ion mass tolerance of 20.0 ppm; (iii) carbamidomethylation of cysteine and TMT 10plex of lysine and the N terminus as fixed modifications; (iv) oxidation of methionine and TMT 10plex of tyrosine as a variable modification. For quantitative analysis, the ion peak intensities of peptides in the current samples were recorded and normalized by the use of Proteome Discoverer 1.4. Proteins displaying a *P* value of <0.05 by *t* test analysis were considered statistically significant. A 1.2-fold change was used as the threshold for selection of regulated proteins. To understand their biological roles, the significant differentially expressed proteins were functionally assigned by the COGs of proteins and the Kyoto Encyclopedia of Genes and Genomes databases (49). The pattern of relative protein expression in different samples was analyzed by the use of Cluster3.0 (http://bonsai.hgc.jp/~mdehoon/software/cluster/software.htm). Data were presented in a heat map. Venn diagrams were constructed using Venny 1.0 (50).

### Validation of protein expression by PRM.

To ensure the reliability of the protein expression data obtained by the TMT labeling method, the levels of expression of selected proteins were validated under the tier 2 level by PRM (19). Briefly, peptides from three biological replicates of the protein samples were produced as described for the TMT labeling, followed by spiking an internal reference standard, i.e., an AQUA stable isotope peptide. The tryptic peptides were desalted on C_18_ stage tips before proceeding to reversed-phase chromatography performed on an Easy nLC-1200 system (Thermo Scientific). A 1-h LC gradient ranging from 5% to 23% of acetonitrile in 42 min was applied. The PRM analysis was achieved on a Q-Exactive Plus mass spectrometer (Thermo Scientific). The MS conditions (including collision energy, charge state, and retention times) were optimized for each target protein experimentally using unique peptides having strong signal intensity and confidence. The MS was operated in positive-ion mode. The full MS1 scan was acquired at a resolution of 60,000 (at *m*/*z* 200), an AGC target value of 3.0 × 10^−6^, and a maximum ion injection time of 250 ms. Full MS scans were followed by 20 PRM scans at a resolution of 30,000 (at *m*/*z* 200) with an AGC target value of 3.0 × 10^−6^, and a maximum injection time of 200 ms. The target peptides were isolated with a 1.6-Th window. Ion activation and dissociation were performed at the normalized collision energy level of 27 in the HCD collision cell. Raw data were analyzed using Skyline (MacCoss Lab, University of Washington) (51) to report the differentially expressed proteins in each sample relative to the standard reference.

### Construction and analysis of gene disruption mutants.

Six genes, namely, LBP_cg0109, LBP_cg0720, LBP_cg0721, LBP_cg0722, LBP_cg1294, and LBP_cg2905, were selected for gene disruption. They putatively encoded a sHSP, two hypothetical proteins, two alkaline shock proteins, an acyl-CoA thioester hydrolase, and a ClpL, respectively. The plasmids and primers used for DNA cloning and generating target inactivation L. plantarum P-8 mutants are listed in Table 1.

**TABLE 1:** Strains, plasmids, and primers used in this study

Strain, plasmid, or primer	Description or primer sequence^*a*^	Reference or source
Strains		
E. coli DH5α	Cloning host	This study
L. plantarum P-8	Isolated from traditional fermented cow milk in Inner Mongolia, China	8
L. plantarum 400g	L. plantarum P-8 propagated in LSM broth containing ampicillin 0.25 μg/ml for 2 months	This study
L. plantarum 1600g	L. plantarum P-8 propagated in LSM broth containing ampicillin 0.25 μg/ml for 8 months	This study
L. plantarum 400g-1294::*lox66*-P32-*cat-lox71*	Derivative of 400g containing a *lox66*-P32-*cat-lox71* replacement of LBP_cg1294	This study
L. plantarum 400g-Δ1294	Derivative of 400g-1294::*lox66*-P32-*cat-lox71* containing a *lox72* replacement of LBP_cg1294	This study
L. plantarum 1600g-0109::*lox66*-P32-*cat-lox71*	Derivative of 1600g containing a *lox66*-P32-*cat-lox71* replacement of LBP_cg0109	This study
L. plantarum 1600g-Δ0109	Derivative of 1600g-0109::*lox66*-P32-*cat-lox71* containing a *lox72* replacement of LBP_cg0109	This study
L. plantarum 1600g-0720::*lox66*-P32-*cat-lox71*	Derivative of 1600g containing a *lox66*-P32-*cat-lox71* replacement of LBP_cg0720	This study
L. plantarum 1600g-Δ0720	Derivative of 1600g-0720::*lox66*-P32-*cat-lox71* containing a *lox72* replacement of LBP_cg0720	This study
L. plantarum 1600g-0721::*lox66*-P32-*cat-lox71*	Derivative of 1600g containing a *lox66*-P32-*cat-lox71* replacement of LBP_cg0721	This study
L. plantarum 1600g-Δ0721	Derivative of 1600g-0721::*lox66*-P32-*cat-lox71* containing a *lox72* replacement of LBP_cg0721	This study
L. plantarum 1600g-0722::*lox66*-P32-*cat-lox71*	Derivative of 1600g containing a *lox66*-P32-*cat-lox71* replacement of LBP_cg0722	This study
L. plantarum 1600g-Δ0722	Derivative of 1600g-0722::*lox66*-P32-*cat-lox71* containing a *lox72* replacement of LBP_cg0722	This study
L. plantarum 1600g-0722-0721::*lox66*-P32-*cat-lox71*	Derivative of 1600g containing a *lox66*-P32-*cat-lox71* replacement of LBP_cg0721 in the strain of 1600G-Δ0722	This study
L. plantarum 1600g-Δ0722-0721	Derivative of 1600g-0722-0721::*lox66*-P32-*cat-lox71* containing a *lox72* replacement of LBP_cg0721 in the strain of 1600G-Δ0722	This study
L. plantarum 1600g-2905::*lox66*-P32-*cat-lox71*	Derivative of 1600g containing a *lox66*-P32-*cat-lox71* replacement of LBP_cg2905	This study
L. plantarum 1600g-Δ2905	Derivative of 1600g-2905::*lox66*-P32-*cat-lox71* containing a *lox72* replacement of LBP_cg2905	This study
Plasmids		
pNZ5319	Cm^r^ Em^r^; containing *lox66*-P32-*cat-lox71* cassette for multiple gene replacement in Gram-positive bacteria	52
pNZ5319-0109Up-Down	Cm^r^ Em^r^; pNZ5319 derivative containing homologous regions up- and downstream of LBP_cg0109	This study
pNZ5319-0720Up-Down	Cm^r^ Em^r^; pNZ5319 derivative containing homologous regions up- and downstream of LBP_cg0720	This study
pNZ5319-0721Up-Down	Cm^r^ Em^r^; pNZ5319 derivative containing homologous regions up- and downstream of LBP_cg0721	This study
pNZ5319-0722Up-Down	Cm^r^ Em^r^; pNZ5319 derivative containing homologous regions up- and downstream of LBP_cg0722	This study
pNZ5319-0722-0721Up-Down	Cm^r^ Em^r^; pNZ5319 derivative containing homologous regions up- and downstream of LBP_cg0721 in the strain of 1600G-Δ0722	This study
pNZ5319-1294Up-Down	Cm^r^ Em^r^; pNZ5319 derivative containing homologous regions up- and downstream of LBP_cg1294	This study
pNZ5319-2905Up-Down	Cm^r^ Em^r^; pNZ5319 derivative containing homologous regions up- and downstream of LBP_cg2905	This study
pMSPCre	Em^r^; expression of *cre*	53
Primers		
0109upF	5′-CCGCTCGAGTATTCGGGTGTCAGGATAAT-3′	This study
0109upR	5′-AGCTTTGTTTAAACTAAAGTATTAGCCATACTAACAATC-3′	This study
0109downF	5′-TCCCCCCGGGATCGAAATTCAATAATTAAATCATT-3′	This study
0109downR	5′-GGGTTTGAGCTCCCCGTTTAGTTGACCAAGTAG-3′	This study
0720upF	5′-CCGCTCGAGTGTTCAGATTTTAAGTGACAATT-3′	This study
0720upR	5′-AGCTTTGTTTAAACCTGTGCGTTACTCATCAGGA-3′	This study
0720downF	5′-TCCCCCCGGGATTACAATGGCACAACCAGAACAAC-3′	This study
0720downR	5′-GGGTTTGAGCTCATGCTGTTCATCCGTAAT-3′	This study
0721upF	5′-CCGCTCGAGTTTAGTTGGTGCCTGGAT-3′	This study
0721upR	5′-AGCTTTGTTTAAACTTCTGGTTGTGCCATTGTA-3′	This study
0721downF	5′-TCCCCCCGGGCGGCAAGTTGAATAGCACG-3′	This study
0721downR	5′-GGGTTTGAGCTC ACCTTCGGCAGCGTAATG-3′	This study
0722upF	5′-CCGCTCGAGAGGTTGGCCAGTTAACGATTC-3′	This study
0722upR	5′-AGCTTTGTTTAAACCGCTGTTGCTTCCATCAAAAT-3′	This study
0722downF	5′-TCCCCCCGGGGGCCCACATTAATGGCAG-3′	This study
0722downR	5′-GGGTTTGAGCTCGATATTTAACGTCCCTAAATGTGTA-3′	This study
0722-0721upF	5′-AGGACCGATAACGCGCTCGAGTTTAGTTGGTGCCTGGAT-3′	This study
0722-0721upR	5′-CGGTAGATTTAAATTGTTTAAACTTCTGGTTGTGCCATTGTA-3′	This study
0722-0721downF	5′-ATACGAACGGTACAGCCCGGGCGGCAAGTTGAATAGCAC-3′	This study
0722-0721downR	5′-TTCTCGTAGCGATCGGAGCTCTCTGACCGCACAGTTGAT-3′	This study
1294upF	5′-AGGACCGATAACGCGCTCGAGAGTCACGTTAACACGGGTTG-3′	This study
1294upR	5′-CGGTAGATTTAAATTGTTTAAACATGAATTGGTTCCATGTCTG-3′	This study
1294downF	5′-ATACGAACGGTACAGCCCGGGGAATTGCCGTGGTGAATC-3′	This study
1294downR	5′-TTCTCGTAGCGATCGGAGCTCAGGCCACCGTTTGACC-3′	This study
2905upF	5′-CCGCTCGAGGCATACAGACGGATAAGA-3′	This study
2905upR	5′-CTATTTAAATCTTAAATCAGTGTTCATAAATAACT-3′	This study
2905downF	5′-TCCCCCCGGGTGCCATATTACAACACCTCCATAAA-3′	This study
2905downR	5′-GGGTTTGAGCTCCACTTTAGCGTTGGTTTT-3′	This study
85	5′-GTTTTTTTCTAGTCCAAGCTCACA-3′	52
87	5′-GCCGACTGTACTTTCGGATCCT-3′	52
CatF	5′-TCAAATACAGCTTTTAGAACTGG-3′	52
CatR	5′-ACCATCAAAAATTGTATAAAGTGGC-3′	52
EryintF	5′-CGATACCGTTTACGAAATTGG-3′	52
EryintR	5′-CTTGCTCATAAGTAACGGTAC-3′	52
CreF	5′-CTAACTCGAGTGATCACCAATTC-3′	52
CreR	5′-GGCTATCAATCAAAGCAACACG-3′	52

a The restriction sites in the primer sequences are underlined. Cm^r^, chloramphenicol resistance; Em^r^, erythromycin resistance.

The target disruption mutants were made according to previously described methods (52). Briefly, the upstream and downstream flanking sequences of the target genes were amplified by PCR using the respective gene-specific primers. The sizes of the amplified fragments were verified before cloning into the XhoI or PmeI (SwaI) and Ecol53KI or XmaI restriction sites of suicide vector pNZ5319 by the use of a ClonExpress II one-step cloning kit (Vazyme, USA) or conventional methods. The respective mutagenesis vectors were then electroporated into the ampicillin-adapted strain. Chloramphenicol-resistant and erythromycin-sensitive transformants were selected for confirmation of correct double-crossover event. The PCR checking was performed with primer pairs specific to the respective target gene and the *cat* selectable marker in each case. The P32-cat selectable marker cassette was cut out by introducing the *cre* expression plasmid pMSPCre into the gene-inactivated mutants. Successful excision of the P32-cat cassette would render the mutants erythromycin resistant and chloramphenicol sensitive. The *cre*-mediated excision of the antibiotic selection cassette was confirmed again by PCR using primers spanning the recombination locus. The pMSPCre vector was cured by maintaining the mutants in culture medium without erythromycin (53). Additionally, the PCR products were checked by sequencing when necessary. The MICs of antibiotics were compared for the wild-type strain and the gene-inactivated mutants.

### Data availability.

The MS proteomics and PRM data have been deposited to the ProteomeXchange Consortium via the PRIDE partner repository (http://www.ebi.ac.uk/pride) with data set identifiers PXD010054, PXD011623, and PXD011624, respectively. The genomes of two adapted strains have been deposited in the National Center for Biotechnology Information (NCBI) Sequence Read Archive (SRA) (http://trace.ncbi.nlm.nih.gov/Traces/sra/sra.cgi) under accession number PRJNA507290.
