# Supplementary figures and images for: Adaptation of Lactobacillus plantarum to Ampicillin Involves Mechanisms That Maintain Protein Homeostasis
Source: mSystems. 2020 Jan 28;5(1):e00853-19. doi: 10.1128/mSystems.00853-19 (PMC6989132; doi:10.1128/mSystems.00853-19)

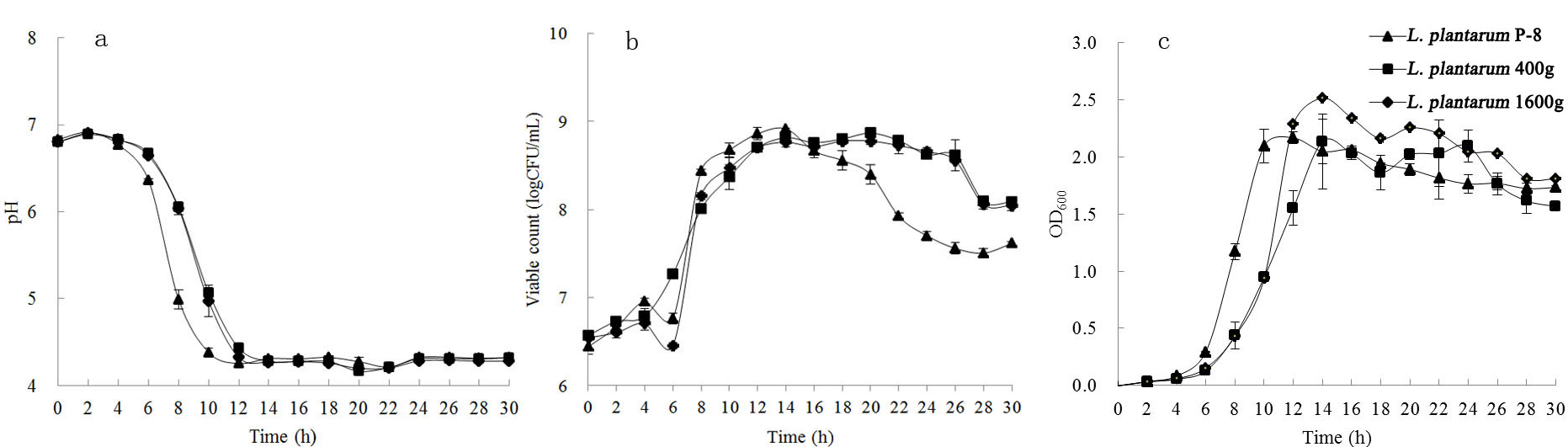

Supplement: FIG S1 [file mSystems.00853-19-sf001.tif]

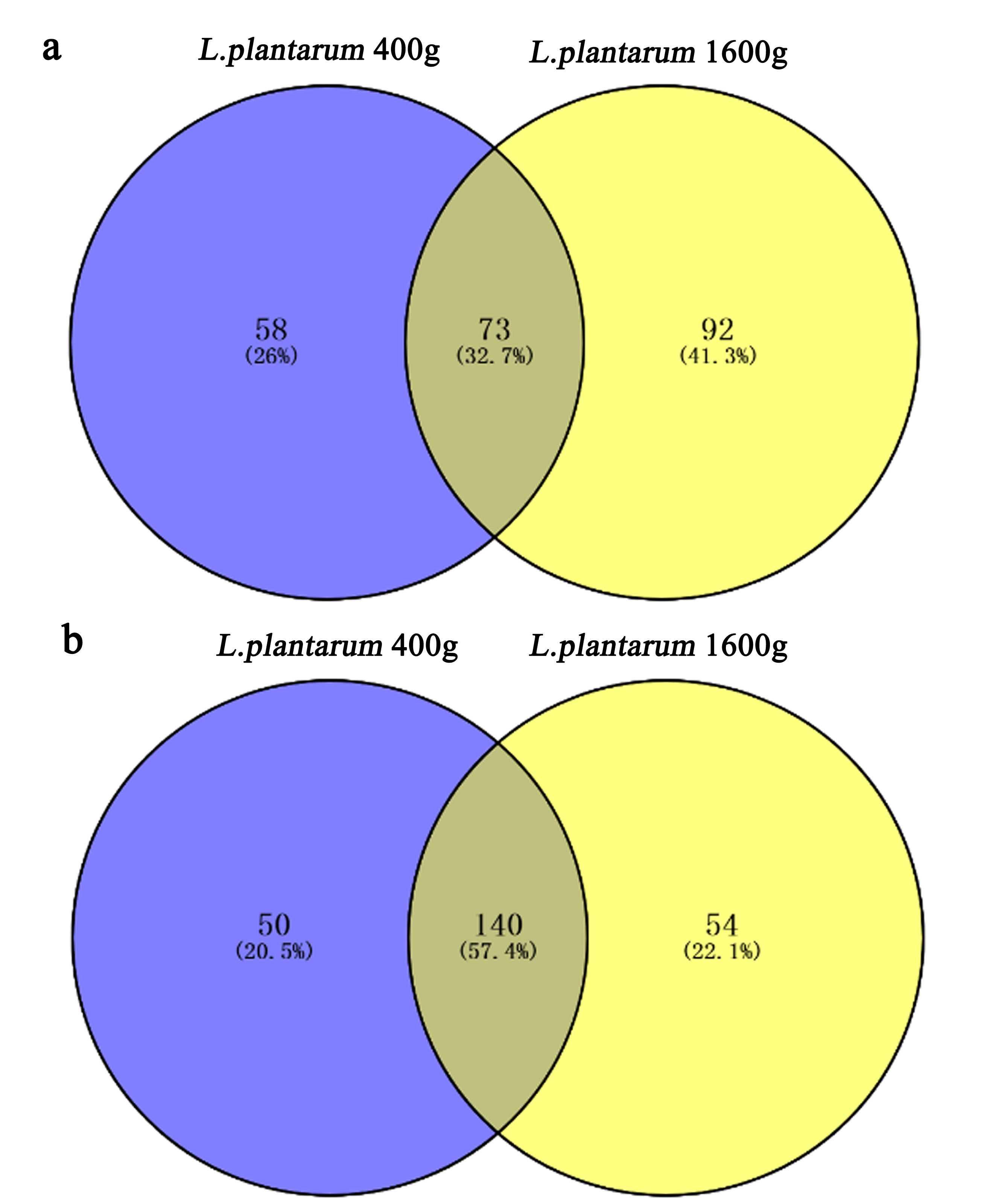

Supplement: FIG S2 [file mSystems.00853-19-sf002.tif]
