# Supplementary material for: Adaptation of Lactobacillus plantarum to Ampicillin Involves Mechanisms That Maintain Protein Homeostasis
Source: mSystems. 2020 Jan 28;5(1):e00853-19. doi: 10.1128/mSystems.00853-19 (PMC6989132; doi:10.1128/mSystems.00853-19)
Supplement: TABLE S1 [file mSystems.00853-19-st001.docx]

**Table S1** General information of SNPs in *L. plantarum* 400g and *L. plantarum* 1600g in comparison with *L. plantarum* P-8

| **Genome Location** | ***L. plantarum* 400g^a^** | ***L. plantarum* 1600g^a^** | **Gene Locus** | **Base change** | **Type** | **Description** |
| --- | --- | --- | --- | --- | --- | --- |
| 668215 | × | × | - | G🡪C | intergenic | - |
| 1256376 | × | × | LBP_cg1189 | G🡪C | non-synonymous | Penicillin binding protein 2B |
| 1846178 | × | × | LBP_cg1793 | G🡪A | non-synonymous | Penicillin binding protein 2B |
| 1875677 | - | × | LBP_ct0056 | A🡪T | - | tRNA-Thr |
| 2367869 | - | × | LBP_cg2310 | C🡪A | non-synonymous | ABC transporter, permease protein |
| 2398461 | × | × | LBP_cg2340 | G🡪A | non-synonymous | ABC superfamily ATP binding cassette transporter, membrane protein |

^a^Indicates in which strain the SNPs were detected.
