# Supplementary material for: Adaptation of Lactobacillus plantarum to Ampicillin Involves Mechanisms That Maintain Protein Homeostasis
Source: mSystems. 2020 Jan 28;5(1):e00853-19. doi: 10.1128/mSystems.00853-19 (PMC6989132; doi:10.1128/mSystems.00853-19)
Supplement: TABLE S2 [file mSystems.00853-19-st002.docx]

**Table S2.** General information of identified proteins

| **Accession** | **Description** | **Unique Peptides** | **Peptide spectrum matches** |
| --- | --- | --- | --- |
| LBP_cg0805 | DNA-directed RNA polymerase subunit beta | 78 | 434 |
| LBP_cg0804 | DNA-directed RNA polymerase subunit beta | 54 | 276 |
| LBP_cg1867 | Alanine--tRNA ligase | 52 | 184 |
| LBP_cg2703 | Formate C-acetyltransferase | 51 | 705 |
| LBP_cg1904 | Valyl-tRNA synthetase | 51 | 193 |
| LBP_cg0802 | ATPase with chaperone activity, ATP-binding subunit | 47 | 255 |
| LBP_cg1742 | Pyruvate carboxylase | 47 | 141 |
| LBP_cg0809 | Elongation factor G | 45 | 855 |
| LBP_cg1586 | Chaperone protein dnaK | 43 | 681 |
| LBP_cg2175 | Phosphoketolase | 43 | 319 |
| LBP_cg1599 | Translation initiation factor IF-2 | 43 | 169 |
| LBP_cg2905 | ATP-dependent Clp protease, ATP-binding subunit ClpL | 42 | 300 |
| LBP_cg1481 | S14 family endopeptidase Clp | 41 | 230 |
| LBP_cg0547 | Protein translocase subunit secA | 41 | 145 |
| LBP_cg1476 | Pyruvate kinase | 40 | 1871 |
| LBP_cg1782 | Isoleucyl-tRNA synthetase | 40 | 179 |
| LBP_cg0971 | ATP-dependent Clp protease, ATP-binding subunit ClpE | 39 | 268 |
| LBP_cg0538 | 60 kDa chaperonin | 38 | 2012 |
| LBP_cg0906 | ATP-dependent DNA helicase PcrA | 38 | 101 |
| LBP_cg1546 | Glycyl-tRNA synthetase beta subunit | 37 | 154 |
| LBP_cg1568 | GTP pyrophosphokinase | 36 | 86 |
| LBP_cg1059 | Arginyl-tRNA synthetase | 35 | 117 |
| LBP_cg1144 | Threonine--tRNA ligase 1 | 34 | 280 |
| LBP_cg1607 | Prolyl-tRNA synthetase | 34 | 235 |
| LBP_cg1463 | 30S ribosomal protein S1 | 33 | 521 |
| LBP_cg0619 | Glucosamine--fructose-6-phosphate aminotransferase | 33 | 418 |
| LBP_p3g040 | Aspartate aminotransferase | 33 | 219 |
| LBP_cg0570 | Phosphoglucomutase | 33 | 194 |
| LBP_cg1167 | 6-phosphogluconate dehydrogenase, decarboxylating | 32 | 238 |
| LBP_cg0974 | Phosphoenolpyruvate-protein phosphatase | 32 | 210 |
| LBP_cg0696 | Bifunctional GMP synthase/glutamine amidotransferase protein | 32 | 136 |
| LBP_cg0439 | Cell division protein FtsH, ATP-dependent zinc metallopeptidase | 31 | 172 |
| LBP_cg0730 | Membrane alanine aminopeptidase | 31 | 127 |
| LBP_cg0508 | Ribonucleoside-diphosphate reductase | 31 | 102 |
| LBP_cg1006 | Leucyl-tRNA synthetase | 31 | 98 |
| LBP_cg1489 | Pyruvate,water dikinase | 31 | 65 |
| LBP_cg1726 | Trigger factor | 30 | 498 |
| LBP_cg0489 | Glutamyl-tRNA synthetase | 30 | 160 |
| LBP_cg1189 | Penicillin binding protein 2B | 30 | 65 |
| LBP_cg0006 | DNA gyrase, A subunit | 30 | 80 |
| LBP_cg0591 | Phosphoglycerate kinase | 29 | 553 |
| LBP_cg0911 | Aspartyl/glutamyl-tRNA(Asn/Gln) amidotransferase subunit B | 29 | 169 |
| LBP_cg0005 | DNA gyrase subunit B | 29 | 98 |
| LBP_cg2375 | Integral membrane protein | 29 | 56 |
| LBP_cg0638 | Polyphosphate kinase | 29 | 71 |
| LBP_cg0593 | Enolase 1 | 28 | 1272 |
| LBP_cg1242 | Glycerone kinase | 28 | 156 |
| LBP_cg0878 | Citrate lyase, alpha chain | 28 | 179 |
| LBP_cg2225 | Bifunctional phosphoribosylaminoimidazolecarboxamide formyltransferase/IMP cyclohydrolase | 28 | 154 |
| LBP_cg1910 | Septation ring formation regulator ezrA | 28 | 66 |
| LBP_cg0907 | DNA ligase | 28 | 66 |
| LBP_cg1138 | DNA polymerase I | 28 | 60 |
| LBP_cg1616 | D-lactate dehydrogenase | 27 | 662 |
| LBP_cg0394 | CTP synthase | 27 | 201 |
| LBP_cg1200 | Glutamine synthetase | 27 | 154 |
| LBP_cg0362 | Methionyl-tRNA synthetase | 27 | 94 |
| LBP_cg1429 | DNA topoisomerase IV subunit A | 27 | 69 |
| LBP_cg2880 | putative phosphoketolase 2 | 27 | 85 |
| LBP_cg1613 | Elongation factor Ts | 26 | 586 |
| LBP_cg1751 | GTP-binding protein TypA | 26 | 188 |
| LBP_cg1332 | Maltose phosphorylase | 26 | 118 |
| LBP_cg0442 | Lysyl-tRNA synthetase | 26 | 128 |
| LBP_cg0598 | Ribonuclease R | 26 | 72 |
| LBP_cg2530 | Muramidase (Putative) | 26 | 58 |
| LBP_cg2401 | Anaerobic ribonucleoside triphosphate reductase | 26 | 68 |
| LBP_cg2229 | Phosphoribosylformylglycinamidine synthase 2 | 26 | 60 |
| LBP_cg1727 | Elongation factor Tu | 25 | 1639 |
| LBP_cg2054 | Glucose-6-phosphate isomerase | 25 | 360 |
| LBP_cg1941 | ATP synthase subunit alpha | 25 | 302 |
| LBP_cg1755 | Dihydrolipoyl dehydrogenase | 25 | 256 |
| LBP_cg1756 | Pyruvate dehydrogenase | 25 | 176 |
| LBP_cg2300 | Tyrosyl-tRNA synthetase | 25 | 77 |
| LBP_cg1181 | Phenylalanyl-tRNA synthetase subunit beta | 25 | 72 |
| LBP_cg0232 | Teichoic acid biosynthesis protein | 25 | 54 |
| LBP_cg2799 | Endopeptidase PepO | 25 | 62 |
| LBP_cg1883 | DNA mismatch repair protein mutS | 25 | 50 |
| LBP_cg0981 | Alkaline phosphatase superfamily protein | 25 | 48 |
| LBP_cg1939 | ATP synthase subunit beta | 24 | 480 |
| LBP_cg2094 | NADH peroxidase | 24 | 196 |
| LBP_cg0773 | Asparagine synthase (Glutamine-hydrolysing) | 24 | 157 |
| LBP_cg0618 | Phosphoglucosamine mutase | 24 | 126 |
| LBP_cg0209 | Mannitol-1-phosphate 5-dehydrogenase | 24 | 101 |
| LBP_cg1729 | Metallo-beta-lactamase superfamily protein (Putative) | 24 | 94 |
| LBP_cg2669 | Adenylosuccinate synthetase | 24 | 93 |
| LBP_cg0644 | Pyruvate oxidase | 24 | 77 |
| LBP_cg0154 | Maltose phosphorylase | 24 | 65 |
| LBP_cg0693 | putative DNA helicase | 24 | 62 |
| LBP_cg1862 | MutS2 protein | 24 | 60 |
| LBP_cg1247 | Cell division protein Smc | 24 | 45 |
| LBP_cg0577 | UvrABC system protein A | 24 | 42 |
| LBP_cg1614 | 30S ribosomal protein S2 | 23 | 335 |
| LBP_cg1787 | Cell division protein ftsZ | 23 | 187 |
| LBP_cg0910 | Glutamyl-tRNA(Gln) amidotransferase subunit A | 23 | 140 |
| LBP_cg0148 | Maltose/maltodextrin ABC transporter, substrate binding protein | 23 | 142 |
| LBP_cg1788 | Cell division protein FtsA | 23 | 117 |
| LBP_cg1725 | ATP-dependent Clp protease ATP-binding subunit clpX | 23 | 104 |
| LBP_cg2194 | Glucose-6-phosphate 1-dehydrogenase | 23 | 110 |
| LBP_cg0841 | NADH dehydrogenase | 23 | 63 |
| LBP_cg0963 | Oligopeptide ABC superfamily ATP binding cassette transporter, substrate binding protein | 23 | 55 |
| LBP_cg2287 | hypothetical protein | 23 | 59 |
| LBP_p5g005 | Cation-transporting ATPase, E1-E2 family | 23 | 60 |
| LBP_cg0793 | Acetolactate synthase | 23 | 49 |
| LBP_cg0585 | Lipoprotein, peptide binding protein OppA-like protein | 23 | 41 |
| LBP_cg1604 | DNA polymerase III polC-type | 23 | 37 |
| LBP_cg1924 | D-alanine--D-alanine ligase | 22 | 200 |
| LBP_cg0563 | UTP-glucose-1-phosphate uridylyltransferase | 22 | 230 |
| LBP_cg0465 | Mannose PTS, EIIAB | 22 | 160 |
| LBP_cg0119 | hypothetical protein | 22 | 137 |
| LBP_cg1287 | Acetyl-CoA carboxylase, biotin carboxylase subunit | 22 | 88 |
| LBP_cg1817 | Oligoendopeptidase F | 22 | 55 |
| LBP_cg1250 | Signal recognition particle protein Ffh | 22 | 75 |
| LBP_cg1562 | Aspartyl-tRNA synthetase | 22 | 78 |
| LBP_p6g001 | YkuG protein | 22 | 54 |
| LBP_cg1433 | ATP-dependent protease ATPase subunit HslU | 22 | 76 |
| LBP_cg1579 | D-alanyl transfer protein DltD | 22 | 57 |
| LBP_cg2944 | tRNA uridine 5-carboxymethylaminomethyl modification enzyme mnmG | 22 | 49 |
| LBP_cg2204 | ATP-dependent nuclease, subunit A | 22 | 36 |
| LBP_cg2598 | Guanosine 5'-monophosphate oxidoreductase | 21 | 170 |
| LBP_cg0430 | L-lactate dehydrogenase 1 | 21 | 221 |
| LBP_cg1758 | Pyruvate dehydrogenase complex, E1 component, alpha subunit | 21 | 160 |
| LBP_cg1585 | Chaperone protein dnaJ | 21 | 137 |
| LBP_cg2892 | Oxidoreductase | 21 | 116 |
| LBP_cg2760 | Oxidoreductase | 21 | 120 |
| LBP_cg1116 | Foldase protein prsA 1 | 21 | 99 |
| LBP_cg1377 | Formate--tetrahydrofolate ligase | 21 | 73 |
| LBP_cg0410 | ATP-dependent RNA helicase | 21 | 76 |
| LBP_cg1126 | UDP-N-acetylmuramate--L-alanine ligase | 21 | 69 |
| LBP_cg0458 | Cation transporting P family ATPase | 21 | 38 |
| LBP_cg1477 | 6-phosphofructokinase | 20 | 369 |
| LBP_cg1427 | putative manganese-dependent inorganic pyrophosphatase | 20 | 238 |
| LBP_cg0877 | Citrate lyase, beta chain | 20 | 146 |
| LBP_cg2578 | Cyclopropane-fatty-acyl-phospholipid synthase | 20 | 101 |
| LBP_cg0269 | Acetate kinase | 20 | 98 |
| LBP_cg0836 | DNA-directed RNA polymerase subunit alpha | 20 | 110 |
| LBP_cg2235 | Oxidoreductase (Putative) | 20 | 77 |
| LBP_cg2169 | Muramidase | 20 | 69 |
| LBP_cg0153 | Multiple sugar ABC transporter, ATP-binding protein | 20 | 69 |
| LBP_cg0956 | Peptide chain release factor 3 | 20 | 50 |
| LBP_cg0035 | Serine protease HtrA | 20 | 64 |
| LBP_cg1775 | tRNA-specific 2-thiouridylase mnmA | 20 | 65 |
| LBP_cg1578 | GTP-binding protein lepA 1 | 20 | 54 |
| LBP_cg0756 | Dipeptidase | 20 | 51 |
| LBP_cg2778 | Phosphoenolpyruvate carboxykinase (ATP) | 20 | 35 |
| LBP_cg1462 | GTP-binding protein engA | 20 | 37 |
| LBP_cg2927 | Bifunctional acetaldehyde-CoA/alcohol dehydrogenase | 20 | 38 |
| LBP_cg2920 | Ribokinase | 19 | 193 |
| LBP_cg2116 | Aspartate-semialdehyde dehydrogenase | 19 | 172 |
| LBP_cg1951 | Serine hydroxymethyltransferase | 19 | 120 |
| LBP_cg0606 | Phosphotransacetylase | 19 | 99 |
| LBP_cg1223 | putative quaternary-amine-transporting ATPase | 19 | 103 |
| LBP_cg2875 | L-iditol 2-dehydrogenase | 19 | 96 |
| LBP_cg1563 | Histidyl-tRNA synthetase | 19 | 83 |
| LBP_cg2275 | 6-phospho-beta-glucosidase | 19 | 79 |
| LBP_cg0579 | Argininosuccinate synthase | 19 | 65 |
| LBP_cg2529 | Succinate-semialdehyde dehydrogenase NADP+ | 19 | 59 |
| LBP_cg0979 | Aromatic amino acid aminotransferase | 19 | 62 |
| LBP_cg1762 | Metallo-beta-lactamase | 19 | 51 |
| LBP_cg2668 | Adenylosuccinate lyase | 19 | 70 |
| LBP_cg1791 | UDP-N-acetylmuramoylalanine--D-glutamate ligase | 19 | 53 |
| LBP_cg1430 | DNA topoisomerase IV subunit B | 19 | 61 |
| LBP_cg1885 | 2',3'-cyclic-nucleotide 2'-phosphodiesterase | 19 | 65 |
| LBP_cg0560 | HPr kinase/phosphorylase | 19 | 58 |
| LBP_cg0255 | Transport protein | 19 | 34 |
| LBP_cg1002 | Poly(Glycerol-phosphate) alpha-glucosyltransferase | 19 | 36 |
| LBP_cg1182 | Aminodeoxychorismate lyase | 19 | 36 |
| LBP_cg0590 | Glyceraldehyde 3-phosphate dehydrogenase | 18 | 858 |
| LBP_cg1602 | Transcription elongation factor NusA | 18 | 128 |
| LBP_cg1341 | Asparaginyl-tRNA synthetase 2 | 18 | 150 |
| LBP_cg2600 | Translation-associated GTPase | 18 | 108 |
| LBP_cg0995 | S-adenosylmethionine synthetase | 18 | 88 |
| LBP_cg0799 | Seryl-tRNA synthetase | 18 | 70 |
| LBP_cg2734 | Small heat shock protein | 18 | 132 |
| LBP_cg0832 | Adenylate kinase | 18 | 102 |
| LBP_cg0908 | Lipoprotein | 18 | 56 |
| LBP_cg1544 | RNA polymerase sigma factor | 18 | 78 |
| LBP_cg0880 | Fumarate hydratase | 18 | 62 |
| LBP_cg0684 | GTP-binding protein HflX | 18 | 45 |
| LBP_cg1437 | DNA topoisomerase I | 18 | 48 |
| LBP_cg0924 | ATP-dependent RNA helicase | 18 | 37 |
| LBP_cg0001 | Chromosomal replication initiator protein dnaA | 18 | 33 |
| LBP_cg0512 | DNA-directed DNA polymerase III, gamma/tau subunit | 18 | 26 |
| LBP_cg2862 | Alpha-glucosidase | 18 | 25 |
| LBP_cg0534 | ABC transporter, ATP-binding protein | 18 | 24 |
| LBP_cg1382 | Carbamoyl phosphate synthase large subunit | 18 | 24 |
| LBP_cg0289 | Fructose-bisphosphate aldolase | 17 | 529 |
| LBP_cg1901 | Cell shape determining protein MreB | 17 | 245 |
| LBP_cg1940 | ATP synthase gamma chain | 17 | 158 |
| LBP_cg1757 | Pyruvate dehydrogenase complex, E1 component, beta subunit | 17 | 155 |
| LBP_p2g050 | Pyridine nucleotide-disulfide oxidoreductase family protein | 17 | 146 |
| LBP_cg0482 | Cysteine aminopeptidase | 17 | 139 |
| LBP_cg1928 | ABC superfamily ATP binding cassette transporter, binding protein | 17 | 96 |
| LBP_cg0457 | NH(3)-dependent NAD(+) synthetase | 17 | 88 |
| LBP_cg1482 | Peptidase T | 17 | 83 |
| LBP_cg0453 | N-acetylglucosamine-6-phosphate deacetylase | 17 | 73 |
| LBP_cg0382 | Glucosamine-1-phosphate N-acetyltransferase | 17 | 47 |
| LBP_cg0771 | UDP-N-acetylmuramoylalanyl-D-glutamate--2, 6-diami nopimelate ligase | 17 | 46 |
| LBP_cg1873 | S-adenosylmethionine:tRNA ribosyltransferase-isomerase | 17 | 55 |
| LBP_cg2524 | Asparagine synthase (Glutamine-hydrolysing) | 17 | 60 |
| LBP_cg0930 | Glutamate dehydrogenase (NADP(+)) | 17 | 51 |
| LBP_cg0788 | Transcription regulator | 17 | 37 |
| LBP_cg2827 | Galactose-1-phosphate uridylyltransferase | 17 | 33 |
| LBP_cg1351 | Penicillin binding protein 1A | 17 | 38 |
| LBP_cg1248 | Signal recognition particle receptor FtsY | 17 | 45 |
| LBP_cg1584 | Serine-type D-Ala-D-Ala carboxypeptidase | 17 | 32 |
| LBP_cg0955 | Glutathione reductase | 17 | 40 |
| LBP_cg0288 | Acetaldehyde dehydrogenase | 17 | 30 |
| LBP_cg1868 | ATP-dependent RNA helicase | 17 | 31 |
| LBP_cg0104 | Cation transporting P-type ATPase | 17 | 26 |
| LBP_cg0576 | UvrABC system protein B | 17 | 32 |
| LBP_cg1000 | hypothetical protein | 17 | 25 |
| LBP_cg0592 | Triosephosphate isomerase | 16 | 457 |
| LBP_cg0500 | 50S ribosomal protein L1 | 16 | 396 |
| LBP_cg0814 | 50S ribosomal protein L2 | 16 | 316 |
| LBP_cg0828 | 30S ribosomal protein S5 | 16 | 227 |
| LBP_cg1290 | Enoyl-(Acyl carrier protein) reductase | 16 | 166 |
| LBP_cg0567 | Thioredoxin reductase | 16 | 124 |
| LBP_cg1010 | Dipeptidase PepV | 16 | 113 |
| LBP_cg1720 | Glutamine ABC transporter, substrate binding and permease protein | 16 | 83 |
| LBP_cg2704 | Formate acetyltransferase activating enzyme | 16 | 68 |
| LBP_cg0885 | Malate dehydrogenase | 16 | 54 |
| LBP_cg2666 | Glutathione reductase | 16 | 61 |
| LBP_cg2593 | Serine family D-Ala-D-Ala carboxypeptidase | 16 | 52 |
| LBP_cg0325 | Glutathione reductase | 16 | 62 |
| LBP_cg2828 | UDP-glucose 4-epimerase | 16 | 59 |
| LBP_cg0842 | Lipoprotein | 16 | 65 |
| LBP_cg1457 | Dihydrodipicolinate reductase | 16 | 63 |
| LBP_cg1232 | Methionyl-tRNA formyltransferase | 16 | 63 |
| LBP_cg1394 | DegV family protein | 16 | 58 |
| LBP_cg1954 | Peptide chain release factor 1 | 16 | 50 |
| LBP_cg0183 | Acetate kinase | 16 | 45 |
| LBP_cg2130 | hypothetical protein | 16 | 47 |
| LBP_cg0490 | Cysteine--tRNA ligase | 16 | 40 |
| LBP_cg2057 | putative S-adenosyl-L-methionine-dependent methyltransferase | 16 | 40 |
| LBP_cg0456 | Nicotinate phosphoribosyltransferase | 16 | 34 |
| LBP_cg2911 | Pyruvate oxidase | 16 | 33 |
| LBP_cg0011 | Exopolyphosphatase-related protein (Putative) | 16 | 29 |
| LBP_cg1956 | UDP-N-acetylmuramyl tripeptide synthase (Putative) | 16 | 28 |
| LBP_cg1172 | Exonuclease SbcC | 16 | 23 |
| LBP_cg1917 | Bifunctional glutamate--cysteine ligase/glutathione synthetase | 16 | 23 |
| LBP_cg2327 | Poly(Glycerol-phosphate) alpha-glucosyltransferase | 16 | 30 |
| LBP_cg1912 | 30S ribosomal protein S4 | 15 | 304 |
| LBP_cg0817 | 30S ribosomal protein S3 | 15 | 216 |
| LBP_cg0669 | Glutamine ABC transporter, ATP-binding protein | 15 | 181 |
| LBP_cg2490 | Short-chain dehydrogenase/oxidoreductase | 15 | 170 |
| LBP_cg1783 | Cell division initiation protein DivIVA | 15 | 114 |
| LBP_cg1942 | ATP synthase subunit delta | 15 | 110 |
| LBP_cg1886 | Protein recA | 15 | 87 |
| LBP_cg0313 | Cell division protein SufI | 15 | 98 |
| LBP_cg2274 | 6-phospho-beta-glucosidase | 15 | 91 |
| LBP_cg2833 | Sugar transport protein | 15 | 60 |
| LBP_cg0345 | Tryptophanyl-tRNA synthetase II | 15 | 61 |
| LBP_cg1611 | Ribosome-recycling factor | 15 | 77 |
| LBP_cg0117 | Oxidoreductase | 15 | 45 |
| LBP_cg0384 | Ribose-phosphate pyrophosphokinase 1 | 15 | 63 |
| LBP_cg1811 | hypothetical protein | 15 | 50 |
| LBP_cg1428 | Transcription regulator | 15 | 52 |
| LBP_cg0367 | DNAse (Putative) | 15 | 55 |
| LBP_cg1156 | GTP-binding protein YqeH | 15 | 44 |
| LBP_cg1201 | hypothetical protein | 15 | 51 |
| LBP_cg2900 | Catalase | 15 | 45 |
| LBP_cg2595 | Response regulator | 15 | 51 |
| LBP_cg0302 | Teichoic acids export ATP-binding protein TagH | 15 | 51 |
| LBP_cg0152 | Alpha-amylase | 15 | 37 |
| LBP_cg0462 | Homoserine dehydrogenase | 15 | 39 |
| LBP_cg1790 | UDP-N-acetylglucosamine--N-acetylmuramyl-(pentapeptide) pyrophosphoryl-undecaprenol N-acetylglucosamine transferase | 15 | 32 |
| LBP_cg2232 | Phosphoribosylaminoimidazole-succinocarboxamide synthase | 15 | 34 |
| LBP_cg1907 | putative tRNA sulfurtransferase | 15 | 32 |
| LBP_cg0754 | Aspartate--ammonia ligase | 15 | 46 |
| LBP_cg1447 | S41 family carboxy-terminal processing peptidase | 15 | 30 |
| LBP_cg0026 | Maltose phosphorylase | 15 | 28 |
| LBP_cg1356 | Site-specific DNA-methyltransferase | 15 | 26 |
| LBP_cg2156 | Pyruvate oxidase | 15 | 25 |
| LBP_cg2462 | ABC transporter, ATP-binding and permease protein | 15 | 29 |
| LBP_cg1793 | Penicillin binding protein 2B | 15 | 23 |
| LBP_cg1180 | Phenylalanyl-tRNA synthetase alpha chain | 15 | 30 |
| LBP_cg1217 | Exodeoxyribonuclease 7 large subunit | 15 | 19 |
| LBP_cg0251 | Lactate dehydrogenase (Oxidoreductase) | 15 | 18 |
| LBP_cg1233 | rRNA methylase (Putative) | 15 | 21 |
| LBP_cg1193 | Glucokinase | 14 | 101 |
| LBP_cg0821 | 50S ribosomal protein L14 | 14 | 149 |
| LBP_cg0562 | Glycerol-3-phosphate dehydrogenase NAD(P)+ | 14 | 81 |
| LBP_cg1958 | Mannose-6-phosphate isomerase | 14 | 107 |
| LBP_cg1244 | Phosphate acyltransferase | 14 | 68 |
| LBP_cg1935 | Cell shape determining protein MreB | 14 | 66 |
| LBP_cg0273 | NADH dehydrogenase | 14 | 63 |
| LBP_cg2829 | Galactokinase | 14 | 73 |
| LBP_cg1612 | Uridylate kinase | 14 | 98 |
| LBP_cg0219 | Cystathionine beta-lyase | 14 | 70 |
| LBP_cg1705 | Fructose PTS, EIIABC | 14 | 65 |
| LBP_cg2670 | GMP reductase | 14 | 59 |
| LBP_cg1587 | Protein grpE | 14 | 72 |
| LBP_cg0670 | Glutamine ABC transporter, substrate binding protein | 14 | 56 |
| LBP_cg0522 | UDP-glucose 4-epimerase | 14 | 48 |
| LBP_cg1927 | L-2-hydroxyisocaproate dehydrogenase | 14 | 56 |
| LBP_cg1169 | Response regulator | 14 | 83 |
| LBP_p3g014 | RepB | 14 | 58 |
| LBP_cg1288 | Acetyl-coenzyme A carboxylase carboxyl transferase subunit beta 2 | 14 | 55 |
| LBP_cg0881 | Succinate dehydrogenase | 14 | 39 |
| LBP_cg1702 | GTPase obg | 14 | 47 |
| LBP_cg1858 | Nucleoside-triphosphatase | 14 | 45 |
| LBP_cg2602 | Chromosome partitioning protein, DNA-binding protein | 14 | 40 |
| LBP_cg1131 | ABC transporter component (Putative) | 14 | 36 |
| LBP_cg0905 | Phosphoribosylaminoimidazole carboxylase, ATPase subunit | 14 | 37 |
| LBP_cg2266 | Outer surface protein | 14 | 31 |
| LBP_cg0600 | Cell surface protein | 14 | 18 |
| LBP_cg1392 | Fibronectin binding protein A | 14 | 25 |
| LBP_cg1454 | ABC superfamily ATP binding cassette transporter, ABC protein | 14 | 26 |
| LBP_cg0432 | Transcription-repair coupling factor | 14 | 21 |
| LBP_cg2125 | Phosphatidylglycerol--membrane-oligosaccharide glycerophosphotransferase | 14 | 20 |
| LBP_cg0811 | 50S ribosomal protein L3 | 13 | 443 |
| LBP_cg0812 | 50S ribosomal protein L4 | 13 | 297 |
| LBP_cg1845 | hypothetical protein | 13 | 92 |
| LBP_cg1704 | 1-phosphofructokinase | 13 | 64 |
| LBP_cg1950 | Uracil phosphoribosyltransferase | 13 | 86 |
| LBP_cg0030 | Response regulator | 13 | 90 |
| LBP_cg1844 | Ribokinase | 13 | 58 |
| LBP_p1g047 | PTS sugar transporter subunit IIA | 13 | 52 |
| LBP_cg1766 | Ribose-phosphate pyrophosphokinase 2 | 13 | 66 |
| LBP_cg0197 | Oxidoreductase | 13 | 53 |
| LBP_cg1582 | D-alanine--poly(phosphoribitol) ligase subunit 1 | 13 | 54 |
| LBP_cg2803 | NADH oxidase | 13 | 58 |
| LBP_cg2702 | putative beta-lactamase | 13 | 47 |
| LBP_cg2689 | Dihydropteroate synthase | 13 | 42 |
| LBP_cg2272 | ABC superfamily ATP binding cassette transporter, ABC protein | 13 | 39 |
| LBP_cg2937 | Cysteine aminopeptidase | 13 | 41 |
| LBP_cg0409 | UDP-N-acetylmuramoyl-tripeptide--D-alanyl-D-alanine ligase | 13 | 35 |
| LBP_cg0320 | Ribosylpyrimidine nucleosidase | 13 | 36 |
| LBP_cg0976 | Glycosyltransferase | 13 | 29 |
| LBP_cg2059 | Response regulator | 13 | 31 |
| LBP_cg2168 | Lipoate-protein ligase | 13 | 33 |
| LBP_cg0617 | YbbR like protein | 13 | 25 |
| LBP_cg2909 | Pyruvate oxidase | 13 | 23 |
| LBP_cg1882 | DNA mismatch repair protein | 13 | 29 |
| LBP_cg0646 | Putative pyruvate oxidase | 13 | 33 |
| LBP_cg1235 | Serine/threonine protein kinase | 13 | 25 |
| LBP_cg1887 | Competence-damage protein | 13 | 27 |
| LBP_cg1342 | DNA replication protein DnaD | 13 | 24 |
| LBP_cg0967 | ABC superfamily ATP binding cassette transporter, ABC protein | 13 | 29 |
| LBP_cg2604 | Chromosome partitioning protein, DNA-binding protein | 13 | 28 |
| LBP_cg2855 | 6-phospho-beta-glucosidase | 13 | 21 |
| LBP_cg2000 | Prophage protein | 13 | 22 |
| LBP_cg0413 | Alanine racemase | 13 | 27 |
| LBP_cg0226 | Alpha, alpha-phosphotrehalase | 13 | 33 |
| LBP_cg0210 | hypothetical protein | 13 | 21 |
| LBP_cg1338 | DNA-directed DNA polymerase III epsilon subunit | 13 | 15 |
| LBP_cg2328 | Poly(Glycerol-phosphate) alpha-glucosyltransferase | 13 | 28 |
| LBP_cg0017 | Lipoprotein, peptide binding protein OppA-like protein | 13 | 25 |
| LBP_cg1142 | Replication initiation and membrane attachment protein DnaB | 13 | 22 |
| LBP_p3g035 | LtrC-like protein | 13 | 26 |
| LBP_cg2787 | Iron dependent peroxidase (Putative) | 13 | 26 |
| LBP_cg1900 | Cell shape determining protein MreC | 13 | 20 |
| LBP_cg1301 | Cyclopropane-fatty-acyl-phospholipid synthase | 13 | 27 |
| LBP_cg0721 | Alkaline shock protein | 12 | 440 |
| LBP_cg2574 | 2,3-bisphosphoglycerate-dependent phosphoglycerate mutase 2 | 12 | 410 |
| LBP_cg0823 | 50S ribosomal protein L5 | 12 | 274 |
| LBP_cg1851 | hypothetical protein | 12 | 184 |
| LBP_cg0012 | 50S ribosomal protein L9 | 12 | 188 |
| LBP_cg0501 | 50S ribosomal protein L10 | 12 | 155 |
| LBP_cg0871 | L-lactate dehydrogenase 2 | 12 | 121 |
| LBP_cg0483 | Ribose-5-phosphate isomerase A | 12 | 62 |
| LBP_cg1848 | Catabolite control protein A | 12 | 72 |
| LBP_cg1781 | Diaminopimelate epimerase | 12 | 63 |
| LBP_cg0578 | S-ribosylhomocysteine lyase | 12 | 72 |
| LBP_cg0815 | 30S ribosomal protein S19 | 12 | 80 |
| LBP_cg1849 | Xaa-Pro dipeptidase | 12 | 67 |
| LBP_cg1908 | Cysteine desulfurase | 12 | 47 |
| LBP_cg1308 | hypothetical protein | 12 | 55 |
| LBP_cg0601 | Glutamine ABC transporter, substrate binding and permease protein | 12 | 41 |
| LBP_cg0002 | DNA-directed DNA polymerase III, beta chain | 12 | 48 |
| LBP_cg1549 | GTP-binding protein era | 12 | 41 |
| LBP_cg0381 | Purine operon repressor | 12 | 41 |
| LBP_cg0637 | Exopolyphosphatase | 12 | 33 |
| LBP_cg0225 | GntR family transcriptional regulator | 12 | 39 |
| LBP_cg1547 | Glycyl-tRNA synthetase alpha subunit | 12 | 35 |
| LBP_cg1558 | putative endonuclease 4 | 12 | 36 |
| LBP_cg0488 | PilT protein domain protein | 12 | 38 |
| LBP_cg1484 | S-adenosyl-L-methionine-dependent methyltransferase | 12 | 38 |
| LBP_cg1225 | Glycine betaine/carnitine/choline ABC superfamily ATP binding cassette transporter, substrate binding protein | 12 | 29 |
| LBP_cg1143 | Primosomal protein DnaI | 12 | 31 |
| LBP_cg2945 | tRNA modification GTPase mnmE | 12 | 23 |
| LBP_cg2038 | hypothetical protein | 12 | 36 |
| LBP_cg1134 | ABC transporter component, iron regulated (Putative) | 12 | 25 |
| LBP_cg0913 | RNA methyltransferase | 12 | 27 |
| LBP_cg1961 | Branched-chain-amino-acid aminotransferase | 12 | 25 |
| LBP_cg1952 | SUA5 family translation factor | 12 | 32 |
| LBP_cg0016 | Gamma-glutamyl phosphate reductase | 12 | 25 |
| LBP_cg1588 | Heat-inducible transcription repressor hrcA | 12 | 28 |
| LBP_cg0401 | UDP-N-acetylglucosamine 1-carboxyvinyltransferase 2 | 12 | 25 |
| LBP_cg0784 | Metal-dependent regulator | 12 | 30 |
| LBP_cg0583 | hypothetical protein | 12 | 27 |
| LBP_cg2142 | 1-deoxy-D-xylulose-5-phosphate synthase | 12 | 26 |
| LBP_p2g040 | ATPase involved in chromosome partitioning | 12 | 25 |
| LBP_cg1222 | DNA repair protein RecN | 12 | 24 |
| LBP_cg2186 | Hemolysin | 12 | 22 |
| LBP_cg0293 | Dipeptidase | 12 | 21 |
| LBP_cg1460 | Tetratricopeptide repeat family protein | 12 | 25 |
| LBP_cg0612 | UDP-N-acetylenolpyruvoylglucosamine reductase | 12 | 23 |
| LBP_cg0639 | Exopolyphosphatase | 12 | 23 |
| LBP_cg1001 | Poly(Glycerol-phosphate) alpha-glucosyltransferase | 12 | 20 |
| LBP_cg0966 | Oligopeptide ABC transporter, ATP-binding protein | 12 | 18 |
| LBP_cg0402 | Transcription termination factor Rho | 12 | 17 |
| LBP_cg1298 | Integral membrane protein | 12 | 18 |
| LBP_cg1329 | tRNA/rRNA methyltransferase | 12 | 17 |
| LBP_cg0469 | Transcription regulator | 12 | 14 |
| LBP_cg0502 | 50S ribosomal protein L7/L12 | 11 | 606 |
| LBP_cg0499 | 50S ribosomal protein L11 | 11 | 167 |
| LBP_cg1283 | 3-oxoacyl-(Acyl-carrier protein) reductase | 11 | 60 |
| LBP_cg1184 | Transcription elongation factor greA 2 | 11 | 69 |
| LBP_cg0230 | Glycerol-3-phosphate cytidylyltransferase | 11 | 74 |
| LBP_cg1464 | Cytidylate kinase | 11 | 46 |
| LBP_cg1451 | DegV family protein | 11 | 42 |
| LBP_cg2613 | Amino acid ABC superfamily ATP binding cassette transporter, binding protein | 11 | 48 |
| LBP_cg1289 | Acetyl-CoA carboxylase, carboxyl transferase subunit alpha | 11 | 44 |
| LBP_cg1354 | Cell cycle protein gpsB | 11 | 44 |
| LBP_cg0492 | tRNA/rRNA methyltransferase | 11 | 32 |
| LBP_cg0535 | Redox-sensing transcriptional repressor rex | 11 | 41 |
| LBP_cg1930 | Methionine import ATP-binding protein MetN 2 | 11 | 35 |
| LBP_cg0772 | Aspartokinase | 11 | 36 |
| LBP_cg1440 | GTPase | 11 | 34 |
| LBP_cg1925 | Oxidoreductase | 11 | 29 |
| LBP_cg0582 | nucleotide-binding protein | 11 | 38 |
| LBP_cg2238 | Protein-tyrosine phosphatase (Putative) | 11 | 25 |
| LBP_cg0923 | Oxidoreductase (Putative) | 11 | 28 |
| LBP_cg0321 | Short chain dehydrogenase | 11 | 20 |
| LBP_cg2768 | Extracellular protein | 11 | 24 |
| LBP_cg0406 | putative membrane protein LemA | 11 | 30 |
| LBP_cg1675 | hypothetical protein | 11 | 26 |
| LBP_cg0231 | Teichoic acid biosynthesis protein | 11 | 25 |
| LBP_cg0580 | Argininosuccinate lyase | 11 | 23 |
| LBP_cg1869 | Exopolyphosphatase-related protein (Putative) | 11 | 28 |
| LBP_cg2259 | Hydrolase, HAD superfamily, Cof family | 11 | 33 |
| LBP_cg0874 | Malic enzyme, NAD-dependent | 11 | 26 |
| LBP_cg2628 | putative RNA methyltransferase | 11 | 21 |
| LBP_cg1556 | Putative phosphotransferase | 11 | 26 |
| LBP_cg2273 | putative D-serine dehydratase | 11 | 25 |
| LBP_cg0327 | Glycerol-3-phosphate dehydrogenase | 11 | 20 |
| LBP_cg2151 | Oligoendopeptidase F | 11 | 20 |
| LBP_cg1724 | putative GTP-binding protein engB | 11 | 22 |
| LBP_cg0975 | Glycosyltransferase | 11 | 17 |
| LBP_cg1202 | hypothetical protein | 11 | 27 |
| LBP_cg1624 | Hydroxymethylglutaryl-CoA synthase | 11 | 21 |
| LBP_cg2386 | Excinuclease ABC, subunit A | 11 | 18 |
| LBP_cg0160 | Sucrose PTS, EIIBCA | 11 | 19 |
| LBP_cg2487 | ABC superfamily ATP binding cassette transporter, membrane protein | 11 | 16 |
| LBP_cg2383 | ABC superfamily ATP binding cassette transporter, ABC protein | 11 | 15 |
| LBP_cg2258 | Threonine synthase | 11 | 18 |
| LBP_cg0546 | Ribosomal protein S30EA | 10 | 507 |
| LBP_cg0826 | 50S ribosomal protein L6 | 10 | 338 |
| LBP_p6g011 | Stress induced DNA binding protein | 10 | 416 |
| LBP_cg0722 | Alkaline shock protein | 10 | 194 |
| LBP_cg0808 | 30S ribosomal protein S7 | 10 | 207 |
| LBP_cg1455 | hypothetical protein | 10 | 111 |
| LBP_cg1722 | hypothetical protein | 10 | 72 |
| LBP_cg0825 | 30S ribosomal protein S8 | 10 | 95 |
| LBP_cg1340 | Aspartate aminotransferase | 10 | 60 |
| LBP_cg0467 | Mannose PTS, EIID | 10 | 58 |
| LBP_cg1554 | Glutaminyl-tRNA synthase b subunit | 10 | 46 |
| LBP_cg0604 | HAD superfamily hydrolase | 10 | 37 |
| LBP_cg0440 | 33 kDa chaperonin | 10 | 39 |
| LBP_cg0227 | Phosphoenolpyruvate-dependent sugar PTS family porter EIIABC, trhalose specific | 10 | 23 |
| LBP_cg2657 | DegV family protein | 10 | 34 |
| LBP_cg0516 | Thymidylate kinase | 10 | 37 |
| LBP_cg1030 | hypothetical protein | 10 | 37 |
| LBP_cg0647 | Proline iminopeptidase | 10 | 37 |
| LBP_cg0201 | Glucosamine-6-phosphate deaminase | 10 | 32 |
| LBP_cg2313 | Purine nucleosidase | 10 | 26 |
| LBP_cg2603 | Chromosome partitioning protein, membrane-associated ATPase | 10 | 30 |
| LBP_cg1897 | Septum site-determining protein MinD | 10 | 32 |
| LBP_cg0054 | Beta-phosphoglucomutase | 10 | 35 |
| LBP_cg1228 | Guanylate kinase | 10 | 39 |
| LBP_cg0873 | Citrate lyase regulator | 10 | 30 |
| LBP_cg1777 | Cysteine desulfurase | 10 | 23 |
| LBP_cg0912 | Diacylglycerol kinase | 10 | 30 |
| LBP_cg0901 | Oxidoreductase (Putative) | 10 | 35 |
| LBP_cg1159 | HD superfamily hydrolase | 10 | 26 |
| LBP_cg1859 | Glutamate racemase | 10 | 22 |
| LBP_cg1893 | Aspartokinase | 10 | 26 |
| LBP_cg1408 | Lipoprotein | 10 | 31 |
| LBP_cg2233 | Phosphoribosylaminoimidazole carboxylase ATPase subunit | 10 | 21 |
| LBP_cg1872 | Queuine tRNA-ribosyltransferase | 10 | 26 |
| LBP_cg1246 | Ribonuclease 3 | 10 | 25 |
| LBP_cg2630 | hypothetical protein | 10 | 22 |
| LBP_cg1590 | FAD synthetase | 10 | 33 |
| LBP_cg0353 | tRNA-dihydrouridine synthase | 10 | 25 |
| LBP_cg2776 | Transcription regulator | 10 | 27 |
| LBP_cg0013 | Replicative DNA helicase DnaC | 10 | 17 |
| LBP_p3g041 | AAE family aspartate:alanine exchanger | 10 | 21 |
| LBP_cg1130 | ABC transporter, ATP-binding protein | 10 | 24 |
| LBP_cg1374 | Oxidoreductase | 10 | 21 |
| LBP_cg0477 | Transcription regulator | 10 | 15 |
| LBP_cg2681 | hypothetical protein | 10 | 23 |
| LBP_cg2405 | tRNA-dihydrouridine synthase | 10 | 23 |
| LBP_cg1469 | Pseudouridine synthase | 10 | 19 |
| LBP_cg1436 | Methylenetetrahydrofolate--tRNA-(uracil-5-)-methyltransferase trmFO | 10 | 15 |
| LBP_cg2835 | Galactose operon repressor | 10 | 16 |
| LBP_cg1802 | DNA translocase ftsK | 10 | 17 |
| LBP_cg2060 | hypothetical protein | 10 | 16 |
| LBP_cg0103 | Halo peroxidase | 10 | 16 |
| LBP_cg2205 | ATP-dependent helicase/deoxyribonuclease subunit B | 10 | 16 |
| LBP_cg2378 | DNA-entry nuclease | 10 | 18 |
| LBP_cg0454 | Transcription regulator | 10 | 20 |
| LBP_cg2743 | Copper transporting ATPase | 10 | 15 |
| LBP_cg2486 | ABC superfamily ATP binding cassette transporter, membrane protein | 10 | 12 |
| LBP_cg0179 | Aryl-alcohol dehydrogenase family enzyme | 10 | 18 |
| LBP_cg0474 | Acetyl-CoA carboxylase, biotin carboxylase subunit | 10 | 17 |
| LBP_cg1125 | Cell division protein FtsK | 10 | 16 |
| LBP_cg0894 | Cytochrome D ABC transporter, ATP-binding and permease protein | 10 | 14 |
| LBP_cg0797 | Serine-type D-Ala-D-Ala carboxypeptidase | 10 | 16 |
| LBP_cg0146 | Alpha-glucosidase | 10 | 11 |
| LBP_cg1052 | Arylsulfate sulfotransferase | 10 | 12 |
| LBP_cg2781 | Extracellular protein, gamma-D-glutamate-meso-diaminopimelate muropeptidase (Putative) | 9 | 228 |
| LBP_cg0834 | 30S ribosomal protein S13 | 9 | 254 |
| LBP_cg1920 | Universal stress protein UspA | 9 | 184 |
| LBP_cg0813 | 50S ribosomal protein L23 | 9 | 165 |
| LBP_cg0109 | Small heat shock protein | 9 | 100 |
| LBP_cg0816 | 50S ribosomal protein L22 | 9 | 111 |
| LBP_cg1213 | Elongation factor P | 9 | 107 |
| LBP_cg0587 | ATP-dependent Clp protease proteolytic subunit | 9 | 86 |
| LBP_cg0837 | 50S ribosomal protein L17 | 9 | 116 |
| LBP_cg2084 | N-acetylglucosamine and glucose PTS, EIICBA | 9 | 31 |
| LBP_cg2185 | hypothetical protein | 9 | 47 |
| LBP_cg0656 | hypothetical protein | 9 | 48 |
| LBP_cg0220 | Cysteine synthase | 9 | 40 |
| LBP_cg1905 | Thiol peroxidase | 9 | 48 |
| LBP_cg1011 | putative universal stress protein | 9 | 35 |
| LBP_cg0849 | 50S ribosomal protein L13 | 9 | 55 |
| LBP_cg1090 | Nucleoside deoxyribosyltransferase | 9 | 35 |
| LBP_cg1280 | 3-oxoacyl-(Acyl carrier protein) synthase III | 9 | 31 |
| LBP_cg0404 | hypothetical protein | 9 | 32 |
| LBP_cg0602 | Glutamine ABC transporter, ATP-binding protein | 9 | 32 |
| LBP_cg0548 | Peptide chain release factor 2 | 9 | 28 |
| LBP_cg1730 | Dihydrodipicolinate synthase | 9 | 18 |
| LBP_cg1525 | Oxidoreductase | 9 | 22 |
| LBP_cg1500 | Succinyl-diaminopimelate desuccinylase | 9 | 28 |
| LBP_cg2096 | 3-hydroxyisobutyrate dehydrogenase | 9 | 27 |
| LBP_cg1474 | RNA-binding protein | 9 | 24 |
| LBP_cg0557 | Phosphate transport system protein | 9 | 29 |
| LBP_cg0429 | ErfK/YbiS/YcfS/YnhG | 9 | 21 |
| LBP_cg1684 | Bifunctional S24 family peptidase/transcriptional regulator | 9 | 20 |
| LBP_cg1795 | Ribosomal RNA small subunit methyltransferase H | 9 | 14 |
| LBP_cg2153 | hypothetical protein | 9 | 18 |
| LBP_cg1317 | Dipeptidase | 9 | 18 |
| LBP_cg1083 | 3'-to-5' exonuclease | 9 | 22 |
| LBP_cg0369 | Ribosomal RNA small subunit methyltransferase A | 9 | 18 |
| LBP_cg0221 | Methionine aminopeptidase | 9 | 22 |
| LBP_cg0472 | 3-oxoacyl-acyl-carrier-protein | 9 | 15 |
| LBP_cg0688 | Phosphoglycerate mutase | 9 | 19 |
| LBP_cg0550 | Response regulator | 9 | 17 |
| LBP_cg1042 | hypothetical protein | 9 | 19 |
| LBP_cg2519 | Extracellular protein (Putative) | 9 | 17 |
| LBP_cg1865 | Putative Holliday junction resolvase | 9 | 21 |
| LBP_cg1776 | hypothetical protein | 9 | 23 |
| LBP_cg0323 | Glycine betaine/carnitine/choline ABC transporter, substrate binding and permease protein | 9 | 18 |
| LBP_cg1699 | Ribonuclease Z | 9 | 17 |
| LBP_cg1830 | HAD superfamily sugar phosphatase | 9 | 15 |
| LBP_cg1835 | Acetate kinase | 9 | 15 |
| LBP_cg2615 | ABC superfamily ATP binding cassette transporter, ABC protein | 9 | 17 |
| LBP_cg2411 | 3-octaprenyl-4-hydroxybenzoate carboxy-lyase | 9 | 13 |
| LBP_cg1736 | dCMP deaminase | 9 | 17 |
| LBP_cg0589 | Central glycolytic protein regulator | 9 | 15 |
| LBP_cg0529 | Glycoprotein endopeptidase | 9 | 12 |
| LBP_cg2814 | Ubiquinone biosynthesis protein UbiB | 9 | 12 |
| LBP_cg1198 | tRNA dimethylallyltransferase | 9 | 12 |
| LBP_cg0978 | Serine-type D-Ala-D-Ala carboxypeptidase | 9 | 9 |
| LBP_cg1415 | Ribitolphosphotransferase | 9 | 14 |
| LBP_cg1559 | Teichoic acid biosynthesis protein | 9 | 14 |
| LBP_cg0844 | Thiamin biosynthesis lipoprotein ApbE | 9 | 14 |
| LBP_cg1049 | Sulfate adenylyltransferase | 9 | 11 |
| LBP_cg1619 | 1-acylglycerol-3-phosphate O-acyltransferase | 9 | 15 |
| LBP_cg0695 | Pantothenate kinase | 9 | 10 |
| LBP_cg0426 | Ornithine carbamoyltransferase | 9 | 9 |
| LBP_cg1039 | Excinuclease ABC, subunit A | 9 | 9 |
| LBP_cg0399 | hypothetical protein | 9 | 12 |
| LBP_cg1461 | DNA-binding protein II | 8 | 273 |
| LBP_cg0785 | Cold shock protein CspC | 8 | 231 |
| LBP_cg0830 | 50S ribosomal protein L15 | 8 | 158 |
| LBP_cg1943 | ATP synthase subunit b | 8 | 121 |
| LBP_cg1284 | 3-oxoacyl-acyl-carrier protein | 8 | 91 |
| LBP_cg0914 | Transcription regulator | 8 | 76 |
| LBP_cg1279 | (3R)-hydroxymyristoyl-acyl-carrier-protein | 8 | 78 |
| LBP_cg2358 | putative universal stress protein | 8 | 52 |
| LBP_cg1889 | hypothetical protein | 8 | 52 |
| LBP_cg2187 | Ribonucleotide reductase | 8 | 50 |
| LBP_cg0810 | 30S ribosomal protein S10 | 8 | 78 |
| LBP_cg2366 | NADP oxidoreductase coenzyme F420-dependent | 8 | 49 |
| LBP_cg0986 | Acetyltransferase | 8 | 45 |
| LBP_cg1346 | putative universal stress protein | 8 | 39 |
| LBP_cg1719 | Glutamine ABC transporter, ATP-binding protein | 8 | 44 |
| LBP_cg1778 | Methylthioadenosine nucleosidase | 8 | 45 |
| LBP_cg0807 | 30S ribosomal protein S12 | 8 | 55 |
| LBP_cg0753 | Asparaginyl-tRNA synthetase | 8 | 44 |
| LBP_cg1080 | hypothetical protein | 8 | 40 |
| LBP_cg1695 | Adenine phosphoribosyltransferase | 8 | 36 |
| LBP_cg1255 | 50S ribosomal protein L19 | 8 | 50 |
| LBP_cg2311 | ABC transporter, ATP-binding protein | 8 | 29 |
| LBP_cg1282 | (Acyl-carrier protein) S-malonyltransferase | 8 | 25 |
| LBP_cg0398 | L-serine dehydratase, alpha subunit | 8 | 24 |
| LBP_cg2242 | ABC transporter, ATP-binding protein | 8 | 19 |
| LBP_cg1854 | Putative N-acetyldiaminopimelate deacetylase | 8 | 38 |
| LBP_cg2236 | Oxidoreductase (Putative) | 8 | 24 |
| LBP_cg1124 | Phenylalanine--tRNA ligase beta subunit | 8 | 29 |
| LBP_cg1819 | Adapter protein mecA | 8 | 20 |
| LBP_cg0324 | ABC superfamily ATP binding cassette transporter, ABC protein | 8 | 19 |
| LBP_cg2672 | Cardiolipin synthetase 2 | 8 | 19 |
| LBP_cg2832 | Alpha-galactosidase | 8 | 17 |
| LBP_cg1955 | Thymidine kinase | 8 | 19 |
| LBP_cg2577 | hypothetical protein | 8 | 13 |
| LBP_cg2917 | Sorbitol-6-phosphate 2-dehydrogenase | 8 | 16 |
| LBP_cg0949 | L-2-hydroxyisocaproate dehydrogenase | 8 | 16 |
| LBP_cg0487 | DNA repair protein radA | 8 | 17 |
| LBP_cg1453 | Thymidylate synthase | 8 | 17 |
| LBP_p1g017 | putative universal stress protein | 8 | 19 |
| LBP_cg1158 | putative nicotinate-nucleotide adenylyltransferase | 8 | 18 |
| LBP_cg2178 | putative transcriptional regulator | 8 | 16 |
| LBP_cg1610 | Undecaprenyl pyrophosphate synthase | 8 | 19 |
| LBP_cg0031 | Sensor protein | 8 | 12 |
| LBP_cg2224 | Phosphoribosylamine--glycine ligase | 8 | 12 |
| LBP_cg0438 | Hypoxanthine-guanine phosphoribosyltransferase | 8 | 24 |
| LBP_cg0518 | DNA polymerase III subunit delta' | 8 | 13 |
| LBP_cg1122 | tRNA (guanine-N(7)-)-methyltransferase | 8 | 19 |
| LBP_cg2345 | Signal peptidase I | 8 | 15 |
| LBP_cg0326 | Glycerol kinase 1 | 8 | 16 |
| LBP_cg1815 | GTP pyrophosphokinase (Putative) | 8 | 17 |
| LBP_cg0903 | Xanthine phosphoribosyltransferase | 8 | 18 |
| LBP_cg1079 | Penicillin binding protein 2A | 8 | 16 |
| LBP_cg2737 | NmrA family NAD-dependent epimerase/dehydratase:3-beta hydroxysteroid dehydrogenase/isomerase | 8 | 12 |
| LBP_cg1696 | Single-strand DNA-specific exonuclease RecJ | 8 | 11 |
| LBP_cg1008 | Pseudouridine synthase | 8 | 15 |
| LBP_cg1754 | Malate/lactate dehydrogenase | 8 | 15 |
| LBP_cg0094 | Phosphomethylpyrimidine kinase | 8 | 14 |
| LBP_cg2310 | ABC transporter, permease protein (Putative) | 8 | 13 |
| LBP_cg0078 | Extracellular protein, peptide binding protein OppA | 8 | 13 |
| LBP_cg1963 | Lipoprotein | 8 | 10 |
| LBP_cg0132 | Short-chain dehydrogenase/oxidoreductase | 8 | 20 |
| LBP_cg1367 | Lysin | 8 | 16 |
| LBP_p3g028 | Nickase | 8 | 10 |
| LBP_cg0690 | Na(+)/H(+) antiporter | 8 | 12 |
| LBP_cg1441 | putative 5-methyltetrahydropteroyltriglutamate--homocysteine S-methyltransferase | 8 | 11 |
| LBP_cg1076 | Aminotransferase | 8 | 10 |
| LBP_cg2385 | Integral membrane protein | 8 | 10 |
| LBP_cg1767 | Exodeoxyribonuclease V, alpha chain | 8 | 9 |
| LBP_cg1231 | Primosomal replication protein n | 8 | 9 |
| LBP_cg1434 | ATP-dependent protease subunit HslV | 8 | 9 |
| LBP_cg2496 | 1,3-propanediol dehydrogenase | 8 | 11 |
| LBP_cg2753 | Cell surface hydrolase, membrane-bound (Putative) | 8 | 9 |
| LBP_cg2277 | Cellobiose PTS, EIIB | 7 | 258 |
| LBP_cg0827 | 50S ribosomal protein L18 | 7 | 96 |
| LBP_cg0010 | 30S ribosomal protein S18 | 7 | 117 |
| LBP_cg2606 | Nucleoside transport protein | 7 | 54 |
| LBP_cg1147 | 50S ribosomal protein L20 | 7 | 65 |
| LBP_cg0507 | Ribonucleotide-diphosphate reductase subunit beta | 7 | 46 |
| LBP_cg1861 | Thioredoxin | 7 | 42 |
| LBP_cg2202 | hypothetical protein | 7 | 33 |
| LBP_cg1432 | Aldose 1-epimerase | 7 | 44 |
| LBP_cg0909 | Aspartyl/glutamyl-tRNA(Asn/Gln) amidotransferase subunit C | 7 | 37 |
| LBP_cg1827 | Peptidylprolyl isomerase | 7 | 41 |
| LBP_cg0850 | 30S ribosomal protein S9 | 7 | 51 |
| LBP_cg2248 | Universal stress protein UspA | 7 | 41 |
| LBP_cg2834 | Aldose 1-epimerase | 7 | 31 |
| LBP_cg2159 | S9 family serine peptidase | 7 | 33 |
| LBP_cg1286 | (3R)-hydroxymyristoyl-(Acyl carrier protein) dehydratase | 7 | 33 |
| LBP_cg1822 | hypothetical protein | 7 | 39 |
| LBP_cg0436 | RNA-binding protein | 7 | 40 |
| LBP_cg1237 | Ribulose-phosphate 3-epimerase | 7 | 29 |
| LBP_cg2166 | hypothetical protein | 7 | 16 |
| LBP_cg2183 | hypothetical protein | 7 | 22 |
| LBP_cg2877 | Galactitol PTS, EIIB | 7 | 28 |
| LBP_cg0206 | Protein-N(Pi)-phosphohistidine--sugar phosphotransferase | 7 | 17 |
| LBP_cg1016 | Deoxyguanosine kinase | 7 | 20 |
| LBP_cg0649 | Acyltransferase (Putative) | 7 | 24 |
| LBP_cg1163 | Nucleic acid-binding protein | 7 | 22 |
| LBP_cg1574 | hypothetical protein | 7 | 28 |
| LBP_cg2219 | hypothetical protein | 7 | 15 |
| LBP_cg2214 | Uracil phosphoribosyltransferase 1 | 7 | 24 |
| LBP_cg1212 | putative Xaa-Pro dipeptidase | 7 | 17 |
| LBP_cg1922 | XRE family transcriptional regulator | 7 | 25 |
| LBP_cg1119 | ABC transporter, ATP-binding protein | 7 | 15 |
| LBP_cg0532 | O-sialoglycoprotein endopeptidase | 7 | 15 |
| LBP_cg2457 | putative universal stress protein | 7 | 23 |
| LBP_cg2706 | Oxidoreductase | 7 | 19 |
| LBP_cg0356 | hypothetical protein | 7 | 18 |
| LBP_p3g034 | hypothetical protein | 7 | 19 |
| LBP_cg2742 | Bile salt hydrolase | 7 | 17 |
| LBP_cg2919 | D-ribose pyranase | 7 | 20 |
| LBP_cg2351 | Phosphoglycerate mutase | 7 | 14 |
| LBP_cg0481 | Extracellular zinc metalloproteinase | 7 | 19 |
| LBP_cg1895 | Glutamine ABC transporter, ATP-binding protein | 7 | 17 |
| LBP_cg0215 | Nucleoside-diphosphate kinase | 7 | 25 |
| LBP_cg1177 | rRNA methylase | 7 | 12 |
| LBP_cg1199 | Glutamine synthetase repressor | 7 | 20 |
| LBP_cg0872 | Cation transport protein | 7 | 21 |
| LBP_cg0389 | Hydrolase, HAD superfamily, Cof family | 7 | 13 |
| LBP_cg1836 | Adenine-specific methyltransferase | 7 | 16 |
| LBP_cg2355 | Alcohol dehydrogenase | 7 | 14 |
| LBP_cg0846 | Cobalt import ATP-binding protein CbiO 2 | 7 | 14 |
| LBP_cg1404 | hypothetical protein | 7 | 18 |
| LBP_cg1898 | Septum site-determining protein MinC | 7 | 20 |
| LBP_cg1552 | Phosphate starvation-inducible protein | 7 | 15 |
| LBP_cg1598 | Ribosome-binding factor A | 7 | 23 |
| LBP_cg0514 | Recombination protein recR | 7 | 17 |
| LBP_cg0431 | Peptidyl-tRNA hydrolase | 7 | 15 |
| LBP_cg1234 | Serine/threonine specific protein phosphatase (Putative) | 7 | 14 |
| LBP_cg1139 | Formamidopyrimidine-DNA glycosylase | 7 | 11 |
| LBP_cg2127 | Ferredoxin--NADP reductase | 7 | 14 |
| LBP_cg0968 | Transcription regulator | 7 | 11 |
| LBP_cg2177 | Transcription regulator | 7 | 10 |
| LBP_cg0953 | Gluconokinase | 7 | 9 |
| LBP_cg0122 | Oxidoreductase | 7 | 12 |
| LBP_cg1032 | Response regulator | 7 | 10 |
| LBP_cg0933 | UDP-N-acetylglucosamine 2-epimerase | 7 | 10 |
| LBP_cg2091 | ABC transporter, substrate binding protein | 7 | 9 |
| LBP_cg1215 | N utilization substance protein B | 7 | 18 |
| LBP_cg2599 | Integral membrane protein | 7 | 16 |
| LBP_cg0004 | DNA replication and repair protein recF | 7 | 11 |
| LBP_cg0893 | Cytochrome D ABC transporter, ATP-binding and permease protein | 7 | 14 |
| LBP_cg1784 | Cell division protein (Putative) | 7 | 12 |
| LBP_cg2497 | Aryl-alcohol dehydrogenase | 7 | 9 |
| LBP_cg0616 | hypothetical protein | 7 | 11 |
| LBP_cg0355 | Hydroxymethylglutaryl-CoA reductase | 7 | 10 |
| LBP_cg0015 | Glutamate 5-kinase | 7 | 10 |
| LBP_cg1220 | Hemolysin-like protein | 7 | 10 |
| LBP_cg1863 | hypothetical protein | 7 | 14 |
| LBP_cg0358 | hypothetical protein | 7 | 10 |
| LBP_cg1608 | Zinc-dependent protease, membrane associated (Putative) | 7 | 11 |
| LBP_cg0460 | S1 domain RNA-binding protein | 7 | 10 |
| LBP_cg2594 | Sensor protein | 7 | 7 |
| LBP_cg1173 | Exonuclease SbcD | 7 | 10 |
| LBP_cg2467 | Transcription regulator | 7 | 9 |
| LBP_p6g007 | Replication protein A | 7 | 8 |
| LBP_cg2913 | Sorbitol PTS, EIIBC | 7 | 9 |
| LBP_cg2890 | N-acetylneuraminate lyase | 7 | 8 |
| LBP_cg2334 | ABC transporter, permease protein (Putative) | 7 | 10 |
| LBP_cg0505 | Lysyl-tRNA synthetase (Class II) | 7 | 8 |
| LBP_cg0594 | hypothetical protein | 7 | 9 |
| LBP_cg2544 | GTP-binding protein LepA | 7 | 7 |
| LBP_cg2137 | Dehydrogenase | 7 | 7 |
| LBP_cg1209 | 50S ribosomal protein L21 | 6 | 227 |
| LBP_cg0008 | 30S ribosomal protein S6 | 6 | 92 |
| LBP_cg0537 | 10 kDa chaperonin | 6 | 124 |
| LBP_cg1211 | 50S ribosomal protein L27 | 6 | 117 |
| LBP_cg1855 | 2,3,4,5-tetrahydropyridine-2,6-dicarboxylate N-acetyltransferase | 6 | 70 |
| LBP_cg2276 | Cellobiose PTS, EIIA | 6 | 75 |
| LBP_cg2170 | hypothetical protein | 6 | 46 |
| LBP_cg1145 | Translation initiation factor IF-3 | 6 | 43 |
| LBP_cg1160 | hypothetical protein | 6 | 35 |
| LBP_cg1938 | ATP synthase epsilon chain | 6 | 26 |
| LBP_cg0009 | Single-stranded DNA-binding protein | 6 | 45 |
| LBP_cg1314 | hypothetical protein | 6 | 54 |
| LBP_cg2286 | Oxidoreductase | 6 | 28 |
| LBP_cg1759 | Peptide deformylase | 6 | 28 |
| LBP_cg0925 | Universal stress protein UspA family nucleotide-binding protein | 6 | 28 |
| LBP_cg1732 | 30S ribosomal protein S15 | 6 | 32 |
| LBP_cg2750 | hypothetical protein | 6 | 23 |
| LBP_cg2343 | DNA-(Apurinic or apyrimidinic site) lyase | 6 | 21 |
| LBP_cg1118 | Cell-cycle regulation histidine triad protein | 6 | 27 |
| LBP_cg0607 | ATP-binding protein | 6 | 18 |
| LBP_cg2144 | Pyrazinamidase/nicotinamidase | 6 | 27 |
| LBP_cg0575 | HD superfamily hydrolase | 6 | 19 |
| LBP_cg1285 | Acetyl-CoA carboxylase, biotin carboxyl carrier protein | 6 | 19 |
| LBP_cg1380 | Pyrimidine regulatory protein PyrR | 6 | 28 |
| LBP_cg0519 | Initiation-control protein yabA | 6 | 23 |
| LBP_cg1195 | Rhodanese family protein | 6 | 23 |
| LBP_cg0661 | hypothetical protein | 6 | 17 |
| LBP_cg1812 | Copper homeostasis protein | 6 | 15 |
| LBP_cg0876 | Citrate lyase acyl carrier protein | 6 | 19 |
| LBP_cg2254 | NUDIX family hydrolase | 6 | 16 |
| LBP_cg0428 | CBS domain containing protein | 6 | 14 |
| LBP_cg2766 | Transcription regulator | 6 | 16 |
| LBP_cg1571 | Ribosomal protein L11 methyltransferase | 6 | 14 |
| LBP_cg1155 | HAD superfamily hydrolase | 6 | 15 |
| LBP_cg1416 | Transcription repressor | 6 | 13 |
| LBP_cg1918 | Recombination factor protein RarA | 6 | 12 |
| LBP_cg0918 | Transcription regulator | 6 | 10 |
| LBP_cg0281 | Acetyltransferase | 6 | 14 |
| LBP_cg2133 | Ribonuclease H (Putative) | 6 | 14 |
| LBP_cg1750 | Extracellular protein | 6 | 13 |
| LBP_cg2184 | Small heat shock protein | 6 | 18 |
| LBP_cg2082 | Ring-cleaving dioxygenase (Putative) | 6 | 16 |
| LBP_cg0951 | Glycerol-3-phosphate cytidylyltransferase | 6 | 17 |
| LBP_cg2431 | Transcription regulator | 6 | 13 |
| LBP_p2g004 | Beta-galactosidase | 6 | 10 |
| LBP_cg2288 | Flavodoxin | 6 | 16 |
| LBP_cg0622 | putative sugar-phosphatase | 6 | 11 |
| LBP_cg0024 | HAD superfamily hydrolase | 6 | 12 |
| LBP_cg1803 | rRNA methyltransferase | 6 | 12 |
| LBP_cg1913 | hypothetical protein | 6 | 9 |
| LBP_cg1393 | Transcription regulator | 6 | 14 |
| LBP_cg2335 | Short-chain dehydrogenase/oxidoreductase | 6 | 9 |
| LBP_cg1483 | Protein of hypothetical function DUF34 | 6 | 12 |
| LBP_cg2073 | putative hydrolase | 6 | 10 |
| LBP_cg0520 | Methyltransferase (Putative) | 6 | 9 |
| LBP_cg0484 | Acetyltransferase | 6 | 12 |
| LBP_cg0319 | Acetyl-CoA carboxylase, biotin carboxyl carrier protein | 6 | 16 |
| LBP_cg2419 | Tannase | 6 | 9 |
| LBP_cg1740 | putative rRNA (Guanine-N(2)-)-methyltransferase | 6 | 11 |
| LBP_cg0144 | LacI family transcriptional regulator | 6 | 11 |
| LBP_cg0301 | Teichoic acid ABC transporter, permease protein | 6 | 15 |
| LBP_cg0396 | Serine transporter | 6 | 10 |
| LBP_cg2369 | Oxidoreductase | 6 | 13 |
| LBP_cg1890 | Zinc-dependent proteinase | 6 | 11 |
| LBP_cg2756 | hypothetical protein | 6 | 14 |
| LBP_cg2718 | Adenine deaminase | 6 | 10 |
| LBP_cg1038 | hypothetical protein | 6 | 12 |
| LBP_cg0556 | Phosphate import ATP-binding protein pstB 2 | 6 | 10 |
| LBP_cg1037 | hypothetical protein | 6 | 11 |
| LBP_cg0503 | Membrane nuclease | 6 | 7 |
| LBP_cg1894 | Glutamine ABC superfamily ATP binding cassette transporter, substrate binding protein | 6 | 10 |
| LBP_p2g025 | hypothetical protein | 6 | 11 |
| LBP_cg2798 | Transcription regulator | 6 | 7 |
| LBP_cg1165 | Lipoprotein | 6 | 10 |
| LBP_cg2605 | Ribosomal RNA small subunit methyltransferase G | 6 | 7 |
| LBP_cg0395 | Seryl-tRNA synthetase 1 | 6 | 12 |
| LBP_cg1423 | hypothetical protein | 6 | 8 |
| LBP_cg2692 | GTP cyclohydrolase 1 | 6 | 12 |
| LBP_cg0624 | Aldose 1-epimerase | 6 | 8 |
| LBP_cg2085 | 2-dehydropantoate 2-reductase | 6 | 10 |
| LBP_p2g005 | Beta-galactosidase large subunit | 6 | 9 |
| LBP_cg2228 | Amidophosphoribosyltransferase | 6 | 8 |
| LBP_cg0485 | Trimetaphosphatase | 6 | 10 |
| LBP_cg0405 | Sortase | 6 | 9 |
| LBP_cg1412 | D-ribitol-5-phosphate cytidylyltransferase | 6 | 9 |
| LBP_cg2629 | hypothetical protein | 6 | 10 |
| LBP_cg2627 | ABC superfamily ATP binding cassette transporter, ABC protein | 6 | 9 |
| LBP_cg2812 | hypothetical protein | 6 | 7 |
| LBP_cg0609 | DNA-directed DNA polymerase III, epsilon chain | 6 | 7 |
| LBP_cg1603 | Ribosome maturation factor rimP | 6 | 11 |
| LBP_cg2879 | Transcription regulator | 6 | 8 |
| LBP_cg0510 | Methyltransferase | 6 | 9 |
| LBP_cg1671 | Phage replication initiation protein | 6 | 10 |
| LBP_cg1230 | Pantothenate metabolism flavoprotein Dfp | 6 | 7 |
| LBP_p5g012 | hypothetical protein | 6 | 7 |
| LBP_cg2314 | Dihydrofolate reductase | 6 | 7 |
| LBP_cg0390 | Hydrolase (Putative) | 6 | 8 |
| LBP_cg1739 | Phosphopantetheine adenylyltransferase | 6 | 10 |
| LBP_cg1132 | Cysteine desulfurase | 6 | 8 |
| LBP_cg2093 | ABC superfamily ATP binding cassette transporter, ABC protein | 6 | 7 |
| LBP_cg2843 | Transcription regulator | 6 | 8 |
| LBP_cg1728 | TPR repeat-containing protein | 6 | 6 |
| LBP_cg0848 | tRNA pseudouridine synthase A | 6 | 7 |
| LBP_cg1878 | Integral membrane protein | 6 | 6 |
| LBP_cg0835 | 30S ribosomal protein S11 | 5 | 85 |
| LBP_cg0719 | hypothetical protein | 5 | 34 |
| LBP_cg1931 | Glycine cleavage system, H protein | 5 | 49 |
| LBP_cg0833 | Translation initiation factor IF-1 | 5 | 44 |
| LBP_cg1852 | Methyl-accepting chemotaxis family protein | 5 | 29 |
| LBP_cg0217 | putative NAD(P)H dehydrogenase (Quinone) | 5 | 23 |
| LBP_cg2285 | Phosphoglycerate dehydrogenase | 5 | 20 |
| LBP_p5g014 | hypothetical protein | 5 | 23 |
| LBP_cg0819 | 50S ribosomal protein L29 | 5 | 32 |
| LBP_cg2644 | hypothetical protein | 5 | 30 |
| LBP_cg2928 | Universal stress protein UspA family protein | 5 | 25 |
| LBP_cg0466 | Mannose PTS, EIIC | 5 | 23 |
| LBP_cg0798 | Deoxyguanosine kinase | 5 | 21 |
| LBP_cg0077 | hypothetical protein | 5 | 23 |
| LBP_cg0280 | hypothetical protein | 5 | 16 |
| LBP_cg1117 | hypothetical protein | 5 | 13 |
| LBP_p2g039 | hypothetical protein | 5 | 17 |
| LBP_cg1253 | Ribosome maturation factor rimM | 5 | 16 |
| LBP_cg1236 | Translation factor, GTPase (Putative) | 5 | 15 |
| LBP_cg1821 | Beta-lactamase superfamily hydrolase | 5 | 12 |
| LBP_cg2015 | Transcriptional regulator | 5 | 12 |
| LBP_cg2878 | Galacitol PTS, EIIA | 5 | 11 |
| LBP_cg2241 | L-asparaginase | 5 | 12 |
| LBP_cg1240 | 50S ribosomal protein L28 | 5 | 30 |
| LBP_cg0312 | Ribose 5-phosphate epimerase | 5 | 11 |
| LBP_cg1914 | hypothetical protein | 5 | 11 |
| LBP_cg2231 | Purine biosynthesis cluster protein | 5 | 16 |
| LBP_cg2262 | ADP-ribose pyrophosphatase | 5 | 14 |
| LBP_cg1936 | Ribosomal protein acetylating enzyme | 5 | 11 |
| LBP_cg0801 | Transcription regulator | 5 | 10 |
| LBP_cg0278 | Spermidine/putrescine import ATP-binding protein PotA | 5 | 13 |
| LBP_cg2946 | hypothetical protein | 5 | 10 |
| LBP_cg1734 | DNA-directed DNA polymerase III, delta chain | 5 | 8 |
| LBP_cg0282 | Acetyltransferase | 5 | 10 |
| LBP_cg0511 | Cytosine/adenosine deaminase | 5 | 9 |
| LBP_cg0480 | Phosphoglycerate mutase | 5 | 10 |
| LBP_cg1524 | Transcription regulator | 5 | 7 |
| LBP_cg0724 | 2-hydroxyhepta-2,4-diene-1,7-dioateisomerase / 5-carboxymethyl-2-oxo-hex-3-ene-1,7-dioatedecarboxylase (Putative) | 5 | 7 |
| LBP_cg1331 | Major facilitator superfamily permease | 5 | 17 |
| LBP_cg1007 | MOP superfamily multidrug/oligosaccharidyl-lipid/polysaccharide flippase transporter | 5 | 9 |
| LBP_cg1768 | TPR repeat-containing protein | 5 | 10 |
| LBP_cg0947 | Transcription regulator | 5 | 13 |
| LBP_cg0767 | Alpha-ribazole-5'-phosphate phosphatase (Putative) | 5 | 11 |
| LBP_cg2840 | 3-dehydroquinate dehydratase | 5 | 8 |
| LBP_cg1780 | ADP-ribose pyrophosphatase | 5 | 8 |
| LBP_cg1601 | hypothetical protein | 5 | 15 |
| LBP_cg1238 | hypothetical protein | 5 | 9 |
| LBP_cg0118 | hypothetical protein | 5 | 13 |
| LBP_cg1933 | hypothetical protein | 5 | 11 |
| LBP_cg0386 | Lipoprotein | 5 | 9 |
| LBP_p6g002 | hypothetical protein | 5 | 10 |
| LBP_cg0172 | XRE family transcriptional regulator | 5 | 9 |
| LBP_cg2282 | HAD superfamily hydrolase | 5 | 8 |
| LBP_cg0694 | 2-nitropropane dioxygenase | 5 | 10 |
| LBP_cg2732 | hypothetical protein | 5 | 13 |
| LBP_cg0530 | Ribosomal-protein-alanine N-acetyltransferase | 5 | 10 |
| LBP_cg1875 | Holliday junction DNA helicase RuvA | 5 | 8 |
| LBP_cg0614 | Transcription regulator | 5 | 8 |
| LBP_cg2891 | Putative N-acetylmannosamine-6-phosphate 2-epimerase | 5 | 7 |
| LBP_cg0613 | Na(+)/H(+) antiporter (Putative) | 5 | 8 |
| LBP_cg2754 | BS_ykrK family protein | 5 | 7 |
| LBP_cg0552 | Phosphate ABC superfamily ATP binding cassette transporter, substrate binding protein | 5 | 6 |
| LBP_cg0025 | Beta-phosphoglucomutase | 5 | 9 |
| LBP_cg1488 | Transcription regulator | 5 | 7 |
| LBP_cg0627 | Nitro/flavin reductase | 5 | 12 |
| LBP_cg0803 | Transcription regulator | 5 | 8 |
| LBP_cg0476 | Acetyl-CoA carboxylase, carboxyl transferase subunit alpha | 5 | 8 |
| LBP_cg1170 | Sensor protein | 5 | 7 |
| LBP_cg0573 | D-alanyl-D-alanine dipeptidase | 5 | 8 |
| LBP_cg0491 | Ribonuclease III | 5 | 8 |
| LBP_cg1814 | putative inorganic polyphosphate/ATP-NAD kinase | 5 | 9 |
| LBP_cg2434 | Nitroreductase | 5 | 8 |
| LBP_cg2554 | 6-phospho-beta-glucosidase | 5 | 8 |
| LBP_cg1555 | Ribosomal protein S21 | 5 | 13 |
| LBP_cg1168 | Recombination factor protein RarA | 5 | 7 |
| LBP_cg0371 | 4-diphosphocytidyl-2-C-methyl-D-erythritol kinase | 5 | 7 |
| LBP_cg1804 | putative FMN reductase | 5 | 11 |
| LBP_cg2709 | hypothetical protein | 5 | 12 |
| LBP_p1g033 | Copy number control protein | 5 | 8 |
| LBP_cg2146 | ABC transporter, ATP-binding protein | 5 | 6 |
| LBP_p2g029 | Potassium uptake protein | 5 | 11 |
| LBP_cg1627 | Glycosyltransferase | 5 | 9 |
| LBP_cg0354 | Exopolyphosphatase | 5 | 6 |
| LBP_cg1888 | CDP-diacylglycerol--glycerol-3-phosphate 3-phosphatidyltransferase | 5 | 9 |
| LBP_cg1769 | Phosphoglycerate mutase | 5 | 5 |
| LBP_cg0950 | Transport protein | 5 | 7 |
| LBP_cg1046 | 5-methyltetrahydropteroyltriglutamate--homocysteine methyltransferase | 5 | 5 |
| LBP_cg2664 | Cell surface hydrolase, membrane-bound (Putative) | 5 | 6 |
| LBP_cg2780 | Glutamate decarboxylase | 5 | 6 |
| LBP_cg2180 | Hydrolase | 5 | 6 |
| LBP_cg2268 | Transcriptional regulator | 5 | 7 |
| LBP_cg0929 | hypothetical protein | 5 | 8 |
| LBP_cg2080 | ABC superfamily ATP binding cassette transporter, ABC protein | 5 | 6 |
| LBP_cg0555 | Phosphate import ATP-binding protein pstB 1 | 5 | 5 |
| LBP_cg0698 | hypothetical protein | 5 | 6 |
| LBP_cg0549 | Integral membrane protein | 5 | 7 |
| LBP_cg1333 | Aldose 1-epimerase | 5 | 8 |
| LBP_cg1074 | Bifunctional GntR family transcriptional regulator/aminotransferase | 5 | 8 |
| LBP_cg0243 | Sensor protein | 5 | 5 |
| LBP_cg1175 | Membrane protein oxaA 2 | 5 | 8 |
| LBP_cg2289 | Cell surface protein | 5 | 5 |
| LBP_cg2842 | Shikimate 5-dehydrogenase | 5 | 7 |
| LBP_cg1545 | DNA primase DnaG | 5 | 6 |
| LBP_cg0408 | hypothetical protein | 5 | 6 |
| LBP_cg1856 | Cystathionine beta-synthase (CBS) domain protein | 5 | 5 |
| LBP_cg1456 | CCA-adding enzyme | 5 | 6 |
| LBP_p5g013 | Tyrosine recombinase | 5 | 5 |
| LBP_cg0244 | Response regulator | 5 | 5 |
| LBP_p5g011 | Transcriptional regulator | 5 | 8 |
| LBP_cg1294 | Acyl-CoA thioester hydrolase (Putative) | 5 | 5 |
| LBP_cg0650 | X-prolyl-dipeptidyl aminopeptidase | 5 | 5 |
| LBP_cg0691 | 7,8-dihydro-8-oxoguanine-triphosphatase | 5 | 5 |
| LBP_cg0973 | Phosphocarrier protein HPr | 4 | 395 |
| LBP_cg2450 | hypothetical protein | 4 | 159 |
| LBP_cg1761 | hypothetical protein | 4 | 81 |
| LBP_cg0497 | Transcription antitermination protein nusG | 4 | 68 |
| LBP_cg0403 | 50S ribosomal protein L31 type B | 4 | 54 |
| LBP_cg1871 | Preprotein translocase, subunit | 4 | 55 |
| LBP_cg0820 | 30S ribosomal protein S17 | 4 | 55 |
| LBP_cg0818 | 50S ribosomal protein L16 | 4 | 49 |
| LBP_cg1017 | Large-conductance mechanosensitive channel | 4 | 24 |
| LBP_cg1245 | Acyl carrier protein | 4 | 24 |
| LBP_cg1229 | DNA-directed RNA polymerase subunit omega | 4 | 24 |
| LBP_cg1866 | hypothetical protein | 4 | 21 |
| LBP_cg1911 | Signal protein | 4 | 13 |
| LBP_p1g007 | Cupin 2 conserved barrel domain protein | 4 | 19 |
| LBP_cg0921 | Cold shock protein 1 | 4 | 26 |
| LBP_cg2281 | hypothetical protein | 4 | 11 |
| LBP_cg0928 | hypothetical protein | 4 | 12 |
| LBP_cg2730 | hypothetical protein | 4 | 12 |
| LBP_cg2271 | ABC superfamily ATP binding cassette transporter, permease protein | 4 | 9 |
| LBP_cg1957 | Cobyric acid synthase (Putative) | 4 | 11 |
| LBP_cg0196 | Glutathione peroxidase | 4 | 11 |
| LBP_cg0470 | Mannose PTS, EIIA | 4 | 10 |
| LBP_cg2744 | Penicillinase repressor | 4 | 8 |
| LBP_cg2163 | hypothetical protein | 4 | 12 |
| LBP_cg1187 | hypothetical protein | 4 | 15 |
| LBP_cg2809 | Transcription regulator | 4 | 12 |
| LBP_cg1692 | hypothetical protein | 4 | 10 |
| LBP_cg1036 | Transcription regulator | 4 | 10 |
| LBP_cg2255 | hypothetical protein | 4 | 12 |
| LBP_cg0851 | putative spore protein YitS | 4 | 9 |
| LBP_cg1218 | Exodeoxyribonuclease 7 small subunit | 4 | 13 |
| LBP_cg1318 | putative tautomerase | 4 | 11 |
| LBP_cg0373 | Family S9 peptidase | 4 | 8 |
| LBP_cg0539 | Transport protein | 4 | 9 |
| LBP_cg0412 | Holo-acyl-carrier-protein | 4 | 10 |
| LBP_cg1077 | Arginine regulator | 4 | 10 |
| LBP_cg1874 | Holliday junction ATP-dependent DNA helicase ruvB | 4 | 8 |
| LBP_cg1252 | RNA-binding protein | 4 | 8 |
| LBP_p3g036 | hypothetical protein | 4 | 8 |
| LBP_cg2333 | ABC transporter, ATP-binding protein | 4 | 7 |
| LBP_cg0158 | Fructokinase | 4 | 11 |
| LBP_cg1620 | hypothetical protein | 4 | 17 |
| LBP_cg1551 | Putative metalloprotease | 4 | 10 |
| LBP_cg1860 | hypothetical protein | 4 | 9 |
| LBP_cg0036 | Ribosomal RNA large subunit methyltransferase H | 4 | 10 |
| LBP_cg2182 | BS_ykrK family protein | 4 | 7 |
| LBP_cg0959 | hypothetical protein | 4 | 5 |
| LBP_cg1133 | NifU-like protein | 4 | 8 |
| LBP_cg1337 | Mevalonate kinase | 4 | 7 |
| LBP_cg1694 | hypothetical protein | 4 | 6 |
| LBP_cg1891 | M16 family metallopeptidase | 4 | 9 |
| LBP_cg0102 | hypothetical protein | 4 | 7 |
| LBP_cg1857 | Phosphoesterase (Putative) | 4 | 10 |
| LBP_cg2612 | putative Aromatic-amino-acid transaminase | 4 | 6 |
| LBP_cg0875 | (Citrate (Pro-3S)-lyase) ligase | 4 | 6 |
| LBP_cg1570 | Protein of hypothetical function DUF558 | 4 | 7 |
| LBP_cg1449 | hypothetical protein | 4 | 6 |
| LBP_cg0092 | Oxidoreductase | 4 | 7 |
| LBP_cg1243 | ATP-dependent DNA helicase RecG | 4 | 5 |
| LBP_cg1698 | Short chain dehydrogenase | 4 | 4 |
| LBP_cg0459 | hypothetical protein | 4 | 8 |
| LBP_cg2611 | Transcription regulator | 4 | 7 |
| LBP_cg0275 | Spermidine/putrescine ABC transporter, substrate binding protein | 4 | 7 |
| LBP_cg1505 | hypothetical protein | 4 | 7 |
| LBP_cg2846 | Acetylesterase | 4 | 7 |
| LBP_cg0599 | SsrA-binding protein | 4 | 10 |
| LBP_p3g011 | Peptidoglycan-binding protein | 4 | 7 |
| LBP_cg2719 | Glutamine--fructose-6-phosphate transaminase (Isomerizing) | 4 | 6 |
| LBP_cg0294 | hypothetical protein | 4 | 8 |
| LBP_cg1334 | Isopentenyl pyrophosphate isomerase | 4 | 7 |
| LBP_cg0178 | Phosphoglycerate mutase (Putative) | 4 | 7 |
| LBP_cg0504 | Prophage Lp1 protein 66, lipoprotein | 4 | 7 |
| LBP_cg0633 | Negative regulator of proteolysis | 4 | 7 |
| LBP_cg0845 | Cobalt import ATP-binding protein CbiO 1 | 4 | 7 |
| LBP_cg2456 | Manganese transport protein | 4 | 9 |
| LBP_cg1786 | Cell division protein sepF | 4 | 9 |
| LBP_cg1216 | Methenyltetrahydrofolate cyclohydrolase | 4 | 5 |
| LBP_cg0493 | hypothetical protein | 4 | 4 |
| LBP_cg2296 | Oxidoreductase | 4 | 6 |
| LBP_cg0658 | Guanylate kinase | 4 | 6 |
| LBP_cg1752 | Myo-inositol-1(Or 4)-monophosphatase | 4 | 5 |
| LBP_cg0945 | hypothetical protein | 4 | 5 |
| LBP_cg0965 | Oligopeptide ABC transporter, permease protein | 4 | 7 |
| LBP_cg2074 | Transcription regulator, AsnC-type | 4 | 6 |
| LBP_cg2881 | Transcription regulator | 4 | 6 |
| LBP_cg0397 | L-serine dehydratase, beta subunit | 4 | 7 |
| LBP_cg2422 | putative acyltransferase | 4 | 6 |
| LBP_cg2240 | Cell surface hydrolase, membrane-bound | 4 | 7 |
| LBP_cg2181 | Response regulator | 4 | 5 |
| LBP_cg2330 | Extracellular protein | 4 | 6 |
| LBP_cg2226 | Phosphoribosylglycinamide formyltransferase | 4 | 6 |
| LBP_cg2856 | 6-phospho-beta-glucosidase | 4 | 8 |
| LBP_p6g003 | hypothetical protein | 4 | 6 |
| LBP_cg0831 | Preprotein translocase subunit secY | 4 | 7 |
| LBP_cg1465 | hypothetical protein | 4 | 7 |
| LBP_cg0944 | Recombination regulator RecX | 4 | 6 |
| LBP_cg1932 | Bacterial cell division membrane protein FtsW | 4 | 6 |
| LBP_cg0207 | Transcription regulator, mannitol operon | 4 | 4 |
| LBP_cg2854 | Transcription antiterminator | 4 | 5 |
| LBP_cg0689 | Phosphoglycerate mutase | 4 | 7 |
| LBP_cg2836 | Aldo/keto reductase family protein | 4 | 4 |
| LBP_cg0272 | Transcription regulator | 4 | 6 |
| LBP_cg1847 | hypothetical protein | 4 | 5 |
| LBP_cg1472 | putative reductase | 4 | 5 |
| LBP_cg1591 | tRNA pseudouridine synthase B | 4 | 5 |
| LBP_cg2921 | Ribose operon repressor | 4 | 4 |
| LBP_cg1188 | Integral membrane protein | 4 | 7 |
| LBP_cg1088 | Fumarate reductase, flavoprotein subunit | 4 | 4 |
| LBP_cg1575 | hypothetical protein | 4 | 5 |
| LBP_cg1379 | Pseudouridine synthase | 4 | 4 |
| LBP_cg1502 | Transcription regulator | 4 | 4 |
| LBP_cg0127 | Transcription regulator | 4 | 4 |
| LBP_cg2502 | Transcription regulator | 4 | 5 |
| LBP_cg1470 | Segregation and condensation protein B | 4 | 4 |
| LBP_cg0344 | Cyanide hydratase | 4 | 5 |
| LBP_cg1319 | Diaminopimelate decarboxylase | 4 | 4 |
| LBP_cg0946 | Extracellular protein, gamma-D-glutamate-meso-diaminopimelate muropeptidase (Putative) | 4 | 4 |
| LBP_cg1426 | Peptide methionine sulfoxide reductase msrB | 4 | 4 |
| LBP_cg1507 | hypothetical protein | 4 | 4 |
| LBP_cg1831 | hypothetical protein | 4 | 5 |
| LBP_cg2929 | Regulator of phenolic acid metabolism PadR | 4 | 4 |
| LBP_cg0124 | ABC superfamily ATP binding cassette transporter, ABC protein | 4 | 4 |
| LBP_cg2533 | Short-chain dehydrogenase/oxidoreductase | 4 | 4 |
| LBP_cg0838 | Trans-hexaprenyltranstransferase, component II | 4 | 5 |
| LBP_cg1478 | DNA-directed DNA polymerase III, alpha chain | 4 | 4 |
| LBP_cg1249 | hypothetical protein | 4 | 4 |
| LBP_cg0782 | putative transcriptional regulator | 4 | 5 |
| LBP_cg2339 | Succinyl-diaminopimelate desuccinylase | 4 | 5 |
| LBP_cg1197 | Glycerophosphodiester phosphodiesterase | 4 | 4 |
| LBP_cg0954 | 6-phosphogluconate dehydrogenase, decarboxylating | 4 | 4 |
| LBP_cg1281 | Acyl carrier protein | 3 | 160 |
| LBP_cg1251 | 30S ribosomal protein S16 | 3 | 25 |
| LBP_cg0829 | 50S ribosomal protein L30 | 3 | 39 |
| LBP_cg1214 | Alkaline shock protein | 3 | 24 |
| LBP_cg0822 | 50S ribosomal protein L24 | 3 | 23 |
| LBP_cg0634 | hypothetical protein | 3 | 14 |
| LBP_cg0208 | Mannitol PTS, EIIA | 3 | 12 |
| LBP_cg1224 | Glycine betaine/carnitine/choline ABC transporter, permease protein | 3 | 13 |
| LBP_cg1589 | Alpha-acetolactate decarboxylase | 3 | 15 |
| LBP_cg2061 | ACT domain protein | 3 | 22 |
| LBP_cg0392 | hypothetical protein | 3 | 17 |
| LBP_cg1810 | Glucitol/sorbitol PTS, EIIA | 3 | 10 |
| LBP_p2g047 | hypothetical protein | 3 | 12 |
| LBP_cg1697 | hypothetical protein | 3 | 13 |
| LBP_cg1157 | RNA-binding protein | 3 | 10 |
| LBP_cg0671 | Glutamine ABC transporter, permease protein | 3 | 14 |
| LBP_cg2132 | Purine nucleosidase | 3 | 11 |
| LBP_cg1226 | Glycine betaine/carnitine/choline ABC transporter, ATP-binding protein | 3 | 6 |
| LBP_cg0452 | Pyrroline-5-carboxylate reductase | 3 | 11 |
| LBP_cg0048 | hypothetical protein | 3 | 10 |
| LBP_cg0434 | S4 RNA-binding domain protein | 3 | 12 |
| LBP_cg2160 | Thioredoxin H-type | 3 | 12 |
| LBP_cg2782 | hypothetical protein | 3 | 6 |
| LBP_cg2745 | hypothetical protein | 3 | 7 |
| LBP_cg2646 | hypothetical protein | 3 | 8 |
| LBP_cg2936 | Transcription regulator | 3 | 11 |
| LBP_cg2152 | Transcription regulator | 3 | 6 |
| LBP_cg1176 | Acylphosphatase | 3 | 11 |
| LBP_cg2725 | Phosphatidylglycerophosphatase | 3 | 10 |
| LBP_cg0475 | Acetyl-coenzyme A carboxylase carboxyl transferase subunit beta 1 | 3 | 10 |
| LBP_cg1621 | Protein of hypothetical function DUF896 | 3 | 10 |
| LBP_cg0891 | Cytochrome D ubiquinol oxidase, subunit I | 3 | 6 |
| LBP_cg2471 | Cellobiose PTS, EIIC | 3 | 7 |
| LBP_cg2413 | Integral membrane protein | 3 | 5 |
| LBP_cg2837 | FMN-binding protein | 3 | 10 |
| LBP_p1g018 | Manganese transport protein | 3 | 7 |
| LBP_cg2735 | hypothetical protein | 3 | 7 |
| LBP_cg2648 | Ferric uptake regulator | 3 | 9 |
| LBP_cg1141 | Transcriptional repressor nrdR | 3 | 11 |
| LBP_cg0357 | NAD-dependent deacetylase (Regulatory protein SIR2 family protein) | 3 | 6 |
| LBP_cg1431 | Acyl-phosphate glycerol-3-phosphate acyltransferase | 3 | 5 |
| LBP_cg2514 | hypothetical protein | 3 | 8 |
| LBP_cg1962 | hypothetical protein | 3 | 8 |
| LBP_cg1353 | hypothetical protein | 3 | 6 |
| LBP_cg0558 | Stress-responsive transcription regulator (Putative) | 3 | 7 |
| LBP_cg1183 | Uridine kinase | 3 | 8 |
| LBP_cg2234 | Phosphoribosylaminoimidazole carboxylase, catalytic subunit | 3 | 11 |
| LBP_cg0847 | ABC transporter, permease protein | 3 | 7 |
| LBP_cg0517 | hypothetical protein | 3 | 7 |
| LBP_cg2802 | hypothetical protein | 3 | 5 |
| LBP_cg2058 | Sensor protein | 3 | 8 |
| LBP_cg0235 | Transcription regulator | 3 | 5 |
| LBP_cg1241 | hypothetical protein | 3 | 8 |
| LBP_cg0566 | NADH oxidase | 3 | 4 |
| LBP_cg1853 | Mechanosensitive transport protein | 3 | 6 |
| LBP_cg0611 | Dehydrogenase | 3 | 6 |
| LBP_cg0960 | Transcription regulator | 3 | 5 |
| LBP_cg2303 | hypothetical protein | 3 | 6 |
| LBP_cg0897 | Trans-hexaprenyltranstransferase, component II | 3 | 3 |
| LBP_cg1796 | Protein mraZ | 3 | 4 |
| LBP_cg1306 | hypothetical protein | 3 | 6 |
| LBP_cg0445 | putative phosphohydrolase | 3 | 4 |
| LBP_cg1339 | hypothetical protein | 3 | 5 |
| LBP_cg1673 | hypothetical protein | 3 | 7 |
| LBP_cg2597 | Peptidylprolyl isomerase | 3 | 4 |
| LBP_cg1573 | hypothetical protein | 3 | 9 |
| LBP_cg1310 | Diacylglycerol kinase | 3 | 6 |
| LBP_cg2377 | Transcription regulator | 3 | 6 |
| LBP_cg2356 | Short chain dehydrogenase | 3 | 5 |
| LBP_cg0631 | Glycerol kinase 2 | 3 | 6 |
| LBP_cg1564 | N-acetylmuramoyl-L-alanine amidase | 3 | 4 |
| LBP_cg2148 | hypothetical protein | 3 | 6 |
| LBP_cg1203 | hypothetical protein | 3 | 5 |
| LBP_cg0285 | ABC transporter, ATP-binding protein | 3 | 3 |
| LBP_cg1029 | HAD superfamily hydrolase | 3 | 4 |
| LBP_p2g030 | Transcription regulator | 3 | 4 |
| LBP_cg0407 | putative protease htpX | 3 | 5 |
| LBP_cg1618 | Methyltransferase (Putative) | 3 | 6 |
| LBP_cg2793 | Thioredoxin | 3 | 6 |
| LBP_cg2620 | hypothetical protein | 3 | 3 |
| LBP_cg2341 | ABC superfamily ATP binding cassette transporter, ABC protein | 3 | 4 |
| LBP_cg2438 | ATP-binding cassette transporter | 3 | 4 |
| LBP_cg0150 | Maltose/maltodextrin ABC transporter, permease protein | 3 | 5 |
| LBP_cg1140 | Dephospho-CoA kinase | 3 | 4 |
| LBP_cg2246 | ABC superfamily ATP binding cassette transporter, ABC protein | 3 | 3 |
| LBP_cg1652 | HK97 family phage major capsid protein | 3 | 3 |
| LBP_cg1497 | Enolase | 3 | 6 |
| LBP_cg2521 | Transcription regulator | 3 | 5 |
| LBP_cg2903 | Sensor histidine protein kinase | 3 | 5 |
| LBP_cg2403 | Peptide deformylase | 3 | 5 |
| LBP_cg2161 | Cystathionine gamma-synthase | 3 | 4 |
| LBP_cg0161 | Beta-fructofuranosidase | 3 | 3 |
| LBP_cg2851 | Outer surface protein | 3 | 4 |
| LBP_cg2901 | Negative regulator of proteolysis | 3 | 3 |
| LBP_cg0763 | hypothetical protein | 3 | 5 |
| LBP_p5g002 | putative cell surface protein | 3 | 4 |
| LBP_cg1330 | hypothetical protein | 3 | 4 |
| LBP_cg0764 | Integral membrane protein | 3 | 3 |
| LBP_cg2390 | Type I phosphodiesterase/nucleotide pyrophosphatase | 3 | 3 |
| LBP_cg2324 | Ribosomal protein acetylating enzyme | 3 | 4 |
| LBP_cg1773 | Potassium uptake protein | 3 | 4 |
| LBP_cg1580 | D-alanine--poly(phosphoribitol) ligase subunit 2-1 | 3 | 9 |
| LBP_cg0584 | Putative sporulation transcription regulator whiA | 3 | 3 |
| LBP_cg2662 | Dehydrosqualene synthase | 3 | 4 |
| LBP_cg2952 | Ribonuclease P protein component | 3 | 6 |
| LBP_cg0990 | Ferrochelatase | 3 | 3 |
| LBP_cg2404 | Thiamin biosynthesis lipoprotein ApbE | 3 | 3 |
| LBP_cg1162 | hypothetical protein | 3 | 3 |
| LBP_cg0368 | Ribonuclease M5 | 3 | 5 |
| LBP_cg2495 | Extracellular protein | 3 | 4 |
| LBP_cg2758 | 2',3'-cyclic-nucleotide 2'-phosphodiesterase, putative | 3 | 5 |
| LBP_cg2635 | Protein-N(Pi)-phosphohistidine--sugar phosphotransferase | 3 | 3 |
| LBP_cg0610 | Exodeoxyribonuclease III | 3 | 4 |
| LBP_cg2336 | 4-carboxymuconolactone decarboxylase (Putative) | 3 | 3 |
| LBP_cg1471 | Segregation and condensation protein | 3 | 3 |
| LBP_cg2278 | Transcription regulator | 3 | 4 |
| LBP_cg0543 | hypothetical protein | 3 | 5 |
| LBP_cg2150 | Cell surface hydrolase, membrane-bound (Putative) | 3 | 4 |
| LBP_p3g026 | hypothetical protein | 3 | 5 |
| LBP_cg0640 | Lipopolysaccharide biosynthesis protein LicD | 3 | 3 |
| LBP_cg2661 | Squalene synthase | 3 | 3 |
| LBP_cg0506 | Lysine decarboxylase | 3 | 5 |
| LBP_cg1207 | hypothetical protein | 3 | 6 |
| LBP_cg1666 | Modification methylase Rho11sI family protein | 3 | 4 |
| LBP_cg0760 | Pseudouridine synthase | 3 | 4 |
| LBP_cg2553 | Transcription regulator | 3 | 3 |
| LBP_cg0791 | Acetyltransferase | 3 | 3 |
| LBP_cg0621 | Diguanylate cyclase/phosphodiesterase domain-containing protein | 3 | 4 |
| LBP_cg2788 | Menaquinone biosynthesis methyltransferase ubiE | 3 | 4 |
| LBP_cg2245 | Transcription regulator | 3 | 4 |
| LBP_cg0279 | Transcription regulator | 3 | 6 |
| LBP_cg0927 | Cell surface hydrolase | 3 | 3 |
| LBP_cg0028 | Cold shock protein 2 | 3 | 7 |
| LBP_cg1903 | Folylpolyglutamate synthase | 3 | 3 |
| LBP_p1g016 | Resolvase | 3 | 4 |
| LBP_cg2379 | Acetyltransferase | 3 | 5 |
| LBP_cg1435 | Integrase/recombinase | 3 | 3 |
| LBP_cg2525 | Response regulator | 3 | 4 |
| LBP_cg2472 | 6-phospho-beta-glucosidase | 3 | 3 |
| LBP_cg2465 | Ser/Thr protein phosphatase family protein | 3 | 3 |
| LBP_cg2257 | Neopullulanase | 3 | 3 |
| LBP_cg1677 | hypothetical protein | 3 | 5 |
| LBP_cg1609 | Phosphatidate cytidylyltransferase | 3 | 4 |
| LBP_cg2861 | Sugar transport protein | 3 | 4 |
| LBP_cg1336 | Diphosphomevalonate decarboxylase | 3 | 3 |
| LBP_cg1057 | hypothetical protein | 3 | 4 |
| LBP_cg1490 | hypothetical protein | 3 | 5 |
| LBP_cg1425 | Protein-methionine-S-oxide reductase | 3 | 4 |
| LBP_cg1274 | Alcohol dehydrogenase | 3 | 3 |
| LBP_cg0166 | Alpha-glucosidase | 3 | 3 |
| LBP_cg2729 | Protein of hypothetical function DUF488 | 3 | 3 |
| LBP_cg2270 | Transcription regulator | 3 | 3 |
| LBP_cg2494 | Amino acid transport protein | 3 | 3 |
| LBP_cg1450 | SGNH superfamily hydrolase | 3 | 3 |
| LBP_cg0571 | Diguanylate cyclase/phosphodiesterase domain-containing protein | 3 | 3 |
| LBP_cg0435 | putative septum formation initiator protein | 3 | 3 |
| LBP_cg2775 | Transcription regulator | 3 | 3 |
| LBP_cg1738 | Endopeptidase La (Putative) | 3 | 3 |
| LBP_cg0133 | ABC superfamily ATP binding cassette transporter, ABC protein | 3 | 3 |
| LBP_cg1733 | 30S ribosomal protein S20 | 2 | 103 |
| LBP_cg0393 | DNA-directed RNA polymerase subunit delta | 2 | 16 |
| LBP_cg2779 | hypothetical protein | 2 | 13 |
| LBP_cg0093 | Hydroxyethylthiazole kinase | 2 | 7 |
| LBP_cg2453 | Teichoic acid glycosylation protein GtrA | 2 | 8 |
| LBP_cg1196 | hypothetical protein | 2 | 16 |
| LBP_cg2659 | Zinc-dependent proteinase (Putative) | 2 | 7 |
| LBP_cg2227 | Phosphoribosylformylglycinamidine cyclo-ligase | 2 | 6 |
| LBP_cg2876 | Galactitol PTS, EIIC | 2 | 6 |
| LBP_cg1146 | 50S ribosomal protein L35 | 2 | 12 |
| LBP_cg2337 | hypothetical protein | 2 | 6 |
| LBP_cg0605 | Uracil-DNA glycosylase | 2 | 6 |
| LBP_cg2736 | hypothetical protein | 2 | 8 |
| LBP_cg1123 | hypothetical protein | 2 | 9 |
| LBP_cg1402 | hypothetical protein | 2 | 5 |
| LBP_cg1945 | ATP synthase subunit a | 2 | 7 |
| LBP_cg0108 | Transcription regulator | 2 | 5 |
| LBP_cg0720 | hypothetical protein | 2 | 7 |
| LBP_cg2912 | Sorbitol PTS, EIIA | 2 | 6 |
| LBP_cg1944 | ATP synthase subunit c | 2 | 17 |
| LBP_cg0043 | hypothetical protein | 2 | 4 |
| LBP_cg1682 | hypothetical protein | 2 | 9 |
| LBP_cg1765 | Extracellular protein, gamma-D-glutamate-meso-diaminopimelate muropeptidase (Putative) | 2 | 7 |
| LBP_cg0136 | Butyryl-CoA dehydrogenase | 2 | 4 |
| LBP_cg2309 | Integral membrane protein | 2 | 6 |
| LBP_cg1809 | Transport protein | 2 | 4 |
| LBP_cg0980 | hypothetical protein | 2 | 9 |
| LBP_cg0521 | Oleoyl-acyl-carrier protein | 2 | 5 |
| LBP_cg2691 | Folylpolyglutamate synthase | 2 | 4 |
| LBP_cg2293 | 3-dehydroquinate dehydratase | 2 | 5 |
| LBP_cg2475 | Extracellular protein | 2 | 8 |
| LBP_cg2714 | Cadmium-/zinc-/cobalt-transporting ATPase | 2 | 6 |
| LBP_cg1204 | hypothetical protein | 2 | 4 |
| LBP_cg0068 | Intracellular protease/amidase (Putative) | 2 | 6 |
| LBP_cg1031 | Accessory protein regulator C | 2 | 3 |
| LBP_cg0306 | Transcriptional regulator | 2 | 5 |
| LBP_p3g024 | DNA-damage-inducible protein | 2 | 6 |
| LBP_cg0162 | Sucrose operon repressor | 2 | 6 |
| LBP_p5g015 | hypothetical protein | 2 | 4 |
| LBP_cg1622 | LexA repressor | 2 | 4 |
| LBP_cg0515 | hypothetical protein | 2 | 4 |
| LBP_cg2118 | hypothetical protein | 2 | 6 |
| LBP_cg0237 | putative manganese transport protein mntH | 2 | 5 |
| LBP_cg1723 | MazG nucleotide pyrophosphohydrolase | 2 | 3 |
| LBP_cg2671 | Protein-tyrosine phosphatase | 2 | 3 |
| LBP_cg2072 | Cell surface hydrolase (Putative) | 2 | 3 |
| LBP_cg1896 | Glutamine ABC transporter, permease protein | 2 | 3 |
| LBP_cg2950 | Membrane protein oxaA 1 | 2 | 6 |
| LBP_cg1396 | Integral membrane protein | 2 | 3 |
| LBP_cg0003 | RNA-binding S4 protein | 2 | 3 |
| LBP_cg2590 | Branched-chain amino acid transport protein | 2 | 3 |
| LBP_cg0551 | Sensor protein | 2 | 3 |
| LBP_cg2650 | hypothetical protein | 2 | 4 |
| LBP_cg2783 | hypothetical protein | 2 | 3 |
| LBP_cg0884 | Malolactic regulator | 2 | 4 |
| LBP_cg2665 | Glycerate kinase | 2 | 2 |
| LBP_cg0291 | hypothetical protein | 2 | 3 |
| LBP_cg0780 | hypothetical protein | 2 | 4 |
| LBP_cg0597 | Carboxylesterase | 2 | 4 |
| LBP_cg1718 | UvrABC system protein C | 2 | 3 |
| LBP_cg1877 | hypothetical protein | 2 | 3 |
| LBP_cg0299 | hypothetical protein | 2 | 3 |
| LBP_cg1254 | tRNA (guanine-N(1)-)-methyltransferase | 2 | 5 |
| LBP_cg0659 | Metal uptake regulator | 2 | 4 |
| LBP_cg1834 | Amino acid transport protein | 2 | 4 |
| LBP_cg2392 | GntR family transcriptional regulator | 2 | 3 |
| LBP_cg2626 | Transport protein | 2 | 3 |
| LBP_cg1372 | Integral membrane protein | 2 | 4 |
| LBP_cg2910 | Transcription regulator | 2 | 3 |
| LBP_cg1459 | HAD superfamily hydrolase | 2 | 3 |
| LBP_cg1397 | hypothetical protein | 2 | 6 |
| LBP_cg2935 | DegV family protein | 2 | 2 |
| LBP_cg1753 | hypothetical protein | 2 | 3 |
| LBP_cg2426 | Sugar kinase and transcription regulator | 2 | 2 |
| LBP_cg2686 | Cation efflux protein | 2 | 3 |
| LBP_p3g031 | DNA topoisomerase | 2 | 3 |
| LBP_cg2384 | Hydrolase, HAD superfamily, Cof family | 2 | 3 |
| LBP_cg0625 | hypothetical protein | 2 | 4 |
| LBP_cg2765 | hypothetical protein | 2 | 3 |
| LBP_cg2489 | Aminoacylase | 2 | 2 |
| LBP_cg1990 | Prophage Lp2 protein 40 | 2 | 3 |
| LBP_cg2939 | Signal peptidase I | 2 | 2 |
| LBP_cg0795 | Lysine transport protein | 2 | 3 |
| LBP_cg1680 | Phage anti-repressor protein | 2 | 2 |
| LBP_cg1309 | Integral membrane protein | 2 | 4 |
| LBP_cg0082 | hypothetical protein | 2 | 3 |
| LBP_cg2230 | Phosphoribosylformylglycinamidine synthase 1 | 2 | 2 |
| LBP_cg1534 | Transcription regulator | 2 | 2 |
| LBP_cg0758 | hypothetical protein | 2 | 5 |
| LBP_cg0717 | Acyltransferase | 2 | 3 |
| LBP_cg1833 | putative phosphoesterase | 2 | 2 |
| LBP_cg1826 | Polyribonucleotide nucleotidyltransferase (Putative) | 2 | 2 |
| LBP_cg1137 | Integral membrane protein | 2 | 4 |
| LBP_cg1929 | Amino acid ABC transporter, permease protein | 2 | 2 |
| LBP_cg2902 | Response regulator | 2 | 3 |
| LBP_cg0246 | RpiR family transcriptional regulator | 2 | 3 |
| LBP_cg1492 | Lipoprotein | 2 | 2 |
| LBP_cg1560 | Integral membrane protein | 2 | 3 |
| LBP_cg0983 | Purine/pyrimidine phosphoribosyltransferase (Putative) | 2 | 3 |
| LBP_cg1409 | hypothetical protein | 2 | 4 |
| LBP_cg1703 | Transcription regulator of fructose operon | 2 | 3 |
| LBP_cg2216 | Xanthine / uracil transport protein | 2 | 2 |
| LBP_cg1407 | APC family amino acid-polyamine-organocation transporter | 2 | 2 |
| LBP_cg1267 | Tryptophan synthase subunit beta | 2 | 2 |
| LBP_cg2147 | Transcription regulator | 2 | 3 |
| LBP_cg0165 | Na(+)/H(+) antiporter | 2 | 3 |
| LBP_cg2291 | cell surface protein precursor | 2 | 2 |
| LBP_cg2458 | hypothetical protein | 2 | 2 |
| LBP_cg0463 | Homoserine kinase | 2 | 2 |
| LBP_cg0588 | RNA polymerase factor sigma-54 | 2 | 3 |
| LBP_cg0964 | Oligopeptide ABC superfamily ATP binding cassette transporter, permease protein | 2 | 2 |
| LBP_cg2239 | Carbonate dehydratase | 2 | 3 |
| LBP_cg2849 | Sugar phosphate isomerase | 2 | 2 |
| LBP_cg2697 | ABC superfamily ATP binding cassette transporter, binding protein | 2 | 2 |
| LBP_cg0915 | P-ATPase superfamily cation transporter | 2 | 2 |
| LBP_cg1964 | ABC transporter, ATP-binding and permease protein | 2 | 2 |
| LBP_cg2423 | Amidohydrolase 2 | 2 | 4 |
| LBP_cg1418 | Glutathione reductase | 2 | 2 |
| LBP_cg0904 | hypothetical protein | 2 | 4 |
| LBP_cg2528 | NmrA family protein | 2 | 2 |
| LBP_cg0033 | hypothetical protein | 2 | 2 |
| LBP_cg1219 | Geranyltranstransferase | 2 | 3 |
| LBP_cg0985 | hypothetical protein | 2 | 3 |
| LBP_cg1221 | Arginine repressor | 2 | 3 |
| LBP_cg0762 | Flavoprotein | 2 | 3 |
| LBP_cg1033 | Extracellular protein, membrane-anchored (Putative) | 2 | 3 |
| LBP_cg1664 | hypothetical protein | 2 | 3 |
| LBP_cg2619 | Integral membrane protein (Putative) | 2 | 3 |
| LBP_cg2679 | Permease | 2 | 3 |
| LBP_cg2790 | hypothetical protein | 2 | 3 |
| LBP_cg0250 | LytR family transcriptional regulator | 2 | 2 |
| LBP_cg0564 | diguanylate cyclase | 2 | 4 |
| LBP_cg2805 | Cell surface protein | 2 | 3 |
| LBP_cg0935 | Glycerol uptake facilitator protein | 2 | 3 |
| LBP_cg2777 | Transcription regulator | 2 | 2 |
| LBP_cg2677 | putative potassium transport system protein kup 2 | 2 | 2 |
| LBP_p1g002 | phosphohydrolase | 2 | 2 |
| LBP_cg0032 | hypothetical protein | 2 | 2 |
| LBP_cg2331 | Methylated-DNA-(Protein)-cysteine S-methyltransferase | 2 | 4 |
| LBP_cg2520 | HAD superfamily hydrolase | 2 | 2 |
| LBP_cg0121 | hypothetical protein | 2 | 2 |
| LBP_cg0471 | Mannose PTS, EIIB | 2 | 2 |
| LBP_cg0414 | PemK family growth inhibitor | 2 | 3 |
| LBP_cg1968 | Abi family protein | 2 | 2 |
| LBP_cg0917 | Cell surface hydrolase (Putative) | 2 | 2 |
| LBP_cg1636 | Prophage Lp1 protein 58, lysin | 2 | 2 |
| LBP_cg2625 | Integral membrane protein | 2 | 2 |
| LBP_cg0216 | hypothetical protein | 2 | 2 |
| LBP_cg1417 | Transcription regulator | 2 | 2 |
| LBP_cg2315 | Na(+)/H(+) antiporter | 2 | 2 |
| LBP_p2g010 | GPH family glycoside-pentoside-hexuronide:cation symporter | 2 | 2 |
| LBP_cg2433 | Haloacid dehalogenase-like family hydrolase | 2 | 2 |
| LBP_cg1566 | HAD superfamily hydrolase | 2 | 2 |
| LBP_cg1259 | putative sulfate-transporting ATPase | 2 | 2 |
| LBP_cg2131 | Nitroreductase | 2 | 3 |
| LBP_cg2321 | 2-haloacid dehalogenase (Putative) | 2 | 2 |
| LBP_cg1452 | Dihydrofolate reductase | 2 | 3 |
| LBP_cg2212 | Dihydroorotase | 2 | 2 |
| LBP_cg1779 | hypothetical protein | 2 | 3 |
| LBP_cg2685 | Transcription regulator | 2 | 2 |
| LBP_cg1959 | putative beta-lactamase | 2 | 2 |
| LBP_cg1009 | hypothetical protein | 2 | 2 |
| LBP_cg1191 | Integral membrane protein | 2 | 2 |
| LBP_cg0896 | hypothetical protein | 2 | 3 |
| LBP_cg1395 | Acetyltransferase | 2 | 2 |
| LBP_cg0095 | Thiamine-phosphate pyrophosphorylase | 2 | 2 |
| LBP_cg1567 | D-tyrosyl-tRNA(Tyr) deacylase | 2 | 2 |
| LBP_cg1953 | N5-glutamine S-adenosyl-L-methionine-dependent methyltransferase | 2 | 2 |
| LBP_cg1381 | Carbamoyl phosphate synthase small subunit | 2 | 3 |
| LBP_cg2460 | ArsR family transcriptional regulator | 2 | 2 |
| LBP_cg0234 | hypothetical protein | 2 | 2 |
| LBP_cg0920 | hypothetical protein | 2 | 2 |
| LBP_p3g018 | NCS2 family nucleobase:cation symporter-2 | 2 | 2 |
| LBP_cg0107 | Oxidoreductase (Putative) | 2 | 2 |
| LBP_cg1741 | hypothetical protein | 2 | 3 |
| LBP_cg0569 | hypothetical protein | 2 | 2 |
| LBP_cg0315 | Phospholipid-binding protein | 2 | 3 |
| LBP_cg2546 | Pyrophosphatase (Putative) | 2 | 2 |
| LBP_cg2636 | hypothetical protein | 2 | 2 |
| LBP_cg1808 | 30S ribosomal protein S14 | 2 | 2 |
| LBP_cg2069 | MutT/nudix family hydrolase | 2 | 2 |
| LBP_cg0495 | 50S ribosomal protein L33 | 2 | 2 |
| LBP_cg0076 | hypothetical protein | 2 | 2 |
| LBP_cg0468 | hypothetical protein | 2 | 2 |
| LBP_cg0149 | Maltose/maltodextrin ABC transporter, permease protein | 2 | 2 |
| LBP_cg2610 | hypothetical protein | 2 | 2 |
| LBP_cg2868 | Transketolase | 2 | 2 |
| LBP_cg0223 | hypothetical protein | 2 | 3 |
| LBP_cg0316 | hypothetical protein | 2 | 2 |
| LBP_cg0444 | POT family proton (H+)-dependent oligopeptide transporter | 2 | 2 |
| LBP_cg1721 | hypothetical protein | 2 | 2 |
| LBP_cg0070 | Transcription regulator | 2 | 2 |
| LBP_cg0194 | ABC transporter, ATP-binding protein | 2 | 2 |
| LBP_cg1674 | Hel protein | 2 | 2 |
| LBP_cg2699 | hypothetical protein | 2 | 2 |
| LBP_cg2716 | hypothetical protein | 2 | 2 |
| LBP_cg0347 | Cellobiose PTS, EIIC | 2 | 2 |
| LBP_cg0603 | hypothetical protein | 2 | 2 |
| LBP_cg2052 | ABC superfamily ATP binding cassette transporter, ATP-binding and permease protein | 2 | 3 |
| LBP_p3g025 | hypothetical protein | 2 | 2 |
| LBP_cg2811 | hypothetical protein | 2 | 2 |
| LBP_cg2555 | Cellobiose PTS, EIIC | 2 | 2 |
| LBP_cg1623 | hypothetical protein | 2 | 2 |
| LBP_cg1663 | hypothetical protein | 2 | 2 |
| LBP_cg2769 | Extracellular protein | 2 | 2 |
