# Supplementary material for: Adaptation of Lactobacillus plantarum to Ampicillin Involves Mechanisms That Maintain Protein Homeostasis
Source: mSystems. 2020 Jan 28;5(1):e00853-19. doi: 10.1128/mSystems.00853-19 (PMC6989132; doi:10.1128/mSystems.00853-19)
Supplement: TABLE S3 [file mSystems.00853-19-st003.docx]

**Table S3.** Up-regulated proteins of *L. plantarum* 400g relative to *L. plantarum* P-8 grown in the presence of the ampicillin

| **Accession** | **COG category** | **Description** | **Protein ID** | **MW [kDa]** | **calc. pI** | **Fold change** | **T test p value** |
| --- | --- | --- | --- | --- | --- | --- | --- |
| LBP_p3g026 | - | hypothetical protein | AGL65764.2 | 11.1 | 9.73 | 3.01 | 2.15E-04 |
| LBP_cg0885 | COG0281 [C] | Malate dehydrogenase | AGL63631.2 | 59.5 | 4.89 | 2.83 | 2.35E-05 |
| LBP_cg1738 | COG3480 [T] | Endopeptidase La (Putative) | AGL64484.2 | 38.5 | 10.05 | 2.61 | 7.94E-04 |
| LBP_cg1450 | COG2755 [E] | SGNH superfamily hydrolase | AGL64196.2 | 35.8 | 9.86 | 2.24 | 3.85E-05 |
| LBP_cg0780 | - | hypothetical protein | AGL63526.2 | 26.8 | 10.48 | 2.01 | 5.81E-04 |
| LBP_cg2150 | COG1073 [R] | Cell surface hydrolase, membrane-bound (Putative) | AGL64896.2 | 34.2 | 9.98 | 1.70 | 2.81E-03 |
| LBP_cg1116 | COG0760 [O] | Foldase protein prsA 1 | AGL63862.2 | 32.6 | 9.77 | 1.68 | 3.54E-04 |
| LBP_cg0504 | - | Prophage Lp1 protein 66, lipoprotein | AGL63250.2 | 23.2 | 10.68 | 1.60 | 9.99E-03 |
| LBP_cg1330 | - | hypothetical protein | AGL64076.2 | 30.3 | 5.60 | 1.60 | 1.37E-02 |
| LBP_cg1866 | COG4472 [S] | hypothetical protein | AGL64612.2 | 10.3 | 5.06 | 1.56 | 1.69E-03 |
| LBP_cg1564 | COG0860 [M] | N-acetylmuramoyl-L-alanine amidase | AGL64310.2 | 31.0 | 9.66 | 1.56 | 1.01E-04 |
| LBP_cg0964 | COG0601 [EP] | Oligopeptide ABC superfamily ATP binding cassette transporter, permease protein | AGL63710.2 | 33.7 | 9.11 | 1.53 | 2.45E-02 |
| LBP_cg0965 | COG1173 [EP] | Oligopeptide ABC transporter, permease protein | AGL63711.2 | 37.6 | 9.31 | 1.50 | 3.22E-04 |
| LBP_cg1294 | COG1607 [I] | Acyl-CoA thioester hydrolase (Putative) | AGL64040.2 | 18.6 | 6.28 | 1.50 | 1.38E-03 |
| LBP_cg2626 | COG0390 [R] | Transport protein | AGL65372.2 | 27.1 | 9.95 | 1.49 | 1.57E-03 |
| LBP_cg2613 | COG0834 [ET] | Amino acid ABC superfamily ATP binding cassette transporter, binding protein | AGL65359.2 | 29.0 | 9.96 | 1.49 | 4.43E-04 |
| LBP_cg1703 | COG1349 [KG] | Transcription regulator of fructose operon | AGL64449.2 | 27.7 | 5.81 | 1.48 | 1.76E-04 |
| LBP_cg2868 | COG0021 [G] | Transketolase | AGL65614.2 | 71.9 | 6.06 | 1.46 | 3.24E-03 |
| LBP_cg0397 | COG1760 [E] | L-serine dehydratase, beta subunit | AGL63143.2 | 23.8 | 5.95 | 1.46 | 4.24E-04 |
| LBP_cg1218 | COG1722 [L] | Exodeoxyribonuclease 7 small subunit | AGL63964.2 | 8.4 | 4.01 | 1.43 | 9.74E-03 |
| LBP_cg2450 | - | hypothetical protein | AGL65196.2 | 8.2 | 4.56 | 1.43 | 7.30E-03 |
| LBP_cg1196 | - | hypothetical protein | AGL63942.2 | 6.4 | 10.11 | 1.41 | 7.81E-04 |
| LBP_cg0569 | - | hypothetical protein | AGL63315.2 | 17.3 | 9.70 | 1.40 | 1.08E-03 |
| LBP_cg1441 | COG0620 [E] | putative 5-methyltetrahydropteroyltriglutamate--homocysteine S-methyltransferase | AGL64187.2 | 43.2 | 5.33 | 1.39 | 1.37E-03 |
| LBP_cg1931 | COG0509 [E] | Glycine cleavage system, H protein | AGL64677.2 | 10.5 | 4.41 | 1.39 | 1.22E-02 |
| LBP_cg1567 | COG1490 [J] | D-tyrosyl-tRNA(Tyr) deacylase | AGL64313.2 | 16.0 | 5.21 | 1.39 | 1.27E-03 |
| LBP_cg0217 | COG0431 [R] | putative NAD(P)H dehydrogenase (Quinone) | AGL62963.2 | 24.6 | 6.70 | 1.39 | 7.10E-04 |
| LBP_cg2627 | COG4619 [R] | ABC superfamily ATP binding cassette transporter, ABC protein | AGL65373.2 | 24.0 | 6.33 | 1.38 | 4.12E-04 |
| LBP_cg1808 | COG0199 [J] | 30S ribosomal protein S14 | AGL64554.2 | 10.1 | 11.43 | 1.38 | 8.45E-03 |
| LBP_p3g011 | - | Peptidoglycan-binding protein | AGL65749.2 | 31.3 | 9.50 | 1.38 | 1.42E-03 |
| LBP_cg1786 | COG1799 [S] | Cell division protein sepF | AGL64532.2 | 15.3 | 4.54 | 1.38 | 7.07E-05 |
| LBP_cg1232 | COG0223 [J] | Methionyl-tRNA formyltransferase | AGL63978.2 | 34.4 | 7.06 | 1.37 | 1.01E-04 |
| LBP_cg0590 | COG0057 [G] | Glyceraldehyde 3-phosphate dehydrogenase | AGL63336.2 | 36.4 | 5.54 | 1.36 | 8.64E-03 |
| LBP_cg0398 | COG1760 [E] | L-serine dehydratase, alpha subunit | AGL63144.2 | 30.7 | 5.68 | 1.36 | 1.74E-03 |
| LBP_cg0208 | COG4668 [G] | Mannitol PTS, EIIA | AGL62954.2 | 16.1 | 4.41 | 1.36 | 3.12E-03 |
| LBP_cg0009 | COG0629 [L] | Single-stranded DNA-binding protein | AGL62755.2 | 20.9 | 5.12 | 1.36 | 4.73E-04 |
| LBP_cg2288 | COG0716 [C] | Flavodoxin | AGL65034.2 | 16.0 | 4.13 | 1.36 | 3.68E-02 |
| LBP_cg0753 | COG0017 [J] | Asparaginyl-tRNA synthetase | AGL63499.2 | 49.0 | 5.24 | 1.36 | 3.02E-04 |
| LBP_cg0491 | COG1939 [S] | Ribonuclease III | AGL63237.2 | 15.3 | 7.64 | 1.36 | 1.50E-03 |
| LBP_cg1928 | COG1464 [P] | ABC superfamily ATP binding cassette transporter, binding protein | AGL64674.2 | 29.9 | 9.85 | 1.35 | 1.14E-05 |
| LBP_cg0395 | COG0172 [J] | Seryl-tRNA synthetase 1 | AGL63141.2 | 47.8 | 5.64 | 1.35 | 6.88E-04 |
| LBP_cg1290 | COG0623 [I] | Enoyl-(Acyl carrier protein) reductase | AGL64036.2 | 26.9 | 7.44 | 1.34 | 3.09E-04 |
| LBP_cg1341 | COG0017 [J] | Asparaginyl-tRNA synthetase 2 | AGL64087.2 | 49.8 | 5.11 | 1.34 | 2.43E-04 |
| LBP_cg1611 | COG0233 [J] | Ribosome-recycling factor | AGL64357.2 | 20.6 | 6.11 | 1.34 | 1.55E-02 |
| LBP_cg0357 | COG0846 [K] | NAD-dependent deacetylase (Regulatory protein SIR2 family protein) | AGL63103.2 | 26.5 | 5.80 | 1.34 | 7.32E-04 |
| LBP_p6g001 | COG3409 [M] | YkuG protein | AGL65828.2 | 83.1 | 5.55 | 1.33 | 1.50E-03 |
| LBP_cg1666 | COG0270 [L] | Modification methylase Rho11sI family protein | AGL64412.2 | 37.6 | 8.07 | 1.32 | 4.15E-02 |
| LBP_cg1038 | - | hypothetical protein | AGL63784.2 | 10.6 | 4.81 | 1.32 | 4.66E-03 |
| LBP_cg1862 | COG1193 [L] | MutS2 protein | AGL64608.2 | 87.2 | 6.58 | 1.32 | 8.74E-04 |
| LBP_cg2781 | COG0791 [M] | Extracellular protein, gamma-D-glutamate-meso-diaminopimelate muropeptidase (Putative) | AGL65527.2 | 34.7 | 9.39 | 1.31 | 1.52E-02 |
| LBP_cg2828 | COG1087 [M] | UDP-glucose 4-epimerase | AGL65574.2 | 36.4 | 5.53 | 1.31 | 5.17E-03 |
| LBP_cg1851 | - | hypothetical protein | AGL64597.2 | 18.0 | 4.48 | 1.31 | 1.15E-03 |
| LBP_cg2919 | COG1869 [G] | D-ribose pyranase | AJF17191.1 | 14.6 | 5.97 | 1.31 | 1.11E-03 |
| LBP_cg2334 | COG1668 [CP] | ABC transporter, permease protein (Putative) | AGL65080.2 | 44.9 | 9.99 | 1.31 | 4.09E-04 |
| LBP_cg1704 | COG1105 [G] | 1-phosphofructokinase | AGL64450.2 | 32.4 | 5.15 | 1.30 | 5.46E-04 |
| LBP_cg2768 | COG4086 [S] | Extracellular protein | AGL65514.2 | 34.2 | 9.51 | 1.30 | 2.50E-05 |
| LBP_cg0797 | COG1876 [M] | Serine-type D-Ala-D-Ala carboxypeptidase | AGL63543.2 | 26.9 | 9.61 | 1.30 | 4.84E-04 |
| LBP_cg2593 | COG1686 [M] | Serine family D-Ala-D-Ala carboxypeptidase | AGL65339.2 | 47.0 | 9.91 | 1.30 | 3.54E-03 |
| LBP_cg0912 | COG1597 [IR] | Diacylglycerol kinase | AGL63658.2 | 37.0 | 5.49 | 1.30 | 3.39E-04 |
| LBP_cg0967 | COG4608 [E] | ABC superfamily ATP binding cassette transporter, ABC protein | AGL63713.2 | 36.6 | 6.02 | 1.30 | 2.63E-04 |
| LBP_cg1396 | COG5523 [S] | Integral membrane protein | AGL64142.2 | 27.7 | 9.61 | 1.30 | 1.61E-03 |
| LBP_cg2268 | COG1827 [R] | Transcriptional regulator | AGL65014.2 | 18.7 | 7.20 | 1.29 | 3.81E-03 |
| LBP_cg2620 | - | hypothetical protein | AGL65366.2 | 33.4 | 8.40 | 1.29 | 2.57E-03 |
| LBP_cg0358 | COG1801 [S] | hypothetical protein | AGL63104.2 | 31.8 | 5.97 | 1.29 | 5.60E-03 |
| LBP_cg1793 | COG0768 [M] | Penicillin binding protein 2B | AGL64539.2 | 77.2 | 9.85 | 1.29 | 2.35E-03 |
| LBP_cg1697 | - | hypothetical protein | AGL64443.2 | 12.7 | 10.08 | 1.28 | 2.06E-04 |
| LBP_cg2597 | COG0760 [O] | Peptidylprolyl isomerase | AGL65343.2 | 34.3 | 9.98 | 1.28 | 4.07E-02 |
| LBP_cg1584 | COG1680 [V] | Serine-type D-Ala-D-Ala carboxypeptidase | AGL64330.2 | 43.3 | 9.80 | 1.28 | 8.51E-03 |
| LBP_cg0396 | COG0814 [E] | Serine transporter | AGL63142.2 | 46.9 | 9.39 | 1.28 | 4.22E-03 |
| LBP_cg0209 | COG0246 [G] | Mannitol-1-phosphate 5-dehydrogenase | AGL62955.2 | 43.2 | 5.34 | 1.28 | 5.86E-03 |
| LBP_cg0386 | - | Lipoprotein | AGL63132.2 | 34.3 | 6.00 | 1.28 | 4.22E-02 |
| LBP_cg0519 | COG4467 [S] | Initiation-control protein yabA | AGL63265.2 | 13.6 | 5.00 | 1.28 | 3.18E-03 |
| LBP_cg1354 | COG3599 [D] | Cell cycle protein gpsB | AGL64100.2 | 12.9 | 6.57 | 1.28 | 4.58E-03 |
| LBP_cg0587 | COG0740 [OU] | ATP-dependent Clp protease proteolytic subunit | AGL63333.2 | 21.5 | 5.02 | 1.28 | 9.76E-03 |
| LBP_cg1663 | - | hypothetical protein | AGL64409.2 | 15.2 | 4.51 | 1.27 | 2.24E-02 |
| LBP_cg0688 | COG0406 [G] | Phosphoglycerate mutase | AGL63434.2 | 25.8 | 6.16 | 1.27 | 3.12E-02 |
| LBP_cg1397 | - | hypothetical protein | AGL64143.2 | 25.3 | 9.72 | 1.27 | 2.98E-04 |
| LBP_cg2809 | COG1309 [K] | Transcription regulator | AGL65555.2 | 22.1 | 6.38 | 1.27 | 1.90E-03 |
| LBP_cg2248 | COG0589 [T] | Universal stress protein UspA | AGL64994.2 | 17.1 | 4.94 | 1.27 | 5.58E-04 |
| LBP_cg0788 | COG1316 [K] | Transcription regulator | AGL63534.2 | 37.7 | 9.89 | 1.26 | 1.89E-03 |
| LBP_cg0671 | COG0765 [E] | Glutamine ABC transporter, permease protein | AGL63417.2 | 23.7 | 9.09 | 1.26 | 1.21E-02 |
| LBP_cg2779 | - | hypothetical protein | AGL65525.2 | 7.4 | 6.28 | 1.26 | 3.68E-02 |
| LBP_cg1077 | COG1438 [K] | Arginine regulator | AGL63823.2 | 17.1 | 5.06 | 1.26 | 6.89E-03 |
| LBP_cg2628 | COG2265 [J] | putative RNA methyltransferase | AGL65374.2 | 53.7 | 7.01 | 1.26 | 1.49E-04 |
| LBP_cg2939 | COG0681 [U] | Signal peptidase I | AJF17211.1 | 22.4 | 9.89 | 1.26 | 1.88E-03 |
| LBP_p5g002 | - | putative cell surface protein | AGL65811.2 | 67.0 | 9.85 | 1.25 | 4.20E-02 |
| LBP_cg2590 | COG1296 [E] | Branched-chain amino acid transport protein | AGL65336.2 | 28.2 | 8.87 | 1.25 | 2.98E-03 |
| LBP_cg1425 | COG0225 [O] | Protein-methionine-S-oxide reductase | AGL64171.2 | 19.2 | 5.20 | 1.25 | 4.29E-02 |
| LBP_cg1052 | - | Arylsulfate sulfotransferase | AGL63798.2 | 64.6 | 10.02 | 1.25 | 1.09E-03 |
| LBP_cg2289 | - | Cell surface protein | AGL65035.2 | 113.1 | 4.48 | 1.25 | 1.37E-02 |
| LBP_cg2160 | COG0526 [OC] | Thioredoxin H-type | AGL64906.2 | 12.3 | 4.70 | 1.24 | 9.30E-03 |
| LBP_cg2610 | COG4814 [R] | hypothetical protein | AGL65356.2 | 30.8 | 11.12 | 1.24 | 9.02E-05 |
| LBP_cg2625 | COG1434 [S] | Integral membrane protein | AGL65371.2 | 41.5 | 9.85 | 1.24 | 3.42E-02 |
| LBP_cg2118 | - | hypothetical protein | AGL64864.2 | 8.1 | 6.10 | 1.24 | 8.30E-03 |
| LBP_cg0319 | COG0511 [I] | Acetyl-CoA carboxylase, biotin carboxyl carrier protein | AGL63065.2 | 20.5 | 4.58 | 1.24 | 1.56E-02 |
| LBP_cg1920 | COG0589 [T] | Universal stress protein UspA | AGL64666.2 | 17.5 | 6.33 | 1.24 | 1.35E-03 |
| LBP_cg1589 | COG3527 [Q] | Alpha-acetolactate decarboxylase | AGL64335.2 | 25.9 | 5.27 | 1.24 | 3.55E-02 |
| LBP_cg0520 | COG0313 [R] | Methyltransferase (Putative) | AGL63266.2 | 32.9 | 6.24 | 1.24 | 5.53E-03 |
| LBP_cg2648 | COG0735 [P] | Ferric uptake regulator | AGL65394.2 | 18.1 | 6.43 | 1.24 | 1.07E-03 |
| LBP_cg1476 | COG0469 [G] | Pyruvate kinase | AGL64222.2 | 62.8 | 5.14 | 1.23 | 3.83E-04 |
| LBP_cg0250 | COG1316 [K] | LytR family transcriptional regulator | AGL62996.2 | 37.6 | 9.76 | 1.23 | 4.92E-03 |
| LBP_cg1464 | COG0283 [F] | Cytidylate kinase | AGL64210.2 | 24.6 | 8.27 | 1.23 | 2.20E-04 |
| LBP_cg1046 | COG0620 [E] | 5-methyltetrahydropteroyltriglutamate--homocysteine methyltransferase | AGL63792.2 | 86.4 | 6.11 | 1.23 | 3.77E-04 |
| LBP_cg2629 | - | hypothetical protein | AGL65375.2 | 16.8 | 6.54 | 1.23 | 3.37E-04 |
| LBP_cg1858 | COG0127 [F] | Nucleoside-triphosphatase | AGL64604.2 | 21.8 | 6.96 | 1.23 | 1.12E-03 |
| LBP_cg0614 | - | Transcription regulator | AGL63360.2 | 19.8 | 8.32 | 1.23 | 1.20E-02 |
| LBP_cg0585 | COG4166 [E] | Lipoprotein, peptide binding protein OppA-like protein | AGL63331.2 | 61.1 | 9.67 | 1.23 | 3.00E-02 |
| LBP_cg1852 | COG4768 [R] | Methyl-accepting chemotaxis family protein | AGL64598.2 | 15.0 | 8.69 | 1.23 | 4.50E-04 |
| LBP_cg0613 | COG0025 [P] | Na(+)/H(+) antiporter (Putative) | AGL63359.2 | 78.2 | 6.68 | 1.23 | 4.62E-03 |
| LBP_cg1165 | COG3212 [S] | Lipoprotein | AGL63911.2 | 21.0 | 5.59 | 1.23 | 6.34E-03 |
| LBP_cg1695 | COG0503 [F] | Adenine phosphoribosyltransferase | AGL64441.2 | 18.9 | 5.25 | 1.23 | 1.00E-03 |
| LBP_cg1856 | COG0517 [R] | Cystathionine beta-synthase (CBS) domain protein | AGL64602.2 | 18.7 | 5.01 | 1.22 | 7.50E-03 |
| LBP_cg0492 | COG0566 [J] | tRNA/rRNA methyltransferase | AGL63238.2 | 27.5 | 8.75 | 1.22 | 4.40E-04 |
| LBP_cg0966 | COG0444 [EP] | Oligopeptide ABC transporter, ATP-binding protein | AGL63712.2 | 39.7 | 5.87 | 1.22 | 1.61E-02 |
| LBP_cg0928 | - | hypothetical protein | AGL63674.2 | 16.2 | 4.18 | 1.22 | 2.23E-03 |
| LBP_cg0477 | COG1316 [K] | Transcription regulator | AGL63223.2 | 43.6 | 9.79 | 1.22 | 2.93E-03 |
| LBP_cg1447 | COG0793 [M] | S41 family carboxy-terminal processing peptidase | AGL64193.2 | 53.2 | 9.82 | 1.22 | 1.76E-03 |
| LBP_cg1961 | COG0115 [EH] | Branched-chain-amino-acid aminotransferase | AGL64707.2 | 37.9 | 5.82 | 1.22 | 1.66E-03 |
| LBP_cg0206 | COG2213 [G] | Protein-N(Pi)-phosphohistidine--sugar phosphotransferase | AGL62952.2 | 63.8 | 8.65 | 1.22 | 9.33E-03 |
| LBP_cg1318 | COG1942 [R] | putative tautomerase | AGL64064.2 | 7.0 | 5.73 | 1.22 | 9.83E-03 |
| LBP_cg2833 | COG2190 [G] | Sugar transport protein | AGL65579.2 | 71.0 | 5.67 | 1.22 | 2.15E-03 |
| LBP_cg2615 | COG1126 [E] | ABC superfamily ATP binding cassette transporter, ABC protein | AGL65361.2 | 28.1 | 5.86 | 1.22 | 1.01E-03 |
| LBP_cg1237 | COG0036 [G] | Ribulose-phosphate 3-epimerase | AGL63983.2 | 23.3 | 4.91 | 1.22 | 9.86E-03 |
| LBP_cg1682 | - | hypothetical protein | AGL64428.2 | 4.6 | 5.91 | 1.22 | 3.21E-02 |
| LBP_cg2202 | COG0702 [MG] | hypothetical protein | AGL64948.2 | 22.9 | 5.63 | 1.21 | 2.60E-03 |
| LBP_p3g034 | - | hypothetical protein | AGL65772.2 | 16.9 | 9.69 | 1.21 | 3.14E-02 |
| LBP_cg2677 | COG3158 [P] | putative potassium transport system protein kup 2 | AGL65423.2 | 75.6 | 9.61 | 1.21 | 7.99E-03 |
| LBP_cg1598 | COG0858 [J] | Ribosome-binding factor A | AGL64344.2 | 13.1 | 7.25 | 1.21 | 4.60E-04 |
| LBP_cg0434 | COG1188 [J] | S4 RNA-binding domain protein | AGL63180.2 | 10.9 | 9.41 | 1.21 | 4.56E-03 |
| LBP_cg2330 | - | Extracellular protein | AGL65076.2 | 33.2 | 9.55 | 1.21 | 1.61E-02 |
| LBP_cg1677 | - | hypothetical protein | AGL64423.2 | 9.0 | 4.88 | 1.20 | 3.81E-02 |
