# Supplementary material for: Adaptation of Lactobacillus plantarum to Ampicillin Involves Mechanisms That Maintain Protein Homeostasis
Source: mSystems. 2020 Jan 28;5(1):e00853-19. doi: 10.1128/mSystems.00853-19 (PMC6989132; doi:10.1128/mSystems.00853-19)
Supplement: TABLE S4 [file mSystems.00853-19-st004.docx]

**Table S4**. Down-regulated proteins of *L. plantarum* 400g relative to *L. plantarum* P-8 grown in the presence of the ampicillin

| **Accession** | **COG category** | **Description** | **Protein ID** | **MW [kDa]** | **calc. pI** | **Fold change** | **T test p value** |
| --- | --- | --- | --- | --- | --- | --- | --- |
| LBP_cg1170 | COG0642 [T] | Sensor protein | AGL63916.2 | 62.0 | 6.79 | 0.83 | 7.39E-03 |
| LBP_cg2270 | COG1309 [K] | Transcription regulator | AGL65016.2 | 25.3 | 6.23 | 0.83 | 2.51E-03 |
| LBP_p2g040 | COG1192 [D] | ATPase involved in chromosome partitioning | AGL65736.2 | 30.0 | 6.09 | 0.83 | 9.37E-04 |
| LBP_cg1618 | COG4123 [R] | Methyltransferase (Putative) | AGL64364.2 | 28.3 | 7.24 | 0.83 | 3.01E-04 |
| LBP_cg0985 | - | hypothetical protein | AGL63731.2 | 19.7 | 9.10 | 0.83 | 8.74E-03 |
| LBP_cg1451 | COG1307 [S] | DegV family protein | AGL64197.2 | 30.1 | 6.09 | 0.83 | 4.45E-05 |
| LBP_cg1238 | COG1564 [H] | hypothetical protein | AGL63984.2 | 24.1 | 5.03 | 0.83 | 1.23E-03 |
| LBP_cg1897 | COG2894 [D] | Septum site-determining protein MinD | AGL64643.2 | 29.1 | 5.21 | 0.83 | 4.94E-04 |
| LBP_cg0515 | - | hypothetical protein | AGL63261.2 | 9.8 | 9.79 | 0.82 | 4.79E-03 |
| LBP_cg1423 | - | hypothetical protein | AGL64169.2 | 32.9 | 8.48 | 0.82 | 3.49E-03 |
| LBP_cg1956 | COG0769 [M] | UDP-N-acetylmuramyl tripeptide synthase (Putative) | AGL64702.2 | 49.7 | 7.84 | 0.82 | 9.62E-03 |
| LBP_cg2204 | COG1074 [L] | ATP-dependent nuclease, subunit A | AGL64950.2 | 140.9 | 5.29 | 0.82 | 4.15E-05 |
| LBP_cg0353 | COG0042 [J] | tRNA-dihydrouridine synthase | AGL63099.2 | 39.5 | 7.17 | 0.82 | 3.43E-03 |
| LBP_cg1913 | COG4493 [S] | hypothetical protein | AGL64659.2 | 23.5 | 8.13 | 0.82 | 2.28E-02 |
| LBP_cg2742 | COG3049 [M] | Bile salt hydrolase | AGL65488.2 | 36.1 | 5.44 | 0.82 | 2.04E-04 |
| LBP_cg1877 | - | hypothetical protein | AGL64623.2 | 19.4 | 4.98 | 0.82 | 1.96E-03 |
| LBP_cg2131 | COG0778 [C] | Nitroreductase | AGL64877.2 | 20.0 | 5.81 | 0.82 | 2.32E-03 |
| LBP_cg0054 | COG0637 [R] | Beta-phosphoglucomutase | AGL62800.2 | 26.2 | 4.82 | 0.81 | 4.81E-03 |
| LBP_cg0604 | COG0561 [R] | HAD superfamily hydrolase | AGL63350.2 | 31.3 | 5.11 | 0.81 | 7.28E-04 |
| LBP_cg2836 | COG0667 [C] | Aldo/keto reductase family protein | AGL65582.2 | 37.9 | 6.81 | 0.81 | 4.37E-02 |
| LBP_cg0552 | COG0226 [P] | Phosphate ABC superfamily ATP binding cassette transporter, substrate binding protein | AGL63298.2 | 31.4 | 9.61 | 0.81 | 2.58E-02 |
| LBP_cg0624 | COG2017 [G] | Aldose 1-epimerase | AGL63370.2 | 38.0 | 6.77 | 0.81 | 1.35E-03 |
| LBP_cg2533 | COG4221 [R] | Short-chain dehydrogenase/oxidoreductase | AGL65279.2 | 26.3 | 6.29 | 0.81 | 2.16E-02 |
| LBP_cg1927 | COG0039 [C] | L-2-hydroxyisocaproate dehydrogenase | AGL64673.2 | 32.5 | 5.35 | 0.81 | 1.65E-02 |
| LBP_cg0399 | COG2964 [S] | hypothetical protein | AGL63145.2 | 27.1 | 5.99 | 0.81 | 7.39E-03 |
| LBP_cg2702 | COG1680 [V] | putative beta-lactamase | AGL65448.2 | 37.3 | 5.78 | 0.81 | 4.79E-07 |
| LBP_cg0851 | COG1307 [S] | putative spore protein YitS | AGL63597.2 | 32.4 | 6.23 | 0.81 | 2.58E-03 |
| LBP_cg2438 | COG2984 [R] | ATP-binding cassette transporter | AGL65184.2 | 33.9 | 9.79 | 0.81 | 2.24E-03 |
| LBP_cg0550 | COG0745 [TK] | Response regulator | AGL63296.2 | 27.2 | 5.97 | 0.81 | 2.80E-03 |
| LBP_cg1834 | COG1113 [E] | Amino acid transport protein | AGL64580.2 | 50.7 | 9.42 | 0.81 | 4.95E-02 |
| LBP_cg1783 | COG3599 [D] | Cell division initiation protein DivIVA | AGL64529.2 | 26.2 | 4.65 | 0.81 | 9.49E-04 |
| LBP_cg0617 | COG4856 [S] | YbbR like protein | AGL63363.2 | 34.6 | 9.52 | 0.81 | 2.93E-05 |
| LBP_cg1883 | COG0249 [L] | DNA mismatch repair protein mutS | AGL64629.2 | 100.0 | 5.50 | 0.81 | 5.89E-05 |
| LBP_cg1172 | COG0419 [L] | Exonuclease SbcC | AGL63918.2 | 118.0 | 5.71 | 0.80 | 4.77E-05 |
| LBP_cg2273 | COG3048 [E] | putative D-serine dehydratase | AGL65019.2 | 47.6 | 5.82 | 0.80 | 1.80E-02 |
| LBP_cg1524 | COG0583 [K] | Transcription regulator | AGL64270.2 | 32.9 | 6.90 | 0.80 | 3.91E-04 |
| LBP_cg2254 | - | NUDIX family hydrolase | AGL65000.2 | 18.1 | 8.16 | 0.80 | 1.31E-04 |
| LBP_cg1329 | COG0144 [J] | tRNA/rRNA methyltransferase | AGL64075.2 | 50.3 | 8.57 | 0.80 | 3.89E-04 |
| LBP_cg0362 | COG0073 [R] | Methionyl-tRNA synthetase | AGL63108.2 | 76.7 | 5.35 | 0.80 | 1.86E-04 |
| LBP_cg0621 | COG2200 [T] | Diguanylate cyclase/phosphodiesterase domain-containing protein | AGL63367.2 | 25.2 | 5.54 | 0.80 | 4.69E-02 |
| LBP_cg2169 | COG1705 [NU] | Muramidase | AGL64915.2 | 82.1 | 9.00 | 0.79 | 2.92E-02 |
| LBP_cg0508 | COG0209 [F] | Ribonucleoside-diphosphate reductase | AGL63254.2 | 82.1 | 5.36 | 0.79 | 4.04E-04 |
| LBP_cg0323 | COG1174 [E] | Glycine betaine/carnitine/choline ABC transporter, substrate binding and permease protein | AGL63069.2 | 55.9 | 9.48 | 0.79 | 1.48E-03 |
| LBP_cg2467 | COG1396 [K] | Transcription regulator | AGL65213.2 | 30.5 | 7.11 | 0.79 | 1.09E-02 |
| LBP_cg0269 | COG0282 [C] | Acetate kinase | AGL63015.2 | 43.8 | 6.48 | 0.79 | 8.80E-03 |
| LBP_cg1957 | COG3442 [R] | Cobyric acid synthase (Putative) | AGL64703.2 | 26.5 | 5.30 | 0.79 | 1.46E-02 |
| LBP_cg0251 | COG0277 [C] | Lactate dehydrogenase (Oxidoreductase) | AGL62997.2 | 50.2 | 5.45 | 0.79 | 6.49E-03 |
| LBP_cg0215 | COG0105 [F] | Nucleoside-diphosphate kinase | AGL62961.2 | 17.0 | 8.84 | 0.79 | 2.73E-02 |
| LBP_cg1306 | - | hypothetical protein | AGL64052.2 | 9.5 | 9.45 | 0.79 | 1.77E-03 |
| LBP_cg1507 | - | hypothetical protein | AGL64253.2 | 63.9 | 5.30 | 0.79 | 4.79E-03 |
| LBP_cg2604 | COG1475 [K] | Chromosome partitioning protein, DNA-binding protein | AGL65350.2 | 31.7 | 9.00 | 0.79 | 6.13E-04 |
| LBP_cg2890 | COG0329 [EM] | N-acetylneuraminate lyase | AGL65636.2 | 32.7 | 4.91 | 0.78 | 4.03E-02 |
| LBP_cg0326 | COG0554 [C] | Glycerol kinase 1 | AGL63072.2 | 55.8 | 5.44 | 0.78 | 2.45E-02 |
| LBP_cg0717 | COG1835 [I] | Acyltransferase | AGL63463.2 | 66.7 | 9.00 | 0.78 | 2.45E-02 |
| LBP_cg2153 | - | hypothetical protein | AGL64899.2 | 32.2 | 6.74 | 0.78 | 6.40E-06 |
| LBP_cg1183 | COG0572 [F] | Uridine kinase | AGL63929.2 | 24.0 | 5.67 | 0.78 | 3.34E-02 |
| LBP_cg2650 | - | hypothetical protein | AGL65396.2 | 8.7 | 11.80 | 0.78 | 2.92E-03 |
| LBP_cg1247 | COG1196 [D] | Cell division protein Smc | AGL63993.2 | 131.8 | 5.55 | 0.78 | 3.30E-03 |
| LBP_cg2426 | COG1940 [KG] | Sugar kinase and transcription regulator | AGL65172.2 | 31.6 | 5.76 | 0.78 | 6.41E-03 |
| LBP_cg2185 | - | hypothetical protein | AGL64931.2 | 13.0 | 4.65 | 0.78 | 5.64E-03 |
| LBP_cg0551 | COG0642 [T] | Sensor protein | AGL63297.2 | 52.6 | 6.61 | 0.78 | 5.14E-03 |
| LBP_cg2729 | COG3189 [S] | Protein of hypothetical function DUF488 | AGL65475.2 | 14.4 | 7.40 | 0.78 | 1.39E-02 |
| LBP_cg1459 | COG0561 [R] | HAD superfamily hydrolase | AGL64205.2 | 28.9 | 5.01 | 0.78 | 8.81E-03 |
| LBP_cg1705 | COG1299 [G] | Fructose PTS, EIIABC | AGL64451.2 | 68.5 | 5.81 | 0.77 | 2.16E-04 |
| LBP_cg0231 | COG1887 [M] | Teichoic acid biosynthesis protein | AGL62977.2 | 47.0 | 9.07 | 0.77 | 1.01E-03 |
| LBP_cg2321 | COG1011 [R] | 2-haloacid dehalogenase (Putative) | AGL65067.2 | 25.3 | 5.76 | 0.77 | 6.85E-04 |
| LBP_cg2783 | - | hypothetical protein | AGL65529.2 | 14.5 | 10.17 | 0.77 | 5.17E-03 |
| LBP_cg0136 | COG1960 [I] | Butyryl-CoA dehydrogenase | AGL62882.2 | 53.9 | 5.07 | 0.77 | 3.81E-04 |
| LBP_cg1379 | COG0564 [J] | Pseudouridine synthase | AGL64125.2 | 33.8 | 9.39 | 0.77 | 6.42E-03 |
| LBP_cg0472 | COG0332 [I] | 3-oxoacyl-[acyl-carrier-protein | synthase 3 protein 1 | 34.4 | 5.01 | 0.76 | 9.34E-04 |
| LBP_cg0094 | COG0351 [H] | Phosphomethylpyrimidine kinase | AGL62840.2 | 28.3 | 5.54 | 0.76 | 6.31E-04 |
| LBP_cg2339 | COG0624 [E] | Succinyl-diaminopimelate desuccinylase | AGL65085.2 | 45.4 | 4.75 | 0.76 | 4.26E-05 |
| LBP_cg2835 | COG1609 [K] | Galactose operon repressor | AGL65581.2 | 37.1 | 7.65 | 0.76 | 1.60E-05 |
| LBP_cg1570 | COG1385 [S] | Protein of hypothetical function DUF558 | AGL64316.2 | 26.9 | 8.44 | 0.76 | 5.79E-04 |
| LBP_cg2799 | COG3590 [O] | Endopeptidase PepO | AGL65545.2 | 71.9 | 5.15 | 0.76 | 4.38E-04 |
| LBP_cg2665 | COG1929 [G] | Glycerate kinase | AGL65411.2 | 38.4 | 7.61 | 0.76 | 2.38E-03 |
| LBP_cg2278 | COG2188 [K] | Transcription regulator | AGL65024.2 | 26.9 | 7.21 | 0.76 | 9.69E-04 |
| LBP_cg2861 | COG0477 [GEPR] | Sugar transport protein | AGL65607.2 | 52.3 | 9.07 | 0.75 | 2.44E-04 |
| LBP_cg0368 | COG1658 [L] | Ribonuclease M5 | AGL63114.2 | 20.2 | 8.38 | 0.75 | 5.98E-03 |
| LBP_cg2911 | COG0028 [EH] | Pyruvate oxidase | AJF17183.1 | 66.1 | 5.57 | 0.75 | 4.88E-04 |
| LBP_cg2635 | COG1445 [G] | Protein-N(Pi)-phosphohistidine--sugar phosphotransferase | AGL65381.2 | 11.4 | 9.07 | 0.75 | 6.41E-03 |
| LBP_cg1959 | COG1680 [V] | putative beta-lactamase | AGL64705.2 | 38.0 | 9.60 | 0.75 | 8.42E-03 |
| LBP_cg0763 | - | hypothetical protein | AGL63509.2 | 37.8 | 9.42 | 0.75 | 8.44E-03 |
| LBP_cg2132 | COG1957 [F] | Purine nucleosidase | AGL64878.2 | 34.1 | 4.64 | 0.74 | 1.12E-02 |
| LBP_cg2496 | - | 1,3-propanediol dehydrogenase | AGL65242.2 | 42.1 | 5.64 | 0.74 | 6.13E-04 |
| LBP_cg1753 | COG4476 [S] | hypothetical protein | AGL64499.2 | 11.1 | 6.07 | 0.74 | 1.12E-03 |
| LBP_cg1079 | COG0744 [M] | Penicillin binding protein 2A | AGL63825.2 | 77.7 | 9.38 | 0.74 | 7.53E-04 |
| LBP_cg2094 | COG0446 [R] | NADH peroxidase | AGL64840.2 | 48.2 | 5.68 | 0.74 | 2.53E-03 |
| LBP_cg2233 | COG0026 [F] | Phosphoribosylaminoimidazole carboxylase ATPase subunit | AGL64979.2 | 40.5 | 6.47 | 0.73 | 1.13E-02 |
| LBP_cg0432 | COG1197 [LK] | Transcription-repair coupling factor | AGL63178.2 | 132.2 | 5.35 | 0.73 | 7.23E-05 |
| LBP_cg2497 | COG1062 [C] | Aryl-alcohol dehydrogenase | AGL65243.2 | 40.0 | 5.24 | 0.73 | 5.51E-03 |
| LBP_cg0600 | - | Cell surface protein | AGL63346.2 | 262.2 | 4.78 | 0.73 | 1.41E-02 |
| LBP_cg2230 | COG0047 [F] | Phosphoribosylformylglycinamidine synthase 1 | AGL64976.2 | 23.9 | 5.01 | 0.73 | 1.04E-02 |
| LBP_cg0324 | COG1125 [E] | ABC superfamily ATP binding cassette transporter, ABC protein | AGL63070.2 | 35.8 | 5.52 | 0.72 | 1.64E-04 |
| LBP_cg2282 | COG0561 [R] | HAD superfamily hydrolase | AGL65028.2 | 31.0 | 5.41 | 0.72 | 3.01E-05 |
| LBP_cg2900 | COG0753 [P] | Catalase | AJF17172.1 | 55.3 | 5.67 | 0.72 | 6.12E-04 |
| LBP_cg2706 | COG4989 [R] | Oxidoreductase | AGL65452.2 | 33.9 | 6.27 | 0.72 | 1.36E-02 |
| LBP_cg0148 | COG2182 [G] | Maltose/maltodextrin ABC transporter, substrate binding protein | AGL62894.2 | 45.6 | 9.69 | 0.72 | 3.65E-02 |
| LBP_cg0227 | COG1263 [G] | Phosphoenolpyruvate-dependent sugar PTS family porter EIIABC, trhalose specific | AGL62973.2 | 69.4 | 8.47 | 0.72 | 5.61E-03 |
| LBP_cg2335 | COG4221 [R] | Short-chain dehydrogenase/oxidoreductase | AGL65081.2 | 26.0 | 6.58 | 0.72 | 5.12E-03 |
| LBP_cg0570 | COG1109 [G] | Phosphoglucomutase | AGL63316.2 | 63.5 | 5.11 | 0.72 | 1.24E-05 |
| LBP_cg2159 | COG1073 [R] | S9 family serine peptidase | AGL64905.2 | 28.3 | 6.60 | 0.72 | 2.99E-05 |
| LBP_cg2489 | COG1473 [R] | Aminoacylase | AGL65235.2 | 43.0 | 5.99 | 0.71 | 1.71E-04 |
| LBP_cg2760 | COG0656 [R] | Oxidoreductase | AGL65506.2 | 31.7 | 5.67 | 0.71 | 4.84E-03 |
| LBP_cg0881 | COG1053 [C] | Succinate dehydrogenase | AGL63627.2 | 48.7 | 5.74 | 0.71 | 3.77E-05 |
| LBP_cg2750 | - | hypothetical protein | AGL65496.2 | 14.0 | 5.27 | 0.71 | 1.72E-06 |
| LBP_cg2691 | COG0285 [H] | Folylpolyglutamate synthase | AGL65437.2 | 49.3 | 7.20 | 0.71 | 3.24E-04 |
| LBP_cg1126 | COG0773 [M] | UDP-N-acetylmuramate--L-alanine ligase | AGL63872.2 | 48.7 | 5.67 | 0.70 | 2.56E-06 |
| LBP_cg2668 | COG0015 [F] | Adenylosuccinate lyase | AGL65414.2 | 49.0 | 5.97 | 0.70 | 2.97E-03 |
| LBP_cg0070 | - | Transcription regulator | AGL62816.2 | 11.9 | 9.25 | 0.70 | 1.97E-05 |
| LBP_cg2142 | COG1154 [HI] | 1-deoxy-D-xylulose-5-phosphate synthase | AGL64888.2 | 63.7 | 5.73 | 0.70 | 1.38E-03 |
| LBP_cg0108 | - | Transcription regulator | AGL62854.2 | 17.6 | 9.64 | 0.70 | 1.07E-04 |
| LBP_cg0555 | COG1117 [P] | Phosphate import ATP-binding protein pstB 1 | AGL63301.2 | 30.4 | 5.54 | 0.70 | 8.68E-04 |
| LBP_cg0647 | COG0596 [R] | Proline iminopeptidase | AGL63393.2 | 35.3 | 5.50 | 0.70 | 4.56E-06 |
| LBP_cg2832 | COG3345 [G] | Alpha-galactosidase | AGL65578.2 | 83.6 | 5.39 | 0.70 | 2.63E-02 |
| LBP_cg2666 | COG1249 [C] | Glutathione reductase | AGL65412.2 | 48.2 | 5.47 | 0.69 | 2.44E-02 |
| LBP_cg2921 | COG1609 [K] | Ribose operon repressor | AJF17193.1 | 36.8 | 6.30 | 0.69 | 2.13E-06 |
| LBP_cg0566 | COG0446 [R] | NADH oxidase | AGL63312.2 | 51.5 | 5.24 | 0.69 | 2.68E-03 |
| LBP_cg2258 | COG0498 [E] | Threonine synthase | AGL65004.2 | 54.5 | 6.35 | 0.69 | 6.45E-04 |
| LBP_cg2891 | COG3010 [G] | Putative N-acetylmannosamine-6-phosphate 2-epimerase | AGL65637.2 | 24.3 | 5.27 | 0.69 | 4.13E-05 |
| LBP_cg0153 | COG3839 [G] | Multiple sugar ABC transporter, ATP-binding protein | AGL62899.2 | 41.3 | 5.90 | 0.69 | 2.08E-02 |
| LBP_cg0095 | COG0352 [H] | Thiamine-phosphate pyrophosphorylase | AGL62841.2 | 22.8 | 5.14 | 0.69 | 1.58E-05 |
| LBP_cg0658 | COG0194 [F] | Guanylate kinase | AGL63404.2 | 21.7 | 6.89 | 0.68 | 2.81E-03 |
| LBP_cg1878 | - | Integral membrane protein | AGL64624.2 | 114.8 | 10.02 | 0.68 | 2.40E-03 |
| LBP_cg1274 | COG1063 [ER] | Alcohol dehydrogenase | AGL64020.2 | 36.8 | 6.07 | 0.68 | 4.24E-03 |
| LBP_cg0646 | COG0028 [EH] | Putative pyruvate oxidase | AGL63392.2 | 63.5 | 5.16 | 0.67 | 3.76E-05 |
| LBP_cg0119 | COG4716 [S] | hypothetical protein | AGL62865.2 | 64.2 | 5.59 | 0.66 | 7.27E-04 |
| LBP_cg1500 | COG0624 [E] | Succinyl-diaminopimelate desuccinylase | AGL64246.2 | 40.8 | 5.10 | 0.66 | 1.09E-03 |
| LBP_cg1454 | COG0488 [R] | ABC superfamily ATP binding cassette transporter, ABC protein | AGL64200.2 | 71.3 | 5.52 | 0.66 | 1.56E-02 |
| LBP_cg2524 | COG0367 [E] | Asparagine synthase (Glutamine-hydrolysing) | AGL65270.2 | 73.0 | 6.73 | 0.66 | 1.27E-02 |
| LBP_cg1333 | COG2017 [G] | Aldose 1-epimerase | AGL64079.2 | 36.6 | 6.06 | 0.66 | 1.74E-02 |
| LBP_cg2901 | COG1393 [P] | Negative regulator of proteolysis | AJF17173.1 | 14.5 | 7.50 | 0.66 | 6.49E-04 |
| LBP_cg0469 | COG1221 [KT] | Transcription regulator | AGL63215.2 | 105.2 | 5.67 | 0.66 | 2.69E-04 |
| LBP_cg1563 | COG0124 [J] | Histidyl-tRNA synthetase | AGL64309.2 | 47.9 | 5.35 | 0.66 | 2.30E-04 |
| LBP_cg1627 | COG0463 [M] | Glycosyltransferase | AGL64373.2 | 35.5 | 6.09 | 0.65 | 2.35E-05 |
| LBP_cg2234 | COG0041 [F] | Phosphoribosylaminoimidazole carboxylase, catalytic subunit | AGL64980.2 | 16.9 | 7.06 | 0.65 | 2.69E-02 |
| LBP_cg2168 | COG0095 [H] | Lipoate-protein ligase | AGL64914.2 | 38.2 | 5.10 | 0.64 | 9.03E-04 |
| LBP_cg2946 | COG1847 [R] | hypothetical protein | AJF17218.1 | 34.2 | 9.73 | 0.64 | 8.69E-05 |
| LBP_cg0556 | COG1117 [P] | Phosphate import ATP-binding protein pstB 2 | AGL63302.2 | 28.0 | 5.34 | 0.64 | 1.25E-04 |
| LBP_p1g002 | COG1418 [R] | phosphohydrolase | AGL65641.2 | 25.3 | 6.67 | 0.64 | 1.16E-03 |
| LBP_cg2605 | COG0357 [M] | Ribosomal RNA small subunit methyltransferase G | AGL65351.2 | 26.8 | 9.22 | 0.63 | 4.21E-04 |
| LBP_cg2670 | COG0516 [F] | GMP reductase | AGL65416.2 | 35.4 | 6.87 | 0.63 | 1.70E-03 |
| LBP_cg2375 | COG1511 [S] | Integral membrane protein | AGL65121.2 | 126.8 | 9.07 | 0.63 | 4.90E-03 |
| LBP_cg1331 | COG0477 [GEPR] | Major facilitator superfamily permease | AGL64077.2 | 48.3 | 9.50 | 0.63 | 8.85E-03 |
| LBP_cg0610 | COG0708 [L] | Exodeoxyribonuclease III | AGL63356.2 | 29.6 | 5.34 | 0.63 | 9.13E-04 |
| LBP_cg2555 | COG1455 [G] | Cellobiose PTS, EIIC | AGL65301.2 | 49.0 | 8.66 | 0.62 | 3.52E-05 |
| LBP_cg1356 | COG0116 [L] | Site-specific DNA-methyltransferase | AGL64102.2 | 43.1 | 6.83 | 0.62 | 3.91E-05 |
| LBP_cg0983 | COG0503 [F] | Purine/pyrimidine phosphoribosyltransferase (Putative) | AGL63729.2 | 19.6 | 6.95 | 0.62 | 3.77E-02 |
| LBP_cg1374 | COG0665 [E] | Oxidoreductase | AGL64120.2 | 40.4 | 5.95 | 0.62 | 1.95E-04 |
| LBP_cg0154 | COG1554 [G] | Maltose phosphorylase | AGL62900.2 | 86.5 | 5.48 | 0.61 | 1.02E-02 |
| LBP_cg1332 | COG1554 [G] | Maltose phosphorylase | AGL64078.2 | 85.7 | 5.03 | 0.61 | 1.26E-02 |
| LBP_cg2495 | - | Extracellular protein | AGL65241.2 | 15.0 | 10.45 | 0.61 | 1.65E-02 |
| LBP_cg2681 | COG0620 [E] | hypothetical protein | AGL65427.2 | 42.1 | 5.91 | 0.60 | 5.25E-06 |
| LBP_cg0954 | COG1023 [G] | 6-phosphogluconate dehydrogenase, decarboxylating | AGL63700.2 | 31.8 | 5.52 | 0.60 | 4.90E-04 |
| LBP_p2g010 | COG2190 [G] | GPH family glycoside-pentoside-hexuronide:cation symporter | AGL65706.2 | 69.8 | 7.64 | 0.59 | 7.37E-03 |
| LBP_cg2311 | COG1136 [V] | ABC transporter, ATP-binding protein | AGL65057.2 | 24.7 | 6.40 | 0.59 | 2.74E-03 |
| LBP_cg2231 | COG1828 [F] | Purine biosynthesis cluster protein | AGL64977.2 | 9.7 | 4.88 | 0.59 | 4.79E-03 |
| LBP_cg2612 | COG1167 [KE] | putative Aromatic-amino-acid transaminase | AGL65358.2 | 45.3 | 6.24 | 0.59 | 3.87E-02 |
| LBP_cg2225 | COG0138 [F] | Bifunctional phosphoribosylaminoimidazolecarboxamide formyltransferase/IMP cyclohydrolase | AGL64971.2 | 55.3 | 6.13 | 0.58 | 6.28E-03 |
| LBP_cg2226 | COG0299 [F] | Phosphoribosylglycinamide formyltransferase | AGL64972.2 | 20.6 | 6.11 | 0.58 | 3.34E-02 |
| LBP_cg1497 | COG0148 [G] | Enolase | AGL64243.2 | 46.6 | 5.03 | 0.57 | 1.95E-05 |
| LBP_cg2472 | COG2723 [G] | 6-phospho-beta-glucosidase | AGL65218.2 | 53.4 | 5.21 | 0.57 | 1.21E-04 |
| LBP_cg1720 | COG0765 [E] | Glutamine ABC transporter, substrate binding and permease protein | AGL64466.2 | 53.8 | 9.82 | 0.57 | 8.27E-03 |
| LBP_cg1719 | COG1126 [E] | Glutamine ABC transporter, ATP-binding protein | AGL64465.2 | 26.9 | 4.86 | 0.56 | 7.86E-03 |
| LBP_p2g050 | COG1249 [C] | Pyridine nucleotide-disulfide oxidoreductase family protein | AJF17232.1 | 50.9 | 8.69 | 0.56 | 4.95E-05 |
| LBP_cg0179 | COG0667 [C] | Aryl-alcohol dehydrogenase family enzyme | AGL62925.2 | 37.0 | 7.03 | 0.55 | 1.79E-03 |
| LBP_cg2856 | COG2723 [G] | 6-phospho-beta-glucosidase | AGL65602.2 | 55.2 | 5.21 | 0.54 | 1.65E-03 |
| LBP_cg2669 | COG0104 [F] | Adenylosuccinate synthetase | AGL65415.2 | 47.2 | 5.64 | 0.54 | 4.52E-03 |
| LBP_cg0426 | COG0078 [E] | Ornithine carbamoyltransferase | AGL63172.2 | 36.8 | 5.60 | 0.54 | 1.30E-02 |
| LBP_cg2224 | COG0151 [F] | Phosphoribosylamine--glycine ligase | AGL64970.2 | 43.5 | 5.59 | 0.54 | 4.38E-02 |
| LBP_cg2156 | COG0028 [EH] | Pyruvate oxidase | AGL64902.2 | 64.2 | 5.12 | 0.52 | 5.28E-03 |
| LBP_cg0152 | COG0366 [G] | Alpha-amylase | AGL62898.2 | 49.8 | 5.08 | 0.51 | 1.91E-02 |
| LBP_cg2227 | COG0150 [F] | Phosphoribosylformylglycinamidine cyclo-ligase | AGL64973.2 | 35.8 | 5.11 | 0.51 | 2.84E-03 |
| LBP_cg2554 | COG2723 [G] | 6-phospho-beta-glucosidase | AGL65300.2 | 54.3 | 5.41 | 0.50 | 1.57E-03 |
| LBP_cg2229 | COG0046 [F] | Phosphoribosylformylglycinamidine synthase 2 | AGL64975.2 | 79.7 | 5.34 | 0.49 | 8.83E-03 |
| LBP_p2g025 | COG1307 [S] | hypothetical protein | AGL65721.2 | 30.6 | 9.23 | 0.49 | 2.22E-05 |
| LBP_cg0974 | COG1080 [G] | Phosphoenolpyruvate-protein phosphatase | AGL63720.2 | 63.1 | 4.83 | 0.48 | 2.67E-04 |
| LBP_cg0158 | COG1940 [KG] | Fructokinase | AGL62904.2 | 30.6 | 5.19 | 0.47 | 2.70E-05 |
| LBP_cg2232 | COG0152 [F] | Phosphoribosylaminoimidazole-succinocarboxamide synthase | AGL64978.2 | 27.1 | 6.24 | 0.45 | 5.76E-03 |
| LBP_cg0580 | COG0165 [E] | Argininosuccinate lyase | AGL63326.2 | 52.3 | 5.69 | 0.42 | 6.60E-03 |
| LBP_p2g047 | - | hypothetical protein | AJF17229.1 | 7.9 | 9.99 | 0.41 | 8.74E-05 |
| LBP_p2g004 | COG3250 [G] | Beta-galactosidase | AGL65700.1 | 35.2 | 4.86 | 0.40 | 8.01E-04 |
| LBP_p1g033 | COG1192 [D] | Copy number control protein | AGL65672.2 | 30.1 | 5.43 | 0.40 | 9.26E-06 |
| LBP_cg0579 | COG0137 [E] | Argininosuccinate synthase | AGL63325.2 | 45.1 | 5.14 | 0.39 | 1.88E-02 |
| LBP_cg2862 | COG1501 [G] | Alpha-glucosidase | AGL65608.2 | 87.2 | 6.24 | 0.39 | 6.57E-06 |
| LBP_p1g016 | COG1961 [L] | Resolvase | AGL65655.2 | 22.1 | 9.99 | 0.37 | 2.01E-04 |
| LBP_cg2228 | COG0034 [F] | Amidophosphoribosyltransferase | AGL64974.2 | 52.3 | 6.04 | 0.35 | 8.87E-03 |
| LBP_cg1351 | COG0744 [M] | Penicillin binding protein 1A | AGL64097.2 | 83.0 | 9.39 | 0.35 | 9.02E-06 |
| LBP_p1g047 | COG2190 [G] | PTS sugar transporter subunit IIA | AGL65686.2 | 71.5 | 8.15 | 0.21 | 1.26E-03 |
| LBP_p1g007 | COG0662 [G] | Cupin 2 conserved barrel domain protein | AGL65646.2 | 16.2 | 5.47 | 0.17 | 3.64E-03 |
