# Supplementary material for: Adaptation of Lactobacillus plantarum to Ampicillin Involves Mechanisms That Maintain Protein Homeostasis
Source: mSystems. 2020 Jan 28;5(1):e00853-19. doi: 10.1128/mSystems.00853-19 (PMC6989132; doi:10.1128/mSystems.00853-19)
Supplement: TABLE S5 [file mSystems.00853-19-st005.docx]

**Table S5**. Up-regulated proteins of *L. plantarum* 1600g relative to *L. plantarum* P-8 grown in the presence of the ampicillin

| **Accession** | **COG category** | **Description** | **Protein ID** | **MW [kDa]** | **calc. pI** | **Fold change** | **T test p value** |
| --- | --- | --- | --- | --- | --- | --- | --- |
| LBP_p3g026 | - | hypothetical protein | AGL65764.2 | 11.1 | 9.73 | 3.97 | 1.25E-05 |
| LBP_cg2704 | COG1180 [O] | Formate acetyltransferase activating enzyme | AGL65450.2 | 31.3 | 6.46 | 3.95 | 3.96E-06 |
| LBP_cg0397 | COG1760 [E] | L-serine dehydratase, beta subunit | AGL63143.2 | 23.8 | 5.95 | 3.32 | 3.92E-04 |
| LBP_cg1489 | COG0574 [G] | Pyruvate,water dikinase | AGL64235.2 | 88.1 | 5.62 | 3.31 | 4.84E-06 |
| LBP_cg2703 | COG1882 [C] | Formate C-acetyltransferase | AGL65449.2 | 84.4 | 5.57 | 3.26 | 4.88E-06 |
| LBP_cg2927 | COG1012 [C] | Bifunctional acetaldehyde-CoA/alcohol dehydrogenase | AJF17199.1 | 94.5 | 6.79 | 3.01 | 8.16E-05 |
| LBP_cg0288 | COG1012 [C] | Acetaldehyde dehydrogenase | AGL63034.2 | 48.7 | 6.80 | 2.84 | 5.94E-04 |
| LBP_cg0398 | COG1760 [E] | L-serine dehydratase, alpha subunit | AGL63144.2 | 30.7 | 5.68 | 2.83 | 1.04E-04 |
| LBP_cg0644 | COG0028 [EH] | Pyruvate oxidase | AGL63390.2 | 63.5 | 5.35 | 2.77 | 1.68E-05 |
| LBP_cg2450 | - | hypothetical protein | AGL65196.2 | 8.2 | 4.56 | 2.52 | 1.98E-03 |
| LBP_cg1450 | COG2755 [E] | SGNH superfamily hydrolase | AGL64196.2 | 35.8 | 9.86 | 2.51 | 4.85E-04 |
| LBP_cg0880 | COG0114 [C] | Fumarate hydratase | AGL63626.2 | 49.5 | 5.16 | 2.48 | 2.15E-04 |
| LBP_cg0872 | COG0471 [P] | Cation transport protein | AGL63618.2 | 50.6 | 9.10 | 2.43 | 5.87E-05 |
| LBP_cg1738 | COG3480 [T] | Endopeptidase La (Putative) | AGL64484.2 | 38.5 | 10.05 | 2.36 | 6.20E-05 |
| LBP_cg2434 | COG0778 [C] | Nitroreductase | AGL65180.2 | 24.1 | 5.43 | 2.32 | 3.72E-05 |
| LBP_cg2842 | COG0169 [E] | Shikimate 5-dehydrogenase | AGL65588.2 | 32.6 | 5.11 | 2.23 | 5.29E-05 |
| LBP_cg0842 | COG4939 [S] | Lipoprotein | AGL63588.2 | 32.9 | 9.76 | 2.18 | 7.69E-05 |
| LBP_p6g001 | COG3409 [M] | YkuG protein | AGL65828.2 | 83.1 | 5.55 | 2.14 | 5.19E-08 |
| LBP_cg0396 | COG0814 [E] | Serine transporter | AGL63142.2 | 46.9 | 9.39 | 2.06 | 3.35E-05 |
| LBP_cg0871 | COG0039 [C] | L-lactate dehydrogenase 2 | AGL63617.2 | 33.1 | 5.54 | 2.04 | 6.52E-05 |
| LBP_cg1908 | COG1104 [E] | Cysteine desulfurase | AGL64654.2 | 42.3 | 7.25 | 1.93 | 9.27E-05 |
| LBP_cg2458 | - | hypothetical protein | AGL65204.2 | 10.3 | 5.03 | 1.86 | 2.14E-02 |
| LBP_cg1907 | COG0301 [H] | putative tRNA sulfurtransferase | AGL64653.2 | 45.5 | 6.39 | 1.86 | 4.56E-05 |
| LBP_cg0964 | COG0601 [EP] | Oligopeptide ABC superfamily ATP binding cassette transporter, permease protein | AGL63710.2 | 33.7 | 9.11 | 1.84 | 4.09E-04 |
| LBP_cg0780 | - | hypothetical protein | AGL63526.2 | 26.8 | 10.48 | 1.82 | 4.37E-04 |
| LBP_cg0720 | - | hypothetical protein | AGL63466.2 | 6.4 | 8.68 | 1.79 | 9.04E-03 |
| LBP_cg0504 | - | Prophage Lp1 protein 66, lipoprotein | AGL63250.2 | 23.2 | 10.68 | 1.77 | 4.82E-04 |
| LBP_p3g011 | - | Peptidoglycan-binding protein | AGL65749.2 | 31.3 | 9.50 | 1.76 | 4.69E-04 |
| LBP_cg2610 | COG4814 [R] | hypothetical protein | AGL65356.2 | 30.8 | 11.12 | 1.72 | 1.48E-05 |
| LBP_cg1330 | - | hypothetical protein | AGL64076.2 | 30.3 | 5.60 | 1.71 | 9.08E-03 |
| LBP_cg2912 | COG3731 [G] | Sorbitol PTS, EIIA | AJF17184.1 | 13.5 | 4.79 | 1.68 | 1.40E-02 |
| LBP_cg2778 | COG1866 [C] | Phosphoenolpyruvate carboxykinase (ATP) | AGL65524.2 | 61.8 | 6.32 | 1.67 | 2.46E-03 |
| LBP_cg0399 | COG2964 [S] | hypothetical protein | AGL63145.2 | 27.1 | 5.99 | 1.67 | 7.92E-04 |
| LBP_cg1116 | COG0760 [O] | Foldase protein prsA 1 | AGL63862.2 | 32.6 | 9.77 | 1.67 | 1.22E-03 |
| LBP_cg1038 | - | hypothetical protein | AGL63784.2 | 10.6 | 4.81 | 1.65 | 3.72E-04 |
| LBP_cg0921 | COG1278 [K] | Cold shock protein 1 | AGL63667.2 | 7.3 | 4.65 | 1.64 | 4.94E-02 |
| LBP_cg1575 | - | hypothetical protein | AGL64321.2 | 32.9 | 5.44 | 1.64 | 6.48E-04 |
| LBP_p3g031 | COG0550 [L] | DNA topoisomerase | AGL65769.1 | 73.8 | 9.09 | 1.63 | 6.71E-04 |
| LBP_cg2913 | COG3732 [G] | Sorbitol PTS, EIIBC | AJF17185.1 | 35.7 | 6.07 | 1.61 | 5.01E-03 |
| LBP_cg0395 | COG0172 [J] | Seryl-tRNA synthetase 1 | AGL63141.2 | 47.8 | 5.64 | 1.61 | 1.62E-03 |
| LBP_cg2803 | COG0446 [R] | NADH oxidase | AGL65549.2 | 49.3 | 5.25 | 1.58 | 2.39E-03 |
| LBP_cg0722 | COG1302 [S] | Alkaline shock protein | AGL63468.2 | 16.1 | 4.94 | 1.57 | 2.68E-03 |
| LBP_cg0537 | COG0234 [O] | 10 kDa chaperonin | AGL63283.2 | 10.3 | 5.03 | 1.56 | 3.10E-02 |
| LBP_cg2917 | COG1028 [IQR] | Sorbitol-6-phosphate 2-dehydrogenase | AJF17189.1 | 28.6 | 6.70 | 1.55 | 3.98E-03 |
| LBP_cg0877 | COG2301 [G] | Citrate lyase, beta chain | AGL63623.2 | 33.2 | 5.21 | 1.55 | 1.56E-05 |
| LBP_cg0878 | COG3051 [C] | Citrate lyase, alpha chain | AGL63624.2 | 54.9 | 5.63 | 1.54 | 8.67E-06 |
| LBP_cg2936 | - | Transcription regulator | AJF17208.1 | 15.5 | 10.35 | 1.54 | 3.10E-03 |
| LBP_cg2937 | COG3579 [E] | Cysteine aminopeptidase | AJF17209.1 | 49.9 | 5.24 | 1.53 | 2.36E-04 |
| LBP_cg2240 | COG4814 [R] | Cell surface hydrolase, membrane-bound | AGL64986.2 | 29.6 | 9.04 | 1.51 | 2.14E-02 |
| LBP_cg1866 | COG4472 [S] | hypothetical protein | AGL64612.2 | 10.3 | 5.06 | 1.51 | 2.94E-03 |
| LBP_cg0386 | - | Lipoprotein | AGL63132.2 | 34.3 | 6.00 | 1.49 | 5.80E-03 |
| LBP_cg0876 | COG3052 [C] | Citrate lyase acyl carrier protein | AGL63622.2 | 10.4 | 5.10 | 1.49 | 5.36E-05 |
| LBP_cg2497 | COG1062 [C] | Aryl-alcohol dehydrogenase | AGL65243.2 | 40.0 | 5.24 | 1.48 | 4.95E-04 |
| LBP_cg0225 | COG2188 [K] | GntR family transcriptional regulator | AGL62971.2 | 26.9 | 7.05 | 1.48 | 1.12E-03 |
| LBP_cg1441 | COG0620 [E] | putative 5-methyltetrahydropteroyltriglutamate--homocysteine S-methyltransferase | AGL64187.2 | 43.2 | 5.33 | 1.48 | 9.32E-04 |
| LBP_cg1564 | COG0860 [M] | N-acetylmuramoyl-L-alanine amidase | AGL64310.2 | 31.0 | 9.66 | 1.44 | 5.13E-04 |
| LBP_cg1218 | COG1722 [L] | Exodeoxyribonuclease 7 small subunit | AGL63964.2 | 8.4 | 4.01 | 1.44 | 6.85E-03 |
| LBP_cg2779 | - | hypothetical protein | AGL65525.2 | 7.4 | 6.28 | 1.44 | 2.51E-02 |
| LBP_cg0841 | COG1252 [C] | NADH dehydrogenase | AGL63587.2 | 71.8 | 8.85 | 1.43 | 5.88E-04 |
| LBP_cg0569 | - | hypothetical protein | AGL63315.2 | 17.3 | 9.70 | 1.43 | 9.48E-04 |
| LBP_cg2519 | - | Extracellular protein (Putative) | AGL65265.2 | 29.3 | 10.62 | 1.42 | 1.92E-02 |
| LBP_p5g002 | - | putative cell surface protein | AGL65811.2 | 67.0 | 9.85 | 1.42 | 1.93E-02 |
| LBP_cg1046 | COG0620 [E] | 5-methyltetrahydropteroyltriglutamate--homocysteine methyltransferase | AGL63792.2 | 86.4 | 6.11 | 1.41 | 1.80E-04 |
| LBP_cg0965 | COG1173 [EP] | Oligopeptide ABC transporter, permease protein | AGL63711.2 | 37.6 | 9.31 | 1.40 | 8.21E-04 |
| LBP_cg0797 | COG1876 [M] | Serine-type D-Ala-D-Ala carboxypeptidase | AGL63543.2 | 26.9 | 9.61 | 1.39 | 1.43E-04 |
| LBP_cg2597 | COG0760 [O] | Peptidylprolyl isomerase | AGL65343.2 | 34.3 | 9.98 | 1.39 | 1.27E-02 |
| LBP_cg2150 | COG1073 [R] | Cell surface hydrolase, membrane-bound (Putative) | AGL64896.2 | 34.2 | 9.98 | 1.38 | 5.87E-04 |
| LBP_cg2620 | - | hypothetical protein | AGL65366.2 | 33.4 | 8.40 | 1.38 | 1.81E-03 |
| LBP_p3g028 | COG0507 [L] | Nickase | AGL65766.1 | 80.7 | 8.66 | 1.38 | 1.87E-03 |
| LBP_cg0694 | COG2070 [R] | 2-nitropropane dioxygenase | AGL63440.2 | 32.1 | 5.44 | 1.37 | 2.68E-04 |
| LBP_cg1196 | - | hypothetical protein | AGL63942.2 | 6.4 | 10.11 | 1.37 | 2.00E-03 |
| LBP_cg2289 | - | Cell surface protein | AGL65035.2 | 113.1 | 4.48 | 1.37 | 1.40E-03 |
| LBP_cg2235 | COG0431 [R] | Oxidoreductase (Putative) | AGL64981.2 | 47.2 | 5.25 | 1.37 | 7.51E-04 |
| LBP_cg2768 | COG4086 [S] | Extracellular protein | AGL65514.2 | 34.2 | 9.51 | 1.37 | 5.24E-05 |
| LBP_cg2330 | - | Extracellular protein | AGL65076.2 | 33.2 | 9.55 | 1.36 | 8.82E-03 |
| LBP_p3g034 | - | hypothetical protein | AGL65772.2 | 16.9 | 9.69 | 1.36 | 1.27E-02 |
| LBP_cg2840 | COG0710 [E] | 3-dehydroquinate dehydratase | AGL65586.2 | 27.2 | 5.00 | 1.36 | 2.54E-05 |
| LBP_cg2288 | COG0716 [C] | Flavodoxin | AGL65034.2 | 16.0 | 4.13 | 1.36 | 1.13E-02 |
| LBP_cg1750 | - | Extracellular protein | AGL64496.2 | 42.1 | 6.57 | 1.35 | 7.79E-03 |
| LBP_cg1037 | - | hypothetical protein | AGL63783.2 | 19.4 | 9.67 | 1.34 | 4.59E-04 |
| LBP_p6g011 | COG0783 [P] | Stress induced DNA binding protein | AJF17241.1 | 18.0 | 4.79 | 1.34 | 4.75E-03 |
| LBP_cg0896 | - | hypothetical protein | AGL63642.2 | 36.9 | 10.32 | 1.34 | 1.96E-02 |
| LBP_cg2286 | COG2085 [R] | Oxidoreductase | AGL65032.2 | 20.4 | 5.59 | 1.34 | 1.27E-03 |
| LBP_cg2236 | COG0431 [R] | Oxidoreductase (Putative) | AGL64982.2 | 22.8 | 5.27 | 1.34 | 3.57E-04 |
| LBP_cg2828 | COG1087 [M] | UDP-glucose 4-epimerase | AGL65574.2 | 36.4 | 5.53 | 1.34 | 2.89E-03 |
| LBP_cg1808 | COG0199 [J] | 30S ribosomal protein S14 | AGL64554.2 | 10.1 | 11.43 | 1.34 | 1.90E-02 |
| LBP_cg1793 | COG0768 [M] | Penicillin binding protein 2B | AGL64539.2 | 77.2 | 9.85 | 1.34 | 3.49E-03 |
| LBP_cg1077 | COG1438 [K] | Arginine regulator | AGL63823.2 | 17.1 | 5.06 | 1.34 | 2.64E-03 |
| LBP_cg2780 | COG0076 [E] | Glutamate decarboxylase | AGL65526.2 | 53.6 | 6.00 | 1.33 | 4.77E-02 |
| LBP_cg1165 | COG3212 [S] | Lipoprotein | AGL63911.2 | 21.0 | 5.59 | 1.33 | 1.79E-04 |
| LBP_cg0614 | - | Transcription regulator | AGL63360.2 | 19.8 | 8.32 | 1.33 | 3.71E-04 |
| LBP_cg0649 | COG1835 [I] | Acyltransferase (Putative) | AGL63395.2 | 74.3 | 9.89 | 1.33 | 6.02E-03 |
| LBP_cg0753 | COG0017 [J] | Asparaginyl-tRNA synthetase | AGL63499.2 | 49.0 | 5.24 | 1.33 | 4.64E-04 |
| LBP_cg1963 | - | Lipoprotein | AGL64709.2 | 34.5 | 10.49 | 1.33 | 8.30E-03 |
| LBP_cg0785 | COG1278 [K] | Cold shock protein CspC | AGL63531.2 | 7.3 | 4.70 | 1.33 | 4.66E-02 |
| LBP_cg2905 | COG0542 [O] | ATP-dependent Clp protease, ATP-binding subunit ClpL | AJF17177.1 | 77.8 | 5.63 | 1.32 | 1.00E-03 |
| LBP_cg0104 | COG0474 [P] | Cation transporting P-type ATPase | AGL62850.2 | 99.6 | 4.93 | 1.32 | 3.68E-03 |
| LBP_cg2422 | COG1835 [I] | putative acyltransferase | AGL65168.2 | 25.5 | 9.00 | 1.32 | 7.47E-04 |
| LBP_cg0226 | COG0366 [G] | Alpha, alpha-phosphotrehalase | AGL62972.2 | 62.0 | 5.91 | 1.32 | 7.40E-04 |
| LBP_cg0844 | COG1477 [H] | Thiamin biosynthesis lipoprotein ApbE | AGL63590.2 | 40.2 | 9.73 | 1.32 | 6.04E-04 |
| LBP_cg1317 | COG4690 [E] | Dipeptidase | AGL64063.2 | 53.4 | 5.12 | 1.32 | 1.12E-02 |
| LBP_p3g024 | - | DNA-damage-inducible protein | AGL65762.1 | 10.4 | 4.74 | 1.32 | 1.25E-03 |
| LBP_cg2781 | COG0791 [M] | Extracellular protein, gamma-D-glutamate-meso-diaminopimelate muropeptidase (Putative) | AGL65527.2 | 34.7 | 9.39 | 1.31 | 1.97E-02 |
| LBP_p5g005 | COG0474 [P] | Cation-transporting ATPase, E1-E2 family | AGL65823.2 | 100.9 | 5.74 | 1.31 | 8.91E-04 |
| LBP_cg0788 | COG1316 [K] | Transcription regulator | AGL63534.2 | 37.7 | 9.89 | 1.31 | 1.59E-03 |
| LBP_cg0587 | COG0740 [OU] | ATP-dependent Clp protease proteolytic subunit | AGL63333.2 | 21.5 | 5.02 | 1.31 | 6.81E-03 |
| LBP_cg1294 | COG1607 [I] | Acyl-CoA thioester hydrolase (Putative) | AGL64040.2 | 18.6 | 6.28 | 1.30 | 7.63E-03 |
| LBP_cg1017 | COG1970 [M] | Large-conductance mechanosensitive channel | AGL63763.2 | 14.0 | 4.94 | 1.29 | 3.76E-02 |
| LBP_cg1822 | COG2329 [R] | hypothetical protein | AGL64568.2 | 19.2 | 8.76 | 1.29 | 2.66E-03 |
| LBP_cg1928 | COG1464 [P] | ABC superfamily ATP binding cassette transporter, binding protein | AGL64674.2 | 29.9 | 9.85 | 1.29 | 5.10E-05 |
| LBP_cg0949 | COG0039 [C] | L-2-hydroxyisocaproate dehydrogenase | AGL63695.2 | 33.3 | 5.66 | 1.28 | 1.31E-02 |
| LBP_cg1703 | COG1349 [KG] | Transcription regulator of fructose operon | AGL64449.2 | 27.7 | 5.81 | 1.28 | 4.60E-02 |
| LBP_cg0721 | COG1302 [S] | Alkaline shock protein | AGL63467.2 | 15.8 | 5.06 | 1.28 | 2.58E-03 |
| LBP_cg1858 | COG0127 [F] | Nucleoside-triphosphatase | AGL64604.2 | 21.8 | 6.96 | 1.28 | 3.27E-04 |
| LBP_cg0538 | COG0459 [O] | 60 kDa chaperonin | AGL63284.2 | 57.4 | 4.81 | 1.27 | 1.63E-02 |
| LBP_cg1611 | COG0233 [J] | Ribosome-recycling factor | AGL64357.2 | 20.6 | 6.11 | 1.27 | 3.15E-02 |
| LBP_cg0430 | COG0039 [C] | L-lactate dehydrogenase 1 | AGL63176.2 | 34.2 | 5.05 | 1.27 | 4.07E-02 |
| LBP_cg2475 | - | Extracellular protein | AGL65221.2 | 21.3 | 8.91 | 1.27 | 4.96E-02 |
| LBP_cg0237 | COG1914 [P] | putative manganese transport protein mntH | AGL62983.2 | 50.3 | 9.60 | 1.27 | 2.16E-04 |
| LBP_cg0477 | COG1316 [K] | Transcription regulator | AGL63223.2 | 43.6 | 9.79 | 1.27 | 1.93E-03 |
| LBP_cg1962 | - | hypothetical protein | AGL64708.2 | 12.9 | 5.66 | 1.27 | 2.77E-03 |
| LBP_cg0995 | COG0192 [H] | S-adenosylmethionine synthetase | AGL63741.2 | 42.6 | 4.94 | 1.26 | 4.52E-05 |
| LBP_cg0575 | COG1896 [R] | HD superfamily hydrolase | AGL63321.2 | 24.5 | 5.43 | 1.26 | 2.23E-05 |
| LBP_cg0590 | COG0057 [G] | Glyceraldehyde 3-phosphate dehydrogenase | AGL63336.2 | 36.4 | 5.54 | 1.26 | 3.10E-02 |
| LBP_cg1931 | COG0509 [E] | Glycine cleavage system, H protein | AGL64677.2 | 10.5 | 4.41 | 1.25 | 4.26E-02 |
| LBP_cg0217 | COG0431 [R] | putative NAD(P)H dehydrogenase (Quinone) | AGL62963.2 | 24.6 | 6.70 | 1.25 | 7.46E-03 |
| LBP_cg0758 | - | hypothetical protein | AGL63504.2 | 7.8 | 9.70 | 1.25 | 4.72E-02 |
| LBP_cg1341 | COG0017 [J] | Asparaginyl-tRNA synthetase 2 | AGL64087.2 | 49.8 | 5.11 | 1.25 | 1.00E-03 |
| LBP_cg1786 | COG1799 [S] | Cell division protein sepF | AGL64532.2 | 15.3 | 4.54 | 1.25 | 6.67E-04 |
| LBP_cg1680 | COG3646 [S] | Phage anti-repressor protein | AGL64426.2 | 26.8 | 9.17 | 1.25 | 4.88E-02 |
| LBP_cg0967 | COG4608 [E] | ABC superfamily ATP binding cassette transporter, ABC protein | AGL63713.2 | 36.6 | 6.02 | 1.24 | 4.08E-04 |
| LBP_p5g014 | - | hypothetical protein | AGL65826.2 | 10.7 | 4.86 | 1.24 | 4.94E-03 |
| LBP_cg1232 | COG0223 [J] | Methionyl-tRNA formyltransferase | AGL63978.2 | 34.4 | 7.06 | 1.24 | 1.19E-04 |
| LBP_cg2118 | - | hypothetical protein | AGL64864.2 | 8.1 | 6.10 | 1.24 | 9.74E-03 |
| LBP_cg2248 | COG0589 [T] | Universal stress protein UspA | AGL64994.2 | 17.1 | 4.94 | 1.24 | 1.63E-03 |
| LBP_cg0519 | COG4467 [S] | Initiation-control protein yabA | AGL63265.2 | 13.6 | 5.00 | 1.23 | 1.04E-02 |
| LBP_cg1033 | - | Extracellular protein, membrane-anchored (Putative) | AGL63779.2 | 12.5 | 10.77 | 1.23 | 4.40E-02 |
| LBP_cg2271 | COG0842 [V] | ABC superfamily ATP binding cassette transporter, permease protein | AGL65017.2 | 43.5 | 9.95 | 1.23 | 3.67E-02 |
| LBP_cg0482 | COG3579 [E] | Cysteine aminopeptidase | AGL63228.2 | 50.2 | 5.06 | 1.23 | 1.02E-03 |
| LBP_cg1862 | COG1193 [L] | MutS2 protein | AGL64608.2 | 87.2 | 6.58 | 1.23 | 2.85E-04 |
| LBP_cg0394 | COG0504 [F] | CTP synthase | AGL63140.2 | 59.7 | 5.69 | 1.23 | 8.70E-06 |
| LBP_cg2291 | - | cell surface protein precursor | AGL65037.2 | 64.6 | 4.58 | 1.23 | 6.33E-04 |
| LBP_cg2310 | COG0577 [V] | ABC transporter, permease protein (Putative) | AGL65056.2 | 38.1 | 9.94 | 1.23 | 1.10E-02 |
| LBP_cg0838 | COG0142 [H] | Trans-hexaprenyltranstransferase, component II | AGL63584.2 | 37.1 | 6.01 | 1.22 | 7.42E-03 |
| LBP_p5g013 | COG0582 [L] | Tyrosine recombinase | AGL65825.2 | 22.6 | 9.20 | 1.22 | 2.80E-03 |
| LBP_cg0357 | COG0846 [K] | NAD-dependent deacetylase (Regulatory protein SIR2 family protein) | AGL63103.2 | 26.5 | 5.80 | 1.22 | 4.73E-03 |
| LBP_cg2085 | COG1893 [H] | 2-dehydropantoate 2-reductase | AGL64831.2 | 37.0 | 5.60 | 1.22 | 2.17E-03 |
| LBP_cg2268 | COG1827 [R] | Transcriptional regulator | AGL65014.2 | 18.7 | 7.20 | 1.22 | 7.36E-03 |
| LBP_cg0803 | COG1309 [K] | Transcription regulator | AGL63549.2 | 22.4 | 6.30 | 1.22 | 4.56E-04 |
| LBP_cg2837 | - | FMN-binding protein | AGL65583.2 | 13.4 | 6.10 | 1.21 | 1.54E-02 |
| LBP_cg1765 | COG0791 [M] | Extracellular protein, gamma-D-glutamate-meso-diaminopimelate muropeptidase (Putative) | AGL64511.2 | 48.3 | 9.09 | 1.21 | 1.56E-02 |
| LBP_cg0920 | - | hypothetical protein | AGL63666.2 | 13.5 | 6.27 | 1.21 | 3.41E-03 |
| LBP_cg2178 | COG2606 [S] | putative transcriptional regulator | AGL64924.2 | 18.6 | 8.91 | 1.21 | 1.29E-04 |
| LBP_cg1134 | COG0719 [O] | ABC transporter component, iron regulated (Putative) | AGL63880.2 | 52.6 | 5.67 | 1.21 | 2.13E-03 |
| LBP_cg2613 | COG0834 [ET] | Amino acid ABC superfamily ATP binding cassette transporter, binding protein | AGL65359.2 | 29.0 | 9.96 | 1.21 | 7.69E-03 |
| LBP_cg1412 | COG1211 [I] | D-ribitol-5-phosphate cytidylyltransferase | AGL64158.2 | 25.9 | 6.06 | 1.21 | 3.62E-02 |
| LBP_cg2334 | COG1668 [CP] | ABC transporter, permease protein (Putative) | AGL65080.2 | 44.9 | 9.99 | 1.21 | 1.91E-03 |
| LBP_cg1237 | COG0036 [G] | Ribulose-phosphate 3-epimerase | AGL63983.2 | 23.3 | 4.91 | 1.21 | 1.52E-02 |
| LBP_cg0502 | COG0222 [J] | 50S ribosomal protein L7/L12 | AGL63248.2 | 12.6 | 4.48 | 1.21 | 2.28E-02 |
| LBP_cg2627 | COG4619 [R] | ABC superfamily ATP binding cassette transporter, ABC protein | AGL65373.2 | 24.0 | 6.33 | 1.21 | 3.53E-03 |
| LBP_cg2939 | COG0681 [U] | Signal peptidase I | AJF17211.1 | 22.4 | 9.89 | 1.21 | 2.34E-02 |
| LBP_cg2648 | COG0735 [P] | Ferric uptake regulator | AGL65394.2 | 18.1 | 6.43 | 1.21 | 6.87E-04 |
| LBP_cg1601 | COG2740 [K] | hypothetical protein | AGL64347.2 | 11.2 | 9.58 | 1.20 | 2.49E-03 |
| LBP_cg1584 | COG1680 [V] | Serine-type D-Ala-D-Ala carboxypeptidase | AGL64330.2 | 43.3 | 9.80 | 1.20 | 1.76E-02 |
| LBP_cg1505 | - | hypothetical protein | AGL64251.2 | 15.5 | 9.39 | 1.20 | 9.83E-04 |
