# Supplementary material for: Adaptation of Lactobacillus plantarum to Ampicillin Involves Mechanisms That Maintain Protein Homeostasis
Source: mSystems. 2020 Jan 28;5(1):e00853-19. doi: 10.1128/mSystems.00853-19 (PMC6989132; doi:10.1128/mSystems.00853-19)
Supplement: TABLE S6 [file mSystems.00853-19-st006.docx]

**Table S6.** Down-regulated proteins of *L. plantarum* 1600g relative to *L. plantarum* P-8 grown in the presence of the ampicillin

| **Accession** | **COG category** | **Description** | **Protein ID** | **MW [kDa]** | **calc. pI** | **Fold Change** | **T test p value** |
| --- | --- | --- | --- | --- | --- | --- | --- |
| LBP_cg1882 | COG0323 [L] | DNA mismatch repair protein | AGL64628.2 | 74.1 | 6.24 | 0.83 | 5.91E-03 |
| LBP_cg1500 | COG0624 [E] | Succinyl-diaminopimelate desuccinylase | AGL64246.2 | 40.8 | 5.10 | 0.83 | 3.52E-02 |
| LBP_cg1897 | COG2894 [D] | Septum site-determining protein MinD | AGL64643.2 | 29.1 | 5.21 | 0.83 | 6.73E-04 |
| LBP_cg1079 | COG0744 [M] | Penicillin binding protein 2A | AGL63825.2 | 77.7 | 9.38 | 0.83 | 3.25E-03 |
| LBP_cg2671 | COG2365 [T] | Protein-tyrosine phosphatase | AGL65417.2 | 29.7 | 6.24 | 0.83 | 3.03E-02 |
| LBP_cg0793 | COG0028 [EH] | Acetolactate synthase | AGL63539.2 | 61.2 | 5.76 | 0.83 | 1.57E-03 |
| LBP_cg0696 | COG0518 [F] | Bifunctional GMP synthase/glutamine amidotransferase protein | AGL63442.2 | 57.4 | 5.16 | 0.83 | 7.90E-04 |
| LBP_cg1755 | COG1249 [C] | Dihydrolipoyl dehydrogenase | AGL64501.2 | 49.9 | 5.45 | 0.83 | 5.44E-03 |
| LBP_cg1608 | COG0750 [M] | Zinc-dependent protease, membrane associated (Putative) | AGL64354.2 | 45.8 | 9.06 | 0.83 | 5.18E-03 |
| LBP_cg0206 | COG2213 [G] | Protein-N(Pi)-phosphohistidine--sugar phosphotransferase | AGL62952.2 | 63.8 | 8.65 | 0.83 | 2.44E-02 |
| LBP_cg1959 | COG1680 [V] | putative beta-lactamase | AGL64705.2 | 38.0 | 9.60 | 0.83 | 3.60E-02 |
| LBP_cg1705 | COG1299 [G] | Fructose PTS, EIIABC | AGL64451.2 | 68.5 | 5.81 | 0.83 | 1.01E-03 |
| LBP_cg1247 | COG1196 [D] | Cell division protein Smc | AGL63993.2 | 131.8 | 5.55 | 0.82 | 5.95E-03 |
| LBP_cg1182 | COG1559 [R] | Aminodeoxychorismate lyase | AGL63928.2 | 44.2 | 9.55 | 0.82 | 1.51E-02 |
| LBP_cg1956 | COG0769 [M] | UDP-N-acetylmuramyl tripeptide synthase (Putative) | AGL64702.2 | 49.7 | 7.84 | 0.82 | 8.23E-03 |
| LBP_cg2282 | COG0561 [R] | HAD superfamily hydrolase | AGL65028.2 | 31.0 | 5.41 | 0.82 | 8.45E-05 |
| LBP_cg2273 | COG3048 [E] | putative D-serine dehydratase | AGL65019.2 | 47.6 | 5.82 | 0.82 | 2.31E-02 |
| LBP_cg1883 | COG0249 [L] | DNA mismatch repair protein mutS | AGL64629.2 | 100.0 | 5.50 | 0.81 | 8.67E-05 |
| LBP_cg0551 | COG0642 [T] | Sensor protein | AGL63297.2 | 52.6 | 6.61 | 0.81 | 1.19E-02 |
| LBP_cg2204 | COG1074 [L] | ATP-dependent nuclease, subunit A | AGL64950.2 | 140.9 | 5.29 | 0.81 | 1.79E-04 |
| LBP_cg0550 | COG0745 [TK] | Response regulator | AGL63296.2 | 27.2 | 5.97 | 0.81 | 3.31E-03 |
| LBP_cg2275 | COG2723 [G] | 6-phospho-beta-glucosidase | AGL65021.2 | 54.9 | 5.08 | 0.81 | 3.71E-03 |
| LBP_cg1618 | COG4123 [R] | Methyltransferase (Putative) | AGL64364.2 | 28.3 | 7.24 | 0.81 | 3.33E-04 |
| LBP_cg1922 | - | XRE family transcriptional regulator | AGL64668.2 | 33.4 | 6.74 | 0.81 | 2.15E-03 |
| LBP_cg2219 | - | hypothetical protein | AGL64965.2 | 33.0 | 7.36 | 0.81 | 8.84E-03 |
| LBP_cg2153 | - | hypothetical protein | AGL64899.2 | 32.2 | 6.74 | 0.80 | 8.93E-06 |
| LBP_cg1957 | COG3442 [R] | Cobyric acid synthase (Putative) | AGL64703.2 | 26.5 | 5.30 | 0.80 | 1.53E-02 |
| LBP_cg2254 | - | NUDIX family hydrolase | AGL65000.2 | 18.1 | 8.16 | 0.80 | 1.37E-04 |
| LBP_cg1756 | COG0508 [C] | Pyruvate dehydrogenase | AGL64502.2 | 46.6 | 5.15 | 0.80 | 3.36E-03 |
| LBP_cg2494 | COG0531 [E] | Amino acid transport protein | AGL65240.2 | 54.3 | 9.50 | 0.80 | 6.64E-03 |
| LBP_cg0321 | COG1028 [IQR] | Short chain dehydrogenase | AGL63067.2 | 32.8 | 9.86 | 0.80 | 9.75E-03 |
| LBP_cg0432 | COG1197 [LK] | Transcription-repair coupling factor | AGL63178.2 | 132.2 | 5.35 | 0.80 | 1.06E-04 |
| LBP_cg0763 | - | hypothetical protein | AGL63509.2 | 37.8 | 9.42 | 0.80 | 6.87E-03 |
| LBP_cg1783 | COG3599 [D] | Cell division initiation protein DivIVA | AGL64529.2 | 26.2 | 4.65 | 0.80 | 8.58E-04 |
| LBP_cg1752 | COG0483 [G] | Myo-inositol-1(Or 4)-monophosphatase | AGL64498.2 | 27.7 | 6.70 | 0.80 | 4.02E-03 |
| LBP_cg1563 | COG0124 [J] | Histidyl-tRNA synthetase | AGL64309.2 | 47.9 | 5.35 | 0.80 | 2.96E-03 |
| LBP_cg2750 | - | hypothetical protein | AGL65496.2 | 14.0 | 5.27 | 0.79 | 2.64E-05 |
| LBP_cg1379 | COG0564 [J] | Pseudouridine synthase | AGL64125.2 | 33.8 | 9.39 | 0.79 | 3.53E-03 |
| LBP_cg1913 | COG4493 [S] | hypothetical protein | AGL64659.2 | 23.5 | 8.13 | 0.79 | 1.09E-02 |
| LBP_cg2636 | COG1762 [GT] | hypothetical protein | AGL65382.2 | 17.5 | 5.17 | 0.79 | 8.69E-04 |
| LBP_cg0508 | COG0209 [F] | Ribonucleoside-diphosphate reductase | AGL63254.2 | 82.1 | 5.36 | 0.79 | 2.88E-03 |
| LBP_cg0324 | COG1125 [E] | ABC superfamily ATP binding cassette transporter, ABC protein | AGL63070.2 | 35.8 | 5.52 | 0.79 | 1.94E-03 |
| LBP_cg0929 | - | hypothetical protein | AGL63675.2 | 21.3 | 8.85 | 0.79 | 4.11E-03 |
| LBP_cg2233 | COG0026 [F] | Phosphoribosylaminoimidazole carboxylase ATPase subunit | AGL64979.2 | 40.5 | 6.47 | 0.79 | 3.51E-02 |
| LBP_cg0172 | - | XRE family transcriptional regulator | AGL62918.2 | 32.0 | 5.80 | 0.79 | 1.20E-04 |
| LBP_cg0661 | - | hypothetical protein | AGL63407.2 | 30.1 | 6.29 | 0.79 | 3.38E-02 |
| LBP_cg2277 | COG1440 [G] | Cellobiose PTS, EIIB | AGL65023.2 | 11.3 | 4.75 | 0.79 | 1.44E-02 |
| LBP_cg2131 | COG0778 [C] | Nitroreductase | AGL64877.2 | 20.0 | 5.81 | 0.79 | 1.51E-02 |
| LBP_cg1002 | COG0438 [M] | Poly(Glycerol-phosphate) alpha-glucosyltransferase | AGL63748.2 | 57.8 | 6.28 | 0.79 | 1.82E-03 |
| LBP_cg2258 | COG0498 [E] | Threonine synthase | AGL65004.2 | 54.5 | 6.35 | 0.79 | 1.41E-03 |
| LBP_cg1000 | - | hypothetical protein | AGL63746.2 | 63.1 | 8.59 | 0.79 | 1.01E-02 |
| LBP_cg1306 | - | hypothetical protein | AGL64052.2 | 9.5 | 9.45 | 0.79 | 1.48E-03 |
| LBP_cg2855 | COG2723 [G] | 6-phospho-beta-glucosidase | AGL65601.2 | 54.7 | 4.97 | 0.79 | 4.36E-03 |
| LBP_cg1172 | COG0419 [L] | Exonuclease SbcC | AGL63918.2 | 118.0 | 5.71 | 0.79 | 1.39E-05 |
| LBP_cg0566 | COG0446 [R] | NADH oxidase | AGL63312.2 | 51.5 | 5.24 | 0.78 | 1.00E-02 |
| LBP_cg2691 | COG0285 [H] | Folylpolyglutamate synthase | AGL65437.2 | 49.3 | 7.20 | 0.78 | 3.48E-04 |
| LBP_cg1556 | COG1806 [S] | Putative phosphotransferase | AGL64302.2 | 30.5 | 5.82 | 0.78 | 8.29E-04 |
| LBP_cg2760 | COG0656 [R] | Oxidoreductase | AGL65506.2 | 31.7 | 5.67 | 0.78 | 1.34E-02 |
| LBP_cg0954 | COG1023 [G] | 6-phosphogluconate dehydrogenase, decarboxylating | AGL63700.2 | 31.8 | 5.52 | 0.78 | 3.26E-03 |
| LBP_cg2142 | COG1154 [HI] | 1-deoxy-D-xylulose-5-phosphate synthase | AGL64888.2 | 63.7 | 5.73 | 0.78 | 4.68E-03 |
| LBP_cg2604 | COG1475 [K] | Chromosome partitioning protein, DNA-binding protein | AGL65350.2 | 31.7 | 9.00 | 0.78 | 4.96E-04 |
| LBP_cg2230 | COG0047 [F] | Phosphoribosylformylglycinamidine synthase 1 | AGL64976.2 | 23.9 | 5.01 | 0.78 | 2.49E-02 |
| LBP_cg2835 | COG1609 [K] | Galactose operon repressor | AGL65581.2 | 37.1 | 7.65 | 0.77 | 6.13E-05 |
| LBP_cg0231 | COG1887 [M] | Teichoic acid biosynthesis protein | AGL62977.2 | 47.0 | 9.07 | 0.77 | 9.79E-04 |
| LBP_cg2321 | COG1011 [R] | 2-haloacid dehalogenase (Putative) | AGL65067.2 | 25.3 | 5.76 | 0.77 | 3.81E-04 |
| LBP_cg0054 | COG0637 [R] | Beta-phosphoglucomutase | AGL62800.2 | 26.2 | 4.82 | 0.77 | 2.56E-03 |
| LBP_cg2278 | COG2188 [K] | Transcription regulator | AGL65024.2 | 26.9 | 7.21 | 0.77 | 4.75E-04 |
| LBP_cg2335 | COG4221 [R] | Short-chain dehydrogenase/oxidoreductase | AGL65081.2 | 26.0 | 6.58 | 0.77 | 1.07E-02 |
| LBP_cg0368 | COG1658 [L] | Ribonuclease M5 | AGL63114.2 | 20.2 | 8.38 | 0.77 | 6.28E-03 |
| LBP_cg0570 | COG1109 [G] | Phosphoglucomutase | AGL63316.2 | 63.5 | 5.11 | 0.77 | 1.35E-05 |
| LBP_cg1927 | COG0039 [C] | L-2-hydroxyisocaproate dehydrogenase | AGL64673.2 | 32.5 | 5.35 | 0.77 | 6.88E-03 |
| LBP_cg0555 | COG1117 [P] | Phosphate import ATP-binding protein pstB 1 | AGL63301.2 | 30.4 | 5.54 | 0.77 | 2.83E-03 |
| LBP_cg2489 | COG1473 [R] | Aminoacylase | AGL65235.2 | 43.0 | 5.99 | 0.76 | 5.66E-04 |
| LBP_cg2836 | COG0667 [C] | Aldo/keto reductase family protein | AGL65582.2 | 37.9 | 6.81 | 0.76 | 1.59E-02 |
| LBP_cg0353 | COG0042 [J] | tRNA-dihydrouridine synthase | AGL63099.2 | 39.5 | 7.17 | 0.76 | 2.25E-03 |
| LBP_cg2668 | COG0015 [F] | Adenylosuccinate lyase | AGL65414.2 | 49.0 | 5.97 | 0.76 | 1.23E-02 |
| LBP_cg0647 | COG0596 [R] | Proline iminopeptidase | AGL63393.2 | 35.3 | 5.50 | 0.75 | 7.36E-06 |
| LBP_cg1356 | COG0116 [L] | Site-specific DNA-methyltransferase | AGL64102.2 | 43.1 | 6.83 | 0.75 | 1.75E-04 |
| LBP_cg0108 | - | Transcription regulator | AGL62854.2 | 17.6 | 9.64 | 0.75 | 1.44E-04 |
| LBP_cg2681 | COG0620 [E] | hypothetical protein | AGL65427.2 | 42.1 | 5.91 | 0.75 | 4.07E-05 |
| LBP_cg2670 | COG0516 [F] | GMP reductase | AGL65416.2 | 35.4 | 6.87 | 0.75 | 3.48E-02 |
| LBP_cg1675 | - | hypothetical protein | AGL64421.2 | 32.7 | 5.33 | 0.75 | 3.16E-02 |
| LBP_cg1490 | - | hypothetical protein | AGL64236.2 | 13.3 | 5.48 | 0.75 | 1.19E-04 |
| LBP_cg0658 | COG0194 [F] | Guanylate kinase | AGL63404.2 | 21.7 | 6.89 | 0.74 | 3.97E-03 |
| LBP_cg1757 | COG0022 [C] | Pyruvate dehydrogenase complex, E1 component, beta subunit | AGL64503.2 | 35.5 | 4.84 | 0.74 | 1.04E-03 |
| LBP_cg1016 | COG1428 [F] | Deoxyguanosine kinase | AGL63762.2 | 24.9 | 4.97 | 0.74 | 3.34E-02 |
| LBP_cg2467 | COG1396 [K] | Transcription regulator | AGL65213.2 | 30.5 | 7.11 | 0.74 | 5.36E-03 |
| LBP_cg1570 | COG1385 [S] | Protein of hypothetical function DUF558 | AGL64316.2 | 26.9 | 8.44 | 0.74 | 2.82E-04 |
| LBP_cg1758 | COG1071 [C] | Pyruvate dehydrogenase complex, E1 component, alpha subunit | AGL64504.2 | 41.4 | 5.26 | 0.74 | 2.34E-03 |
| LBP_cg2861 | COG0477 [GEPR] | Sugar transport protein | AGL65607.2 | 52.3 | 9.07 | 0.73 | 5.82E-04 |
| LBP_cg0556 | COG1117 [P] | Phosphate import ATP-binding protein pstB 2 | AGL63302.2 | 28.0 | 5.34 | 0.73 | 5.28E-04 |
| LBP_cg2832 | COG3345 [G] | Alpha-galactosidase | AGL65578.2 | 83.6 | 5.39 | 0.73 | 3.25E-02 |
| LBP_cg0148 | COG2182 [G] | Maltose/maltodextrin ABC transporter, substrate binding protein | AGL62894.2 | 45.6 | 9.69 | 0.73 | 4.52E-02 |
| LBP_cg0070 | - | Transcription regulator | AGL62816.2 | 11.9 | 9.25 | 0.73 | 4.05E-05 |
| LBP_cg1126 | COG0773 [M] | UDP-N-acetylmuramate--L-alanine ligase | AGL63872.2 | 48.7 | 5.67 | 0.73 | 1.29E-05 |
| LBP_cg1029 | COG0561 [R] | HAD superfamily hydrolase | AGL63775.2 | 31.6 | 5.25 | 0.72 | 3.98E-02 |
| LBP_cg1374 | COG0665 [E] | Oxidoreductase | AGL64120.2 | 40.4 | 5.95 | 0.72 | 2.12E-04 |
| LBP_cg1183 | COG0572 [F] | Uridine kinase | AGL63929.2 | 24.0 | 5.67 | 0.72 | 1.41E-02 |
| LBP_cg2890 | COG0329 [EM] | N-acetylneuraminate lyase | AGL65636.2 | 32.7 | 4.91 | 0.72 | 1.84E-02 |
| LBP_cg0327 | COG0578 [C] | Glycerol-3-phosphate dehydrogenase | AGL63073.2 | 66.6 | 5.29 | 0.72 | 3.49E-02 |
| LBP_cg1080 | COG3679 [S] | hypothetical protein | AGL63826.2 | 13.3 | 4.70 | 0.71 | 9.36E-04 |
| LBP_cg1753 | COG4476 [S] | hypothetical protein | AGL64499.2 | 11.1 | 6.07 | 0.71 | 1.19E-04 |
| LBP_cg2946 | COG1847 [R] | hypothetical protein | AJF17218.1 | 34.2 | 9.73 | 0.71 | 8.76E-05 |
| LBP_cg1573 | - | hypothetical protein | AGL64319.2 | 8.6 | 10.24 | 0.71 | 1.29E-02 |
| LBP_cg1274 | COG1063 [ER] | Alcohol dehydrogenase | AGL64020.2 | 36.8 | 6.07 | 0.71 | 3.20E-03 |
| LBP_cg2274 | COG2723 [G] | 6-phospho-beta-glucosidase | AGL65020.2 | 54.7 | 5.29 | 0.71 | 5.38E-04 |
| LBP_cg0646 | COG0028 [EH] | Putative pyruvate oxidase | AGL63392.2 | 63.5 | 5.16 | 0.70 | 2.08E-05 |
| LBP_cg2168 | COG0095 [H] | Lipoate-protein ligase | AGL64914.2 | 38.2 | 5.10 | 0.70 | 2.14E-03 |
| LBP_cg2891 | COG3010 [G] | Putative N-acetylmannosamine-6-phosphate 2-epimerase | AGL65637.2 | 24.3 | 5.27 | 0.70 | 9.69E-05 |
| LBP_cg0153 | COG3839 [G] | Multiple sugar ABC transporter, ATP-binding protein | AGL62899.2 | 41.3 | 5.90 | 0.69 | 2.20E-02 |
| LBP_cg0093 | COG2145 [H] | Hydroxyethylthiazole kinase | AGL62839.2 | 27.5 | 4.75 | 0.69 | 4.58E-02 |
| LBP_cg2132 | COG1957 [F] | Purine nucleosidase | AGL64878.2 | 34.1 | 4.64 | 0.69 | 4.62E-03 |
| LBP_cg0269 | COG0282 [C] | Acetate kinase | AGL63015.2 | 43.8 | 6.48 | 0.69 | 2.48E-03 |
| LBP_cg0650 | - | X-prolyl-dipeptidyl aminopeptidase | AGL63396.2 | 91.5 | 5.54 | 0.69 | 2.33E-02 |
| LBP_p1g002 | COG1418 [R] | phosphohydrolase | AGL65641.2 | 25.3 | 6.67 | 0.69 | 2.08E-03 |
| LBP_cg0959 | - | hypothetical protein | AGL63705.2 | 38.2 | 7.31 | 0.69 | 3.12E-03 |
| LBP_cg1301 | COG2230 [M] | Cyclopropane-fatty-acyl-phospholipid synthase | AGL64047.2 | 44.6 | 6.58 | 0.68 | 7.81E-05 |
| LBP_cg1719 | COG1126 [E] | Glutamine ABC transporter, ATP-binding protein | AGL64465.2 | 26.9 | 4.86 | 0.68 | 2.92E-02 |
| LBP_cg1720 | COG0765 [E] | Glutamine ABC transporter, substrate binding and permease protein | AGL64466.2 | 53.8 | 9.82 | 0.68 | 2.54E-02 |
| LBP_cg1331 | COG0477 [GEPR] | Major facilitator superfamily permease | AGL64077.2 | 48.3 | 9.50 | 0.68 | 2.22E-02 |
| LBP_cg2911 | COG0028 [EH] | Pyruvate oxidase | AJF17183.1 | 66.1 | 5.57 | 0.67 | 7.34E-04 |
| LBP_p2g029 | COG3158 [P] | Potassium uptake protein | AGL65725.2 | 76.4 | 9.42 | 0.67 | 1.17E-03 |
| LBP_cg1332 | COG1554 [G] | Maltose phosphorylase | AGL64078.2 | 85.7 | 5.03 | 0.67 | 2.16E-02 |
| LBP_cg2605 | COG0357 [M] | Ribosomal RNA small subunit methyltransferase G | AGL65351.2 | 26.8 | 9.22 | 0.67 | 1.23E-04 |
| LBP_cg0095 | COG0352 [H] | Thiamine-phosphate pyrophosphorylase | AGL62841.2 | 22.8 | 5.14 | 0.67 | 1.79E-05 |
| LBP_cg1627 | COG0463 [M] | Glycosyltransferase | AGL64373.2 | 35.5 | 6.09 | 0.67 | 3.88E-05 |
| LBP_cg2878 | COG1762 [GT] | Galacitol PTS, EIIA | AGL65624.2 | 17.5 | 5.17 | 0.67 | 3.76E-02 |
| LBP_cg2669 | COG0104 [F] | Adenylosuccinate synthetase | AGL65415.2 | 47.2 | 5.64 | 0.66 | 2.24E-02 |
| LBP_cg2524 | COG0367 [E] | Asparagine synthase (Glutamine-hydrolysing) | AGL65270.2 | 73.0 | 6.73 | 0.66 | 1.31E-02 |
| LBP_cg2311 | COG1136 [V] | ABC transporter, ATP-binding protein | AGL65057.2 | 24.7 | 6.40 | 0.66 | 5.73E-03 |
| LBP_cg0610 | COG0708 [L] | Exodeoxyribonuclease III | AGL63356.2 | 29.6 | 5.34 | 0.66 | 1.48E-03 |
| LBP_cg1507 | - | hypothetical protein | AGL64253.2 | 63.9 | 5.30 | 0.66 | 4.95E-04 |
| LBP_cg0119 | COG4716 [S] | hypothetical protein | AGL62865.2 | 64.2 | 5.59 | 0.66 | 6.46E-04 |
| LBP_cg2854 | COG3711 [K] | Transcription antiterminator | AGL65600.2 | 32.8 | 6.40 | 0.66 | 4.94E-03 |
| LBP_cg1878 | - | Integral membrane protein | AGL64624.2 | 114.8 | 10.02 | 0.66 | 1.76E-03 |
| LBP_cg1454 | COG0488 [R] | ABC superfamily ATP binding cassette transporter, ABC protein | AGL64200.2 | 71.3 | 5.52 | 0.65 | 1.32E-02 |
| LBP_cg0495 | COG0267 [J] | 50S ribosomal protein L33 | AGL63241.2 | 5.7 | 9.60 | 0.65 | 4.56E-02 |
| LBP_p2g040 | COG1192 [D] | ATPase involved in chromosome partitioning | AGL65736.2 | 30.0 | 6.09 | 0.64 | 1.44E-04 |
| LBP_cg1333 | COG2017 [G] | Aldose 1-epimerase | AGL64079.2 | 36.6 | 6.06 | 0.64 | 1.73E-02 |
| LBP_cg0154 | COG1554 [G] | Maltose phosphorylase | AGL62900.2 | 86.5 | 5.48 | 0.64 | 1.23E-02 |
| LBP_cg1497 | COG0148 [G] | Enolase | AGL64243.2 | 46.6 | 5.03 | 0.64 | 3.25E-04 |
| LBP_cg0077 | - | hypothetical protein | AGL62823.2 | 16.0 | 5.01 | 0.64 | 1.15E-04 |
| LBP_cg0469 | COG1221 [KT] | Transcription regulator | AGL63215.2 | 105.2 | 5.67 | 0.64 | 9.33E-05 |
| LBP_cg1187 | COG4841 [S] | hypothetical protein | AGL63933.2 | 11.5 | 4.82 | 0.64 | 1.08E-04 |
| LBP_cg0426 | COG0078 [E] | Ornithine carbamoyltransferase | AGL63172.2 | 36.8 | 5.60 | 0.63 | 2.67E-02 |
| LBP_cg2555 | COG1455 [G] | Cellobiose PTS, EIIC | AGL65301.2 | 49.0 | 8.66 | 0.63 | 2.78E-04 |
| LBP_cg2231 | COG1828 [F] | Purine biosynthesis cluster protein | AGL64977.2 | 9.7 | 4.88 | 0.62 | 8.97E-03 |
| LBP_cg0983 | COG0503 [F] | Purine/pyrimidine phosphoribosyltransferase (Putative) | AGL63729.2 | 19.6 | 6.95 | 0.61 | 3.70E-02 |
| LBP_cg0146 | COG0366 [G] | Alpha-glucosidase | AGL62892.2 | 62.9 | 5.57 | 0.61 | 3.99E-02 |
| LBP_cg2227 | COG0150 [F] | Phosphoribosylformylglycinamidine cyclo-ligase | AGL64973.2 | 35.8 | 5.11 | 0.61 | 7.89E-03 |
| LBP_cg2375 | COG1511 [S] | Integral membrane protein | AGL65121.2 | 126.8 | 9.07 | 0.60 | 3.98E-03 |
| LBP_cg2226 | COG0299 [F] | Phosphoribosylglycinamide formyltransferase | AGL64972.2 | 20.6 | 6.11 | 0.60 | 3.99E-02 |
| LBP_cg1223 | COG1125 [E] | putative quaternary-amine-transporting ATPase | AGL63969.2 | 44.0 | 5.16 | 0.60 | 2.78E-06 |
| LBP_cg2635 | COG1445 [G] | Protein-N(Pi)-phosphohistidine--sugar phosphotransferase | AGL65381.2 | 11.4 | 9.07 | 0.60 | 1.00E-03 |
| LBP_cg0326 | COG0554 [C] | Glycerol kinase 1 | AGL63072.2 | 55.8 | 5.44 | 0.60 | 3.44E-03 |
| LBP_cg2225 | COG0138 [F] | Bifunctional phosphoribosylaminoimidazolecarboxamide formyltransferase/IMP cyclohydrolase | AGL64971.2 | 55.3 | 6.13 | 0.60 | 1.15E-02 |
| LBP_cg2706 | COG4989 [R] | Oxidoreductase | AGL65452.2 | 33.9 | 6.27 | 0.60 | 3.61E-03 |
| LBP_cg1459 | COG0561 [R] | HAD superfamily hydrolase | AGL64205.2 | 28.9 | 5.01 | 0.59 | 9.85E-04 |
| LBP_p2g039 | - | hypothetical protein | AGL65735.2 | 8.9 | 6.06 | 0.58 | 1.24E-02 |
| LBP_cg2876 | COG3775 [G] | Galactitol PTS, EIIC | AGL65622.2 | 45.2 | 9.47 | 0.58 | 4.56E-02 |
| LBP_cg2612 | COG1167 [KE] | putative Aromatic-amino-acid transaminase | AGL65358.2 | 45.3 | 6.24 | 0.56 | 3.29E-02 |
| LBP_cg2877 | COG3414 [G] | Galactitol PTS, EIIB | AGL65623.2 | 10.5 | 5.15 | 0.56 | 4.90E-02 |
| LBP_cg0974 | COG1080 [G] | Phosphoenolpyruvate-protein phosphatase | AGL63720.2 | 63.1 | 4.83 | 0.56 | 5.18E-04 |
| LBP_cg0179 | COG0667 [C] | Aryl-alcohol dehydrogenase family enzyme | AGL62925.2 | 37.0 | 7.03 | 0.55 | 1.43E-03 |
| LBP_cg2650 | - | hypothetical protein | AGL65396.2 | 8.7 | 11.80 | 0.54 | 6.80E-04 |
| LBP_cg2472 | COG2723 [G] | 6-phospho-beta-glucosidase | AGL65218.2 | 53.4 | 5.21 | 0.54 | 2.71E-04 |
| LBP_cg1224 | COG1174 [E] | Glycine betaine/carnitine/choline ABC transporter, permease protein | AGL63970.2 | 22.4 | 8.40 | 0.53 | 5.21E-05 |
| LBP_cg0152 | COG0366 [G] | Alpha-amylase | AGL62898.2 | 49.8 | 5.08 | 0.53 | 2.22E-02 |
| LBP_cg1225 | COG1732 [M] | Glycine betaine/carnitine/choline ABC superfamily ATP binding cassette transporter, substrate binding protein | AGL63971.2 | 34.8 | 9.45 | 0.53 | 2.26E-05 |
| LBP_cg0580 | COG0165 [E] | Argininosuccinate lyase | AGL63326.2 | 52.3 | 5.69 | 0.53 | 1.33E-02 |
| LBP_cg0621 | COG2200 [T] | Diguanylate cyclase/phosphodiesterase domain-containing protein | AGL63367.2 | 25.2 | 5.54 | 0.52 | 2.14E-03 |
| LBP_cg2856 | COG2723 [G] | 6-phospho-beta-glucosidase | AGL65602.2 | 55.2 | 5.21 | 0.52 | 1.21E-03 |
| LBP_cg0158 | COG1940 [KG] | Fructokinase | AGL62904.2 | 30.6 | 5.19 | 0.52 | 3.67E-05 |
| LBP_cg2229 | COG0046 [F] | Phosphoribosylformylglycinamidine synthase 2 | AGL64975.2 | 79.7 | 5.34 | 0.50 | 1.26E-02 |
| LBP_cg2554 | COG2723 [G] | 6-phospho-beta-glucosidase | AGL65300.2 | 54.3 | 5.41 | 0.48 | 1.68E-03 |
| LBP_cg1226 | COG1174 [E] | Glycine betaine/carnitine/choline ABC transporter, ATP-binding protein | AGL63972.2 | 23.0 | 9.76 | 0.48 | 8.64E-05 |
| LBP_cg0579 | COG0137 [E] | Argininosuccinate synthase | AGL63325.2 | 45.1 | 5.14 | 0.48 | 3.19E-02 |
| LBP_p2g010 | COG2190 [G] | GPH family glycoside-pentoside-hexuronide:cation symporter | AGL65706.2 | 69.8 | 7.64 | 0.46 | 2.64E-03 |
| LBP_cg2156 | COG0028 [EH] | Pyruvate oxidase | AGL64902.2 | 64.2 | 5.12 | 0.46 | 3.52E-03 |
| LBP_p2g030 | - | Transcription regulator | AGL65726.2 | 16.8 | 8.56 | 0.45 | 6.22E-03 |
| LBP_cg2232 | COG0152 [F] | Phosphoribosylaminoimidazole-succinocarboxamide synthase | AGL64978.2 | 27.1 | 6.24 | 0.45 | 7.26E-03 |
| LBP_cg2224 | COG0151 [F] | Phosphoribosylamine--glycine ligase | AGL64970.2 | 43.5 | 5.59 | 0.45 | 2.86E-02 |
| LBP_p1g033 | COG1192 [D] | Copy number control protein | AGL65672.2 | 30.1 | 5.43 | 0.42 | 1.26E-05 |
| LBP_p2g050 | COG1249 [C] | Pyridine nucleotide-disulfide oxidoreductase family protein | AJF17232.1 | 50.9 | 8.69 | 0.41 | 1.14E-05 |
| LBP_cg2862 | COG1501 [G] | Alpha-glucosidase | AGL65608.2 | 87.2 | 6.24 | 0.39 | 2.93E-06 |
| LBP_cg2228 | COG0034 [F] | Amidophosphoribosyltransferase | AGL64974.2 | 52.3 | 6.04 | 0.39 | 1.15E-02 |
| LBP_p2g005 | COG3250 [G] | Beta-galactosidase large subunit | AGL65701.2 | 72.1 | 4.94 | 0.39 | 4.36E-02 |
| LBP_p2g025 | COG1307 [S] | hypothetical protein | AGL65721.2 | 30.6 | 9.23 | 0.35 | 6.17E-06 |
| LBP_p2g004 | COG3250 [G] | Beta-galactosidase | AGL65700.1 | 35.2 | 4.86 | 0.28 | 3.35E-04 |
| LBP_cg1351 | COG0744 [M] | Penicillin binding protein 1A | AGL64097.2 | 83.0 | 9.39 | 0.27 | 2.77E-06 |
| LBP_p1g016 | COG1961 [L] | Resolvase | AGL65655.2 | 22.1 | 9.99 | 0.27 | 9.99E-05 |
| LBP_p2g047 | - | hypothetical protein | AJF17229.1 | 7.9 | 9.99 | 0.23 | 7.29E-05 |
| LBP_p1g047 | COG2190 [G] | PTS sugar transporter subunit IIA | AGL65686.2 | 71.5 | 8.15 | 0.21 | 1.26E-03 |
| LBP_p1g007 | COG0662 [G] | Cupin 2 conserved barrel domain protein | AGL65646.2 | 16.2 | 5.47 | 0.14 | 3.18E-03 |
