# Supplementary material for: Adaptation of Lactobacillus plantarum to Ampicillin Involves Mechanisms That Maintain Protein Homeostasis
Source: mSystems. 2020 Jan 28;5(1):e00853-19. doi: 10.1128/mSystems.00853-19 (PMC6989132; doi:10.1128/mSystems.00853-19)
Supplement: TABLE S7 [file mSystems.00853-19-st007.docx]

**Table S7.** Signature peptides used for PRM validation of the up-regulated proteins in *L. plantarum* 400g and *L. plantarum* 1600g

| **Gene locus** | **Signature peptide** |
| --- | --- |
| LBP_cg0109 | TDINETDDQYQVK |
| LBP_cg0397 | GNLYEYDLDR |
| LBP_cg0720 | FVHVDLAAVSQK |
| LBP_cg0720 | LSEYLSK |
| LBP_cg0721 | VQQDVER |
| LBP_cg0721 | DEADPTQGVDAQVGK |
| LBP_cg0722 | NGQEILAR |
| LBP_cg0885 | SEFSNADELTNLEAVVK |
| LBP_cg0885 | VQTIDEQATQAYAQFK |
| LBP_cg1290 | ATLEGDFVNTTK |
| LBP_cg1290 | VNAISAGAVK |
| LBP_cg1290 | TLAVTGIHEHQQLLK |
| LBP_cg1294 | SIEVFTK |
| LBP_cg1294 | IIGEHLK |
| LBP_cg1793 | QSISSGTVVSSGQR |
| LBP_cg1793 | LTTSGTGYVK |
| LBP_cg2704 | VEVLPYHTLGVK |
| LBP_cg2704 | LEGIESPTQDR |
| LBP_cg2905 | NTIIIATSNAGFGNEALSGDK |
| LBP_cg2905 | LIGTSAGYVGYEDNANTLTER |
