# Supplementary material for: Adaptation of Lactobacillus plantarum to Ampicillin Involves Mechanisms That Maintain Protein Homeostasis
Source: mSystems. 2020 Jan 28;5(1):e00853-19. doi: 10.1128/mSystems.00853-19 (PMC6989132; doi:10.1128/mSystems.00853-19)
Supplement: TABLE S8 [file mSystems.00853-19-st008.docx]

**Table S8** Primers used for SNPs validation.

| **Primers** | **Primer sequence (5’-3’)** | **Annealing temperature (℃)** | **Amplicon size (bp)** |
| --- | --- | --- | --- |
| 1F | GGCGGAATAGATGAAGATAAGGC | 58.00 | 395 |
| 1R | GTGGAATGCGGAATGTTTAGTTG | 56.21 |  |
| 2F | GGAGCCTATGCTGTTGTAATGAA | 56.21 | 554 |
| 2R | GTGTAAGCATCGTAGTTCCCGTA | 58.00 |  |
| 3F | ATCGTCGTCGCTTTAGTCTCACT | 58.00 | 751 |
| 3R | AGATCGGTAATAATACCGTTCCT | 54.43 |  |
| 4F | TGGAACAAAGCGGAAGACGGGATT | 59.88 | 429 |
| 4R | GTTGCCATGCGAAATTGGAGGAT | 58.00 |  |
| 5F | TGCCCTCGTCGTCTTTATGTTAG | 58.00 | 361 |
| 5R | ACCAATCCGATAGCCTTTGTCTT | 56.21 |  |
| 6F | TCCCATGACTAATAAACTAAAGGC | 54.76 | 213 |
| 6R | TACGATGGTGAAGTGACGATGTA | 56.21 |  |
